# Preferential targeting of i-motifs and G-quadruplexes by small molecules†
†Electronic supplementary information (ESI) available: Experimental details, synthetic procedures, characterization data of compounds, ^1^H NMR and ^13^C NMR spectra, FRET melting, TO displacement, smFRET, lifetime data, CD spectra, western blot, dual luciferase, caspase assay. See DOI: 10.1039/c7sc02693e



**DOI:** 10.1039/c7sc02693e

**Published:** 2017-09-08

**Authors:** Manish Debnath, Shirsendu Ghosh, Ajay Chauhan, Rakesh Paul, Kankan Bhattacharyya, Jyotirmayee Dash

**Affiliations:** a Department of Organic Chemistry , Indian Association for the Cultivation of Science , Jadavpur , Kolkata-700032 , India . Email: ocjd@iacs.res.in; b Department of Physical Chemistry , Indian Association for the Cultivation of Science , Jadavpur , Kolkata-700032 , India

## Abstract

i-Motifs and G-quadruplexes are dynamic nucleic acid secondary structures, which are believed to play key roles in gene expression. We herein report two peptidomimetic ligands (**PBP1** and **PBP2**) that selectively target i-motifs and G-quadruplexes over double-stranded DNA. These peptidomimetics, regioisomeric with respect to the position of triazole/prolinamide motifs, have been synthesized using a modular method involving Cu(i)-catalyzed azide and alkyne cycloaddition. The *para*-isomer, **PBP1** exhibits high selectivity for i-motifs while the *meta*-isomer **PBP2** binds selectively to G-quadruplex structures. Interestingly, these ligands have the ability to induce G-quadruplex or i-motif structures from the unstructured single-stranded DNA conformations, as observed using single molecule Förster resonance energy transfer (smFRET) studies. The quantitative real-time polymerase chain reaction (qRT-PCR), western blot, and dual-luciferase assays indicate that **PBP1** upregulates and **PBP2** downregulates *BCL-2* gene expression in cancer cells.

## Abstract 2

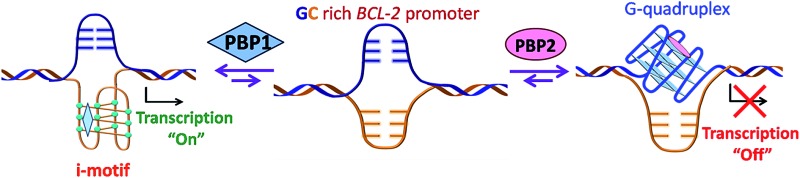
Ligand-dependent regulation of gene expression has been delineated by targeting i-motifs and G-quadruplexes.

## Introduction

Cytosine (C)-rich and guanine (G)-rich sequences can adopt stable nucleic acid secondary structures such as i-motifs^1^ and G-quadruplexes,^2^ respectively. The C-rich sequences form i-motif structures at acidic pH,^3–6^ whereas the G-rich sequences usually form G-quadruplexes at neutral pH in the presence of metal ions (Na^+^, K^+^). These sequences are prevalent in the promoter region of oncogenes like *BCL-2* and *c-MYC*.^7–10^ It has been reported that small molecules bind G-quadruplexes^11^ and modulate the gene expression.^12–17^ Although i-motifs are hypothesized to play important roles in gene transcription,^18–21^ only a few ligands are known to selectively target i-motifs in biological systems.^18–22^ Furthermore, i-motifs and G-quadruplexes are highly dynamic and they can exist in equilibrium with unfolded DNA under physiological conditions.^12,18–20^ However, little is known about how small molecules can regulate the relative populations of these two dynamic secondary structures. In this context, we envisioned to develop small molecules that can discriminate between i-motif and G-quadruplex structures and modulate gene expression.

The single molecule Förster resonance energy transfer (smFRET) technique provides key information about the structure, relative population distribution of folded or unfolded species, and the end-to-end distance of biomolecules.^23–34^ The smFRET technique has been used to elucidate the conformational dynamics of G-quadruplexes in the presence of metal ions (K^+^/Na^+^),^28^ protein,^33^ and small molecules.^34^ The population equilibrium of C-rich ILPR and *BCL-2* promoter sequences has been studied using laser tweezer experiments.^35,36^ Majima and co-workers have used smFRET to quantitatively analyse the pH-induced intra-molecular folding dynamics of i-motif DNA.^37^ However, the use of smFRET to monitor the ligand induced change in the relative population distribution of i-motif and G-quadruplex structures present in oncogenic promoters is very limited.

Hurley and Hecht have reported that the steroid ligand **IMC-48** folds the *BCL-2* C-rich sequence into an i-motif, while the same sequence is folded into a hairpin duplex in the presence of the related ligand **IMC-76**.^18,19^ In this study, we describe the synthesis of two flexible peptidomimetic congeners, **PBP1** and **PBP2**, which show structure-specific recognition for G-quadruplex and i-motif structures. The interaction of these ligands with *BCL-2* or *c-MYC* i-motifs and G-quadruplexes has been evaluated using biophysical studies like melting analysis by Förster resonance energy transfer (FRET), thiazole-orange (TO) displacement assay, fluorescence quenching assay, and circular dichroism (CD) spectroscopy. In addition, the ability of these ligands to induce the formation of i-motif and G-quadruplex structures from the unfolded *BCL-2* and *c-MYC* C-rich and G-rich promoter sequences has been investigated using smFRET and fluorescence lifetime studies at neutral pH. We have further demonstrated how ligand-dependent conformational changes of *BCL-2* i-motif or G-quadruplex topologies can modulate the *BCL-2* expression in cancer cells.

## Results and discussion

### Design and synthesis of peptidomimetic ligands

Peptidomimetics are designed to interact with specific biological targets as they exhibit enhanced proteolytic stability and improved cell permeability.^38,39^ We have anticipated that peptidomimetics containing the 2,6-pyridine dicarboxamide unit, linked to l-proline residues through triazole and arene motifs would be structurally flexible enough to adopt different conformations upon interacting with different DNA four stranded structures (i-motifs and G-quadruplexes). The proline residues play an important role in peptide conformation. The 2,6-pyridine dicarboxamide motif can adopt folded conformations due to the bifurcated H-bonding between the lone pair of pyridine nitrogen and amide –NH protons. The arene motif attached to the proline residues would provide additional flexibility to form topologically different positional isomers that could discriminate between different DNA structures such as i-motifs and G-quadruplexes (Fig. S1, ESI†). Furthermore, the triazole ring system could facilitate stacking interactions with the loop bases and, thus, could differentially interact with different DNA secondary structures with variations in the loop region.^40^ The triazole ring system, able to mimic the *cis*- or *trans*-conformations of amide bonds, would impart rigidity to the peptidomimetics. It has been reported that triazole containing ligands generated by “click” chemistry selectively bind G-quadruplexes.^41–43^

**Scheme 1:**
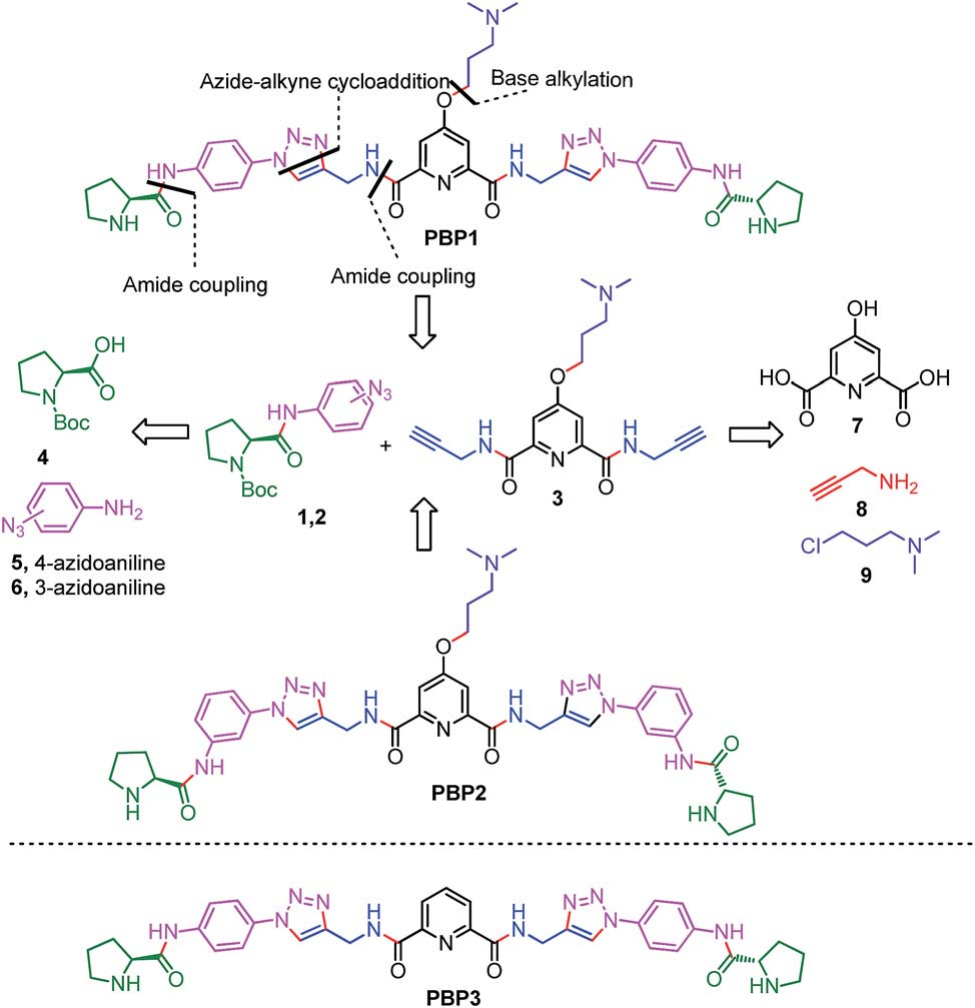
The synthesis of bis-prolinamide derivatives **PBP1**, **PBP2** and the structure of **PBP3**.

The bis-triazole containing peptidomimetic type ligands **PBP1** and **PBP2** were assembled using a modular synthetic strategy involving a Cu(i)-catalyzed 1,3-dipolar azide–alkyne cycloaddition between azido prolinamides **1**, **2** and pyridyl dialkyne **3** (Schemes 1 and S1, ESI†). The azido prolinamides **1** and **2** were obtained by amide coupling of *N*-Bocproline **4** with the *para* and *meta*-azido anilines **5** and **6**. The dialkyne building block **3** was prepared from chelidamic acid **7**. Chelidamic acid **7** was treated with oxalyl chloride to generate the corresponding acid chloride, which was subsequently coupled with propargyl amine **8**, followed by alkylation of the resulting pyridyl dialkyne with 3-dimethylaminopropyl chloride **9**, affording the dialkyne **3** in high overall yield. The Cu(i)-catalyzed Huisgen cycloaddition of azido prolinamide derivatives **1** and **2** with the dialkyne **3** and subsequent removal of the Boc group provided the bis-prolinamide derivatives **PBP1** and **PBP2** in high yields. The bis-prolinamide derivative **PBP3** was similarly assembled from azido prolinamide **1** and pyridine-2,6-dicarboxylic acid.

### **PBP1** and **PBP2** exhibit differential binding between i-motifs and G-quadruplexes

The ability of these regioisomeric ligands to interact with G-quadruplexes and i-motifs was evaluated using biophysical assays. C-Rich sequences were folded into i-motifs by annealing in 60 mM K-cacodylate buffer, at pH 4.8 and then the pH was adjusted to 6 for biophysical analysis.^18,22,44,45^

#### (a) Melting analysis using FRET

**Fig. 1:**
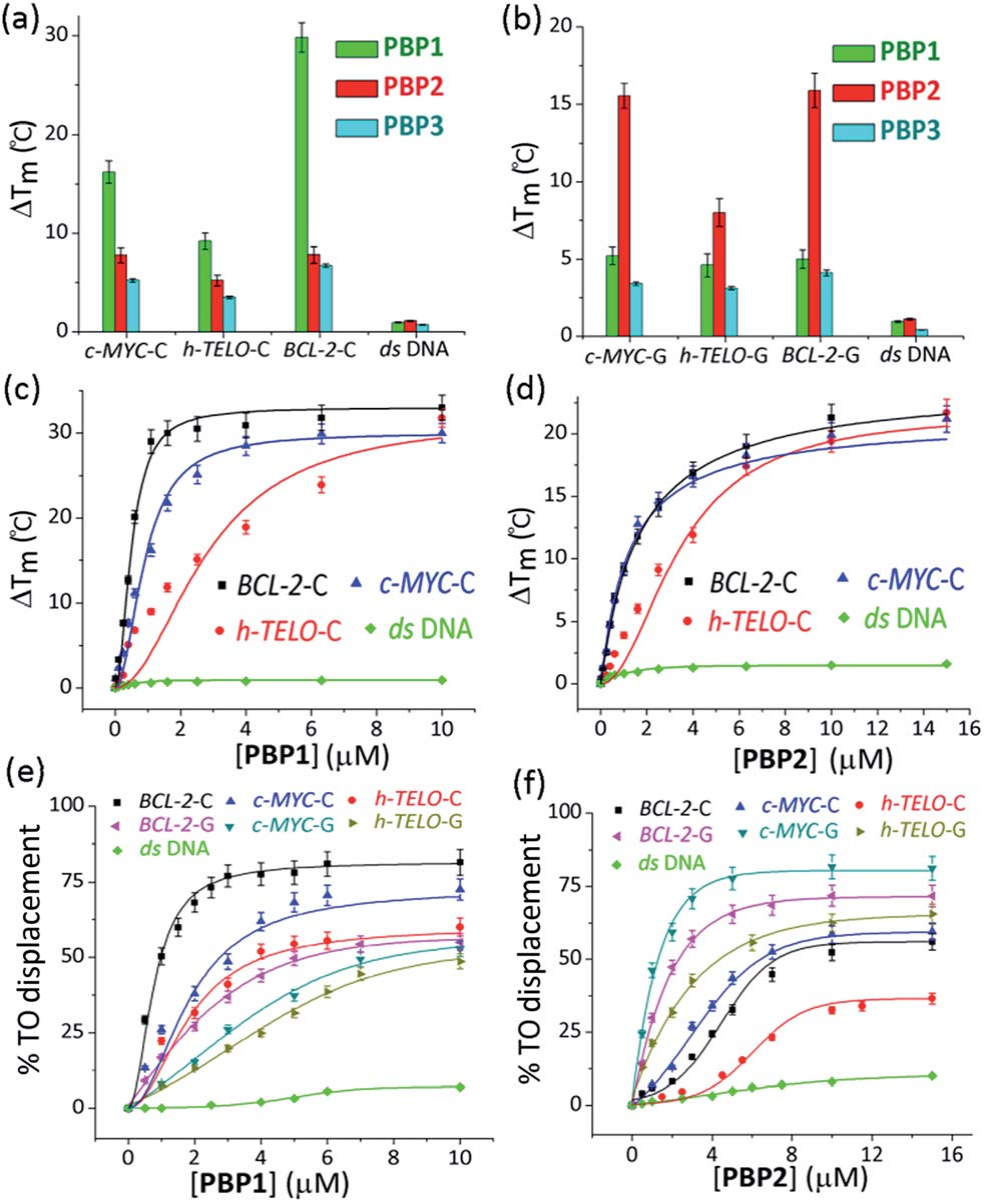
The FRET melting and TO displacement assays. The FRET stabilization potential of bis-prolinamide derivatives **PBP1** (1 μM), **PBP2** (1 μM), and **PBP3** (1 μM) upon interaction with (a) 100 nM folded i-motifs (*c-MYC*-C, *BCL-2*-C, and *h-TELO*-C) and *ds* DNA in 60 mM K-cacodylate buffer, (pH 6); (b) 100 nM folded G-quadruplexes (*c-MYC*-G, *BCL-2*-G, and *h-TELO*-G) and *ds* DNA in 60 mM K-cacodylate buffer, (pH 7); thermal shift profiles for (c) **PBP1** (0–10 μM) and (d) **PBP2** (0–15 μM) upon stabilizing i-motifs and *ds* DNA in 60 mM K-cacodylate buffer, (pH 6). The TO displacement from 250 nM *BCL-2*-C, *c-MYC*-C, and *h-TELO*-C i-motifs in 60 mM K-cacodylate buffer, (pH 6); *BCL-2*-G, *c-MYC*-G, and *h-TELO*-G G-quadruplexes and *ds* DNA in 60 mM K-cacodylate buffer, (pH 7) with increasing concentrations of (e) **PBP1** (0–10 μM); (f) **PBP2** (0–15 μM).

The FRET based melting assay was carried out to evaluate the stabilization potential of **PBP1–3** for G-quadruplexes and i-motifs.^46,47^ Dual labeled (5′-FAM and 3′-TAMRA) C-rich and G-rich sequences present in oncogenic promoter regions (*BCL-2* and *c-MYC*) and the telomeric region (*h-TELO*) were folded into i-motifs and G-quadruplexes, respectively,^18,48^ and these were used in this study along with a control double-stranded (*ds*) DNA (Fig. 1 and Table S1, ESI†).

Interestingly, the two positional isomers **PBP1** and **PBP2** exhibited a marked difference in increasing the *T*_m_ of folded G-quadruplexes and i-motifs at 1 μM ligand concentration (Table 1, Fig. 1a and b and S2, ESI†). Ligand **PBP1**, in which the prolinamide motifs are at the *para* position with respect to the triazole ring system, increased the *T*_m_ values of *BCL-2*-C and *c-MYC*-C i-motifs more effectively compared to ligand **PBP2** at 1 μM ligand concentration (Δ*T*_m_ = 16–29 °C for **PBP1** and Δ*T*_m_ = 8 °C for **PBP2**). In contrast, the *meta* regioisomer **PBP2** increased the Δ*T*_m_ value of *c-MYC*-G and *BCL-2*-G G-quadruplexes (Δ*T*_m_ = 16 °C at 1 μM **PBP2** and Δ*T*_m_ = 5.2 °C at 1 μM **PBP1**) (Table 1). Ligand **PBP3**, which lacks the –NMe_2_ side chain in the central pyridine ring, showed low stabilization potential (Δ*T*_m_ = 3–5 °C) for both G-quadruplex and i-motif structures (Table S1, ESI†). When *BCL-2*-C and *c-MYC*-C mutant C-rich sequences were used in the melting analysis, no melting curves were observed, thereby indicating their existence in the unfolded form (Fig. S3, ESI†).

**Table 1:** The sequences used in this study and a comparison of the binding data obtained for **PBP1** and **PBP2** from TO displacement, fluorescence quenching, and FRET melting assay

DNA^*a*^	DC_50_ ^*b*^ (μM)		*K*_d_ ^*c*^ (μM)		Δ*T*_m_ ^*d*^ (°C)	
	**PBP1**	**PBP2**	**PBP1**	**PBP2**	**PBP1**	**PBP2**
*BCL-2*-C: 5′-d(CAGC_4_GCTC_3_GC_5_T_2_C_2_TC_3_GCGC_3_GC_4_T)-3′	0.9	8.2	0.3	5.8	29	8
*BCL-2*-G: 5′-d(AG_4_CG_3_CGCG_3_AG_2_A_2_G_5_CG_3_AGCG_4_CGT)-3′	5.7	2.4	7.2	1.9	5.2	16
*c-MYC*-C: 5′-d(TC_4_AC_2_T_2_C_4_AC_3_TC_4_AC_3_TC_4_A)-3′	2.7	6.8	2.4	9.5	16	8
*c-MYC*-G: 5′-d(TG_4_AG_3_TG_4_AG_3_TG_4_A_2_G_2_TG_4_A)-3′	8.5	1.3	12.5	1.3	5.2	16
*h-TELO*-C: 5′-d(TA_2_C_3_TA_2_C_3_TA_2_C_3_TA_2_C_3_)-3′	4	>15	n.d.	n.d.	9	5
*h-TELO*-G: 5′-d(G_3_TTAG_3_TTAG_3_TTAG_3_)-3′	9.8	4.7	n.d.	n.d.	5	8
*ds* DNA: 5′-d(TATAGCTATA-HEG-TATAGCTATA)-3′	n.d.	n.d.	>25	>25	0.94	1.1

^*a*^Unlabeled, single TAMRA labeled and dual FAM-TAMRA labeled sequences were used in the TO displacement, fluorescence quenching, and FRET melting experiments, respectively; HEG = hexaethylene glycol.

^*b*^Error = ±5%.

^*c*^ *K*_d_ values indicated for the 5′-labeled sequences (*K*_d_ = ± 5%). **PBP1** (fold selectivity): *BCL-2*-C/*c-MYC*-C/*BCL-2*-G/*c-MYC*-G = 40/6/1.5/1; **PBP2** (fold selectivity): *BCL-2*-C/*c-MYC*-C/*BCL-2*-G/*c-MYC*-G = 1.5/1/4.5/7.

^*d*^Δ*T*_m_ = ±1 °C; [**PBP1**] = [**PBP2**] = 1 μM. The *T*_m_ values of folded *c-MYC*-C, *BCL-2*-C, *h-TELO*-C i-motifs and *ds* DNA diluted in 60 mM K-cacodylate buffer at pH 6 are 48 ± 1 °C, 59 ± 1 °C, 43 ± 1 °C, and 60 ± 1 °C (Table S1, ESI). The *T*_m_ values of folded *c-MYC*-G, *BCL-2*-G, and *h-TELO*-G diluted in 60 mM K-cacodylate buffer at pH 7 are 69 ± 1 °C, 70 ± 1 °C, 55 ± 1 °C.

Next, FRET melting experiments were carried out for *BCL-2*, *c-MYC*, and *h-TELO* i-motifs and G-quadruplexes using an increasing concentration of **PBP1–2**. **PBP1** showed high Δ*T*_m_ values for *BCL-2*-C, *c-MYC*-C, and *h-TELO*-C i-motifs while **PBP2** exhibited high Δ*T*_m_ values for the corresponding G-quadruplexes in a dose-dependent manner (Fig. 1c and d, S2–S4, ESI†). **PBP1** showed a Δ*T*_m_ value of 32 ± 1 °C (*i.e.*, a *T*_m_ of 92 °C) for *BCL-2*-C at 1.3 μM concentration, whereas higher concentrations of **PBP1** were required to attain a Δ*T*_m_ value of 32 ± 1 °C for *c-MYC*-C (a *T*_m_ of 81 °C at 6.5 μM) and *h-TELO*-C (a *T*_m_ of 76 °C at 10 μM). These results indicate that **PBP1** shows a preferential affinity for the *BCL-2*-C i-motif as it can obtain a maximum Δ*T*_m_ value for the *BCL-2*-C i-motif at 5–8 fold lower concentrations than the *c-MYC*-C and *h-TELO*-C i-motifs. In contrast, *BCL-2*-G and *c-MYC*-G G-quadruplexes exhibited maximum Δ*T*_m_ values at 3 fold lower concentrations of **PBP2** than **PBP1** (Fig. S2, ESI†). However, both **PBP1** and **PBP2** failed to alter the *T*_m_ of *ds* DNA even at high ligand concentrations (10–15 μM), indicating their selectivity for four stranded structures over double-stranded DNA.

The selectivity of **PBP1** for i-motifs and **PBP2** for G-quadruplexes was determined using the FRET competition assay with the competing G-quadruplex (TG_5_T)_4_ and double-stranded ds26 DNA (Fig. S2c and d, ESI†). The results show that no significant changes in the Δ*T*_m_ values of **PBP1** bound i-motifs and **PBP2** bound G-quadruplexes were observed in the presence of 40 mol equivalent excess of the G-quadruplex and double-stranded DNA competitors.

#### (b) TO displacement assay

The affinity of **PBP1–3** for the folded G-quadruplexes and i-motifs was further investigated by measuring the ability of the ligands to displace the bound thiazole-orange (TO) from pre-folded G-quadruplexes^49^ or i-motifs^50^ (Fig. 1e and f). Table S2† lists the concentrations of the ligands required to displace TO by 50% (DC_50_) from the investigated DNA structures.

**PBP1** exhibited DC_50_ values of 0.9 μM, 2.7 μM and 4.0 μM for *BCL-2*-C, *c-MYC*-C and *h-TELO*-C i-motifs, respectively (Table 1). In comparison, the *meta*-isomer **PBP2** displayed significantly lower affinity for *BCL-2*-C (DC_50_ = 8.2 μM), *c-MYC*-C (DC_50_ = 6.8 μM), and *h-TELO*-C (DC_50_ > 15 μM) i-motifs. On the other hand, **PBP1** showed higher DC_50_ values for *BCL-2*-G, *c-MYC*-G, and *h-TELO*-G G-quadruplexes compared to **PBP2** (Table 1). These results are in agreement with the FRET melting data suggesting the higher affinity of **PBP1** for the *BCL-2*-C i-motif as compared to that of **PBP2** and the preferential binding of **PBP2** for *c-MYC*-G and *BCL-2*-G G-quadruplexes as compared to **PBP1**. However, ligand **PBP3** exhibited high DC_50_ values for G-quadruplexes (DC_50_ = 8.4–10.2 μM) and i-motifs (DC_50_ = 7.9–10 μM), which indicates the weak affinity of **PBP3** for both four stranded structures (Fig. S5, ESI†).

#### (c) Fluorescence binding titrations

**Fig. 2:**
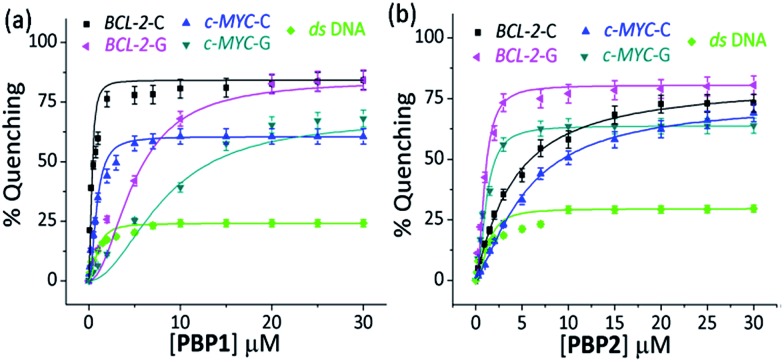
The percentage fluorescence quenching observed upon titration of 250 nM of 5′-TAMRA labeled folded *BCL-2*-C and *c-MYC*-C i-motif structures in 60 mM K-cacodylate buffer, pH 6, and 250 nM 5′-TAMRA labeled folded *BCL-2*-G and *c-MYC*-G G-quadruplex structures in 60 mM K-cacodylate buffer, pH 7 by (a) 0–30 μM **PBP1** and (b) 0–30 μM **PBP2**.

Next, fluorescence spectroscopy was employed to determine the dissociation constants (*K*_d_) of **PBP1** and **PBP2** with *BCL-2* and *c-MYC* i-motifs and G-quadruplexes (Tables S3 and S4, ESI†). Here, i-motifs and G-quadruplexes are labeled at either the 5′-end or at 3′-end with TAMRA dye. Binding of the ligand in the vicinity of the labeled site facilitates proximity induced quenching of the dye through non radiative methods (Scheme S2, ESI†).^51^ For a comparison, *ds* DNA was used as a control. We observed a dose-dependent decrease in the fluorescence emission of TAMRA labeled DNA structures upon titration with **PBP1** and **PBP2** (Fig. 2 and S6, ESI†). From the level of quenching, *K*_d_ values of the ligands for the i-motif and G-quadruplex structures were determined. **PBP1** showed a 20 fold higher affinity for the 5′-TAMRA-*BCL-2*-C i-motif with a *K*_d_ value of 0.3 μM over **PBP2** (*K*_d_ = 5.8 μM) (Table 1). Similarly, **PBP1** exhibited a lower *K*_d_ value (2.4 μM) for the 5′-TAMRA-*c-MYC*-C i-motif compared to **PBP2** (*K*_d_ = 9.5 μM).

When 5′-TAMRA labeled *BCL-2*-G and *c-MYC*-G G-quadruplexes were titrated with **PBP1** and **PBP2**, a marked difference in their affinity was observed. **PBP2** exhibited a 7 fold preference for the 5′-labeled *c-MYC*-G (*K*_d_ = 1.3 μM) G-quadruplex over the i-motif counterpart. Similarly, a 3 fold higher affinity of **PBP2** was observed for the *BCL-2*-G G-quadruplex (*K*_d_ = 1.9 μM) over the *BCL-2*-C i-motif. It is intriguing to note that **PBP1** showed a 24 fold higher selectivity for the *BCL-2*-C i-motif over *BCL-2*-G (*K*_d_ = 7.2 μM) and a 40 fold higher selectivity for the *BCL-2*-C i-motif over *c-MYC*-G (*K*_d_ = 12.5 μM) G-quadruplexes. To the best of our knowledge, this is one of the highest levels of selectivity reported by a small molecule ligand for i-motif over G-quadruplex structures.

Similar binding titrations with 3′-TAMRA labeled *BCL-2* and *c-MYC* i-motifs and G-quadruplexes revealed that both **PBP1** and **PBP2** displayed a higher affinity (lower *K*_d_ value) for 5′-labeled G-quadruplexes and i-motifs over 3′-labeled structures (Fig. S6, Scheme S2, Tables S3 and S4, ESI†). Therefore, the 5′-end of G-quadruplex and i-motif structures is the preferred binding site for **PBP1** and **PBP2**. In comparison, **PBP3** induced a considerably lower level of fluorescence quenching (>40%) in TAMRA labeled G-quadruplexes and i-motifs (Fig. S7, ESI†), suggesting the weak affinity of **PBP3** for these DNA structures. The weak affinity of **PBP3** may be attributed to its poor solubility in aqueous buffer and the lack of a cationic side chain, and hence **PBP3** was not selected for further studies. Importantly, **PBP1** and **PBP2** preferentially bind to the four stranded DNA structures over *ds* DNA, as control experiments with TAMRA labeled *ds* DNA showed no significant quenching upon addition of the ligands (Fig. 2 and S6, ESI†).

### **PBP1** and **PBP2** induce the formation of i-motifs and G-quadruplexes, respectively

#### (a) SmFRET analysis

**Fig. 3:**
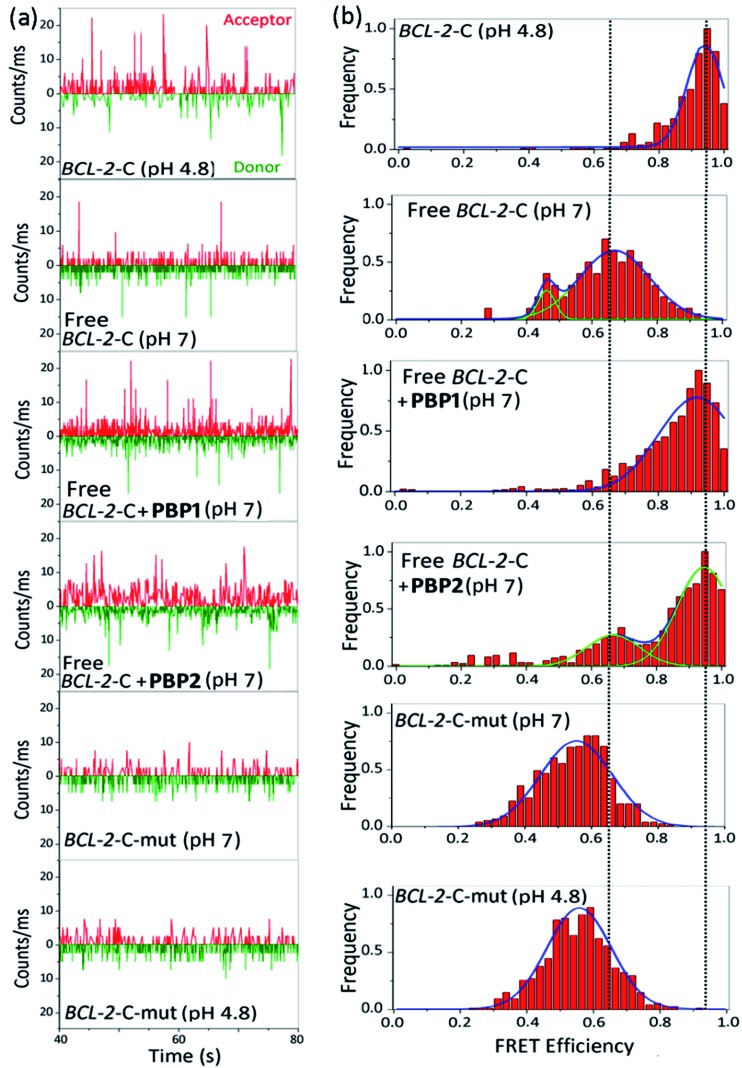
The smFRET analysis of *BCL-2*-C and mutated *BCL-2*-C (*BCL-2*-C-mut). Photon bursts of donor/acceptor (background corrected) (a), and FRET efficiency distributions (b) of 100 pM dual fluorescent labeled *BCL-2*-C and *BCL-2*-C-mut under neutral (pH 7) and acidic (pH 4.8) conditions or in the presence of **PBP1** (1 equiv.), **PBP2** (1 equiv.). *BCL-2*-C-mut: 5′-FAM-d(CAGC_2_TCGCTC_2_TGC_2_TC_2_T_2_C_2_TC_2_TGCGC_2_TGC_2_TCG)-TAMRA-3′.

SmFRET was used to study the conformational changes of folded and free i-motif and G-quadruplex forming sequences in the presence and absence of ligands *via* monitoring the FRET between donor and acceptor fluorophores. Dual labeled sequences of highest purity (Table S1, ESI†) were used to exclude the signals from the donor only sample and, further, the donor shot noise contributions were found to be negligible (Table S5, ESI†).^34^ We observed that the donor–acceptor fluorescence intensities of the dual labeled *BCL-2*-C and *c-MYC*-C i-motif sequences produced anti-correlated fluctuations (Fig. 3 and S8, ESI†). The FRET histograms obtained from the time traces were fitted with bi- and single Gaussian distributions. The FRET histogram of the *BCL-2*-C i-motif at pH 4.8 showed a narrow distribution with a mean *ε*_FRET_ ∼ 0.95 (Fig. 3). Using eqn (S5),† the distance (*R*_DA_) between the donor and acceptor dyes of the *BCL-2*-C i-motif was determined to be ∼33.7 Å (Table S6, ESI†), thereby indicating the existence of a compact structure. The single narrow distribution of the *BCL-2*-C i-motif was preserved even after the addition of **PBP1** and **PBP2** (1 equiv.) (Fig. S9, ESI†). The pre-folded *BCL-2*-C i-motif at pH 6 also exhibited a high *ε*_FRET_ value (∼0.88) with a correspondingly low *R*_DA_ ∼ 39 Å, suggesting the presence of folded i-motif structures (Fig. S10, ESI†). The FRET histogram of the free *BCL-2*-C i-motif sequence at pH 7 showed two population distributions, a wide distribution with FRET efficiency (*ε*_FRET_) centered at ∼0.64 (91%) and a narrow distribution centered at *ε*_FRET_ ∼ 0.45 (Fig. 3). The distribution with *ε*_FRET_ ∼ 0.45 was ignored due to the contribution of shot noise (Table S5, ESI†). The lower *ε*_FRET_ ∼ 0.64 value with a large *R*_DA_ (∼50 Å) suggests that the free *BCL-2*-C sequence remains in the unstructured form at pH 7. Upon addition of **PBP1** (1 equiv.), the histogram of the free *BCL-2*-C sequence (pH 7) was shifted to a higher value (*ε*_FRET_ ∼ 0.9) with a *R*_DA_ of ∼36.6 Å, which suggests that **PBP1** induces folding of free C-rich sequences into i-motif structures at pH 7. However, **PBP2** (1 equiv.) induces only a partial shift in the population distributions of the free i-motif sequence at pH 7, exhibiting two major populations with *ε*_FRET_ values of ∼0.67 (*R*_DA_ ∼ 49 Å) and ∼0.95 (*R*_DA_ ∼ 33.7 Å). The mutant *BCL-2*-C C-rich sequence exists in an unstructured form, showing low FRET efficiencies (∼57%) in both Milli-Q water (pH 7) and in 10 mM Na-cacodylate buffer (pH 4.8) (*R*_DA_ ∼ 53 Å) (Fig. 3).

The FRET histogram of the free *c-MYC*-C i-motif sequence at pH 7 showed two major population distributions having mean *ε*_FRET_ values of ∼0.55 and ∼0.8 with *R*_DA_ ∼ 53.2 Å and ∼43.7 Å, respectively (Fig. S8, ESI†). Upon addition of **PBP1** (1 equiv.), the *ε*_FRET_ distribution was shifted towards higher value (∼0.93) (*R*_DA_ ∼ 35.7 Å), indicating the formation of a compact i-motif structure; whereas **PBP2** (1 equiv.) did not significantly alter the FRET distribution pattern of the free *c-MYC*-C i-motif sequence at pH 7. Similar the *BCL-2*-C i-motif, the folded *c-MYC*-C i-motif at pH 4.8 showed a single population with a mean *ε*_FRET_ value ∼0.95, indicating the formation of a more compact structure with lower end-to-end distances (*R*_DA_ ∼ 33.7 Å).

Similar to the folded i-motifs, the folded G-quadruplexes are known to exhibit lower *R*_DA_ values compared to unstructured G-rich sequences.^34^ The free *BCL-2*-G G-quadruplex sequence showed a wide distribution centered at *ε*_FRET_ ∼ 0.6 with a corresponding *R*_DA_ of ∼51.4 Å (Fig. S11a and b, ESI†). **PBP2** (1 equiv.) could significantly shift the populations of free *BCL-2*-G sequence towards higher values (*ε*_FRET_ ∼ 0.95) with a low *R*_DA_ value of ∼33.7 Å, indicating **PBP2** could induce a compact G-quadruplex structure similar to the K^+^-folded G-quadruplex.^34^ However, the free *BCL-2*-G sequence showed two major populations with *ε*_FRET_ values of ∼0.6 (*R*_DA_ ∼ 51.4 Å) and ∼0.85 (*R*_DA_ ∼ 41.2 Å) in the presence of **PBP1** (1 equiv.). The addition of **PBP2** decreased the *R*_DA_ value of free *c-MYC*-G from ∼57 Å to ∼41 Å in the absence of K^+^ ions (Fig. S11c, d and Table S6, ESI†). However, the non-G-quadruplex forming mutated *BCL-2*-G sequence did not exhibit any notable change in *ε*_FRET_ values upon the addition of ligands **PBP1** and **PBP2** (*ε*_FRET_ ∼ 0.6), suggesting that the mutated sequences are unstructured even in the presence of the ligands (Fig. S12, ESI†).

Collectively, the smFRET results suggest that ligand **PBP1** can completely shift the dynamic equilibrium of C-rich *BCL-2*-C and *c-MYC*-C sequences towards the folded i-motifs from the unstructured form under physiologically relevant neutral pH conditions. However, **PBP2** induces only a partial shift in the population distributions of free i-motif sequences at neutral pH but it has the ability to trigger the formation of G-quadruplexes from the unstructured G-rich sequences in the absence of K^+^ ions.

#### (b) Fluorescence lifetime analysis

The differential folding behaviour of free *c-MYC*-C and *BCL-2*-C i-motif sequences upon binding to **PBP1** and **PBP2** was further investigated by measuring the donor decay of dual labeled sequences (Fig. S13–S16, ESI†). The folding states of the dual labeled sequences were assigned on the basis of the *R*_DA_ determined from the average lifetime (*τ*_avg_) of donor (D) labeled *c-MYC*-C and *BCL-2*-C (*τ*_D_) and donor–acceptor (DA) dual labeled *c-MYC*-C and *BCL-2*-C (*τ*_DA_) i-motif sequences using eqn (S10) (Tables 2 and S7, ESI†). The free *BCL-2*-C i-motif sequence at pH 7 exhibited a *R*_DA_ value of ∼52 Å, which decreased to ∼43 Å for the folded *BCL-2*-C (pH 4.8). A similar decrease in *R*_DA_ value was observed for the free *c-MYC*-C i-motif sequence upon decreasing the pH from 7 (*R*_DA_ ∼ 54.3 Å) to 4.8 (*R*_DA_ ∼ 44 Å). As observed from smFRET, the *R*_DA_ values of the free *BCL-2*-C and *c-MYC*-C i-motif sequences decreased to ∼40 Å and ∼47 Å, respectively, upon binding to **PBP1**, at pH 7 (Table 2). However, no sharp decrease in *R*_DA_ values was observed after the addition of **PBP2** to *BCL-2*-C and *c-MYC*-C i-motif sequences at pH 7.

**Table 2:** Lifetime parameters of *BCL-2*-C and *c-MYC*-C i-motifs

System		*τ*_avg_ ^*a*^	*ε*_FRET_ ^*a*^	*R*_DA_ ^*a*^ (Å)
*BCL-2*-C (pH 7)	D	3.78	0.59	52
	DA	1.56		
*BCL-2*-C-mut (pH 7)^*b*^	D	3.62	0.53	54
	DA	1.7		
*BCL-2*-C (pH 4.8)	D	4.32	0.81	43.2
	DA	0.84		
*BCL-2*-C + **PBP1** (pH 7)	D	3.24	0.87	40.1
	DA	0.42		
*BCL-2*-C-mut + **PBP1** (pH 7)	D	2.05	0.55	53
	DA	0.92		
*BCL-2*-C + **PBP2** (pH 7)	D	2.43	0.67	48.9
	DA	0.79		
*BCL-2*-C-mut + **PBP2** (pH 7)	D	2.34	0.59	52
	DA	0.96		
*c-MYC*-C (pH 7)	D	3.32	0.52	54.3
	DA	1.6		
*c-MYC*-C (pH 4.8)	D	4.62	0.79	44.1
	DA	0.97		
*c-MYC*-C + **PBP1** (pH 7)	D	3.04	0.72	47
	DA	0.87		
*c-MYC*-C + **PBP2** (pH 7)	D	2.7	0.62	50.7
	DA	1.02		

^*a*^±10%.

^*b*^ *BCL-2*-G-mut: 5′-FAM-d(AG_2_TGCG_2_TCGC G_2_AAG_2_A_2_G_2_ TG_2_ C GTAA GCG_2_TGCTG)-TAMRA-3′.

Conversely, the addition of **PBP2** decreased the *R*_DA_ value of free *BCL-2*-G and *c-MYC*-G from ∼55 Å to ∼41 Å in the absence of K^+^ ions (Table S8, ESI†), which indicates that **PBP2** folds single stranded *BCL-2*-G and *c-MYC*-G G-rich sequences into G-quadruplex structures. However, no significant changes in the *R*_DA_ values of mutant *BCL-2* C-rich and G-rich sequences were noted upon addition of **PBP1** and **PBP2** (Tables 2, S8 and Fig. S15, ESI†). These results suggest that the observed changes in *R*_DA_ values of the investigated sequences are due to the formation of folded G-quadruplex or i-motif structures in the presence of ligands.

In agreement with the smFRET and lifetime analyses, the CD spectroscopy also supports the idea that the ligand **PBP1** triggers the formation of *BCL-2*-C and *c-MYC*-C i-motif structures and **PBP2** induces the formation of G-quadruplex structures (Fig. S17–S24, ESI†). Moreover, the change in CD intensity with the mole fraction of ligands (Job’s plot) suggests a 1 : 1 binding stoichiometry of **PBP1** and **PBP2** with i-motifs and G-quadruplexes, respectively (Fig. S25 and S26, ESI†).

### Growth inhibition assay

The growth-inhibitory activity of ligands **PBP1** and **PBP2** on human breast adenocarcinoma (MCF-7) cells, human colon cancer (HCT116) cells, and normal mouse myoblast (C2C12) cells were evaluated using MTT assay (Fig. S27 and S28, ESI†).^52^ After 24 h treatment of cells, **PBP1** showed IC_50_ values of 17.9 ± 1.8 μM and 18.5 ± 1.9 μM in MCF-7 and HCT116 cells, respectively. Ligand **PBP2** displayed IC_50_ values of 3.3 ± 0.7 μM and 3.9 ± 0.9 μM in MCF-7 cells and HCT116 cells, respectively, after 24 h (Table S9, ESI†). The IC_50_ values suggested a differential effect of **PBP1** and **PBP2** on cancer cells after a 48 h treatment. When the cells were treated with **PBP1** for 48 h, no significant change in IC_50_ values (14.4 ± 1.4 μM for MCF-7 and 15.1 ± 1.5 μM for HCT116 cells) was observed (Fig. S28 and Table S10, ESI†). However, treatment of cells with **PBP2** for 48 h caused a nearly 10 fold decrease in IC_50_ values (1.7 ± 0.2 μM for MCF-7 and 1.3 ± 0.15 μM for HCT116 cells) as compared to **PBP1**. This indicates that **PBP2** can considerably inhibit the growth of cancer cells after a 48 h treatment, while **PBP1** shows less potent cytotoxic activity. Importantly, both **PBP1** and **PBP2** exhibited negligible toxicity towards normal C2C12 cells after a 48 h treatment, even at >40 μM concentration.

### Ligand-dependent *BCL-2* expression in cancer cells

#### (a) qRT-PCR analysis

**Fig. 4:**
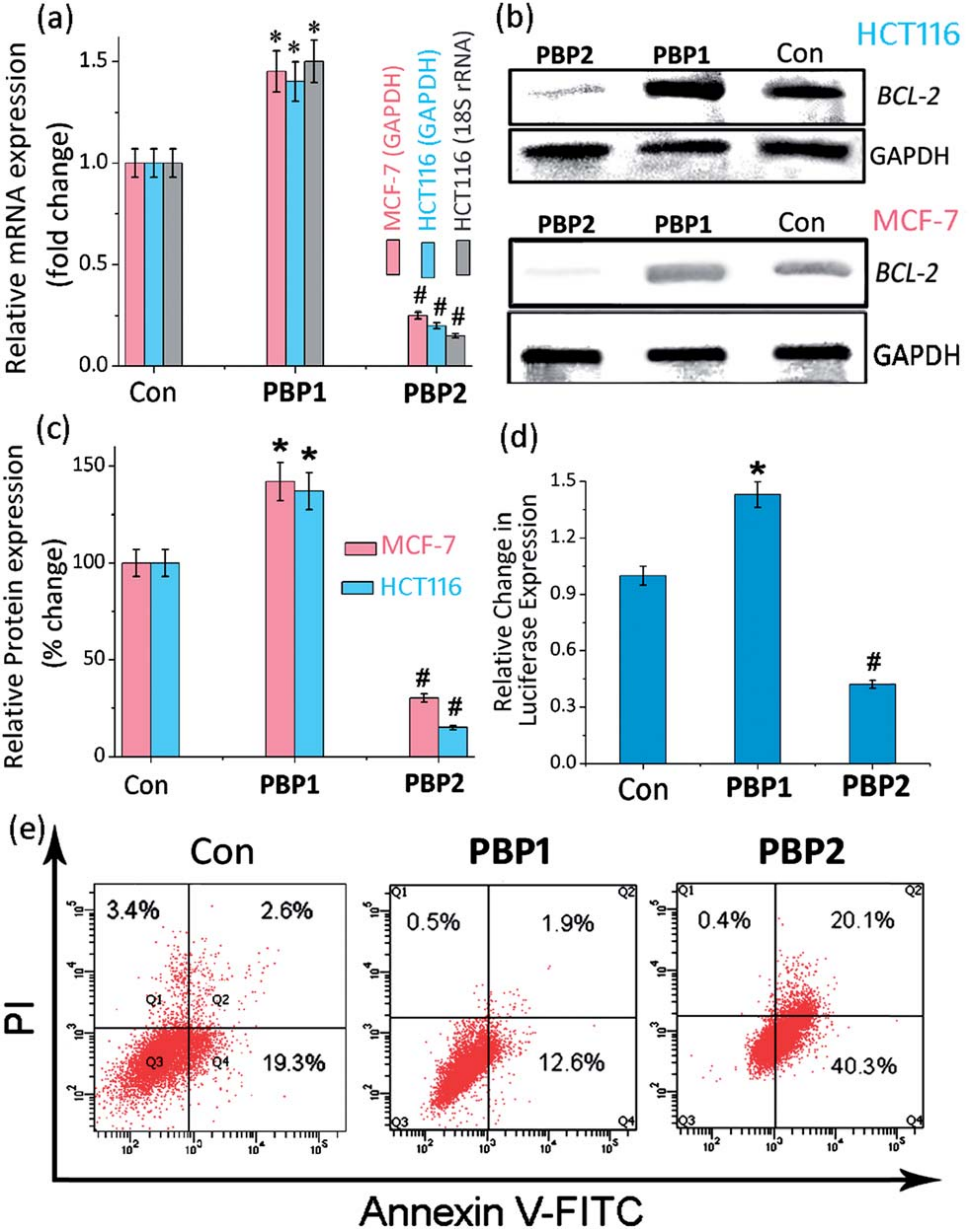
(a) Determination of the transcriptional regulation of *BCL-2* mRNA in the presence of IC_50_ doses (24 h) of **PBP1** or **PBP2** in cancer cells (MCF-7 and HCT116) *via* qRT-PCR and quantified by double delta *C*_*t*_ analysis using GAPDH and 18S rRNA as reference genes. Data is presented in terms of fold change (the expression of control is 1 fold). The data are shown as mean ± SD. *P < 0.05, ^#^ *P* < 0.01, *versus* untreated cancer cells. (b) Immunoreactive bands of the *BCL-2* protein were analyzed *via* western blot in MCF-7 and HCT116 cells. The data are shown as mean ± SD. *P < 0.05, ^#^ *P* < 0.01, *versus* untreated cancer cells. (c) The protein expression of the *BCL-2* protein in the presence of IC_50_ doses (24 h) of **PBP1** or **PBP2** in MCF-7 and HCT116 cancer cells. (d) The relative luciferase expression in the LB322 *BCL-2* promoter containing firefly plasmid normalized with pRL-TK *Renilla* plasmid (FF/RL) upon treatment with 5 μM of **PBP1** and **PBP2** in HCT116 cells, data shown here as mean ± SD. *P < 0.05, ^#^ *P* < 0.01, *versus* untreated cancer cells. (e) Flow cytometric analysis upon treatment with 5 μM of **PBP1** and **PBP2** in serum starved MCF-7 cells, Q3, Q4, Q2, and Q1 indicate healthy cells, early, late apoptotic, and necrotic cells, respectively.

To investigate the ability of **PBP1** and **PBP2** to regulate the expression of the *BCL-2* gene in biological systems, we measured the level of *BCL-2* expression at transcriptional and translational levels. After a 24 h treatment with IC_50_ dose (24 h) of **PBP1** and **PBP2**, the total RNA was isolated from MCF-7 and HCT116 cells. The level of transcription of *BCL-2* was quantified using qRT-PCR. Gene expression was normalized against the expression of the constitutively expressed house-keeping gene, glyceraldehyde-3-phosphate dehydrogenase (GAPDH). Treatment with the **PBP2** reduced *BCL-2* mRNA level to 0.3-fold (by 70%) and 0.24-fold (by 76%) in MCF-7 and HCT116 cells, respectively, compared to the control (Fig. 4a and Table S14, ESI†). In contrast, when cells were treated with **PBP1**, the *BCL-2* mRNA expression was upregulated by 1.45-fold (45%) and 1.35-fold (35%) in MCF-7 and HCT116 cells, respectively, compared to the control (Fig. 4a and Table S12, ESI†). In addition to GAPDH, gene expression was also normalized using 18S rRNA as a control gene (Fig. S29, ESI†). Treatment with an IC_50_ dose (24 h) of **PBP1** upregulated the *BCL-2* mRNA level by 1.5-fold (50%), whereas treatment with IC_50_ dose (24 h) of **PBP2** reduced *BCL-2* mRNA level to 0.13-fold (87%) with respect to the 18S rRNA control in HCT116 cells (Tables S11 and S13, ESI†). However, GAPDH mRNA and 18S rRNA were equally expressed in the untreated control and ligand treated MCF-7 and HCT116 cells, indicating the gene specific behaviour of the bis-prolinamide derivatives.

#### (b) Western blot analysis

Having assessed the expression of *BCL-2* at the transcriptional level, we employed western blot analysis to observe the effect of these ligands at the translational level (Fig. 4b, c and S30, ESI†). Protein levels of *BCL-2* and GAPDH were measured in MCF-7 and HCT116 cells treated with **PBP1** and **PBP2** for 24 h at their respective IC_50_ doses (24 h). The western blots exhibited the differential effect of **PBP1** and **PBP2** on the expression of *BCL-2* compared to the control cells, which is in good agreement with the qRT-PCR analysis data. The protein expressions calculated from densitometric analysis of western blots were normalized for ligand treated cells against untreated control cells. In **PBP2** treated MCF-7 and HCT116 cells, the *BCL-2* protein expression was downregulated by 70% and 85%, respectively (Fig. 4c). In contrast, the *BCL-2* protein was upregulated by 40% and 50% in **PBP1** treated MCF-7 and HCT116 cells, respectively. On the other hand, negligible reduction in GAPDH expression was observed in both treated and control cells. These results suggest that the *meta*-prolinamide **PBP2** can downregulate *BCL-2* expression, whereas the treatment with *para*-prolinamide **PBP1** results in upregulation of the *BCL-2* expression at both the mRNA and protein levels in cancer cells.

#### (c) Dual-luciferase assay

**Scheme 2:**
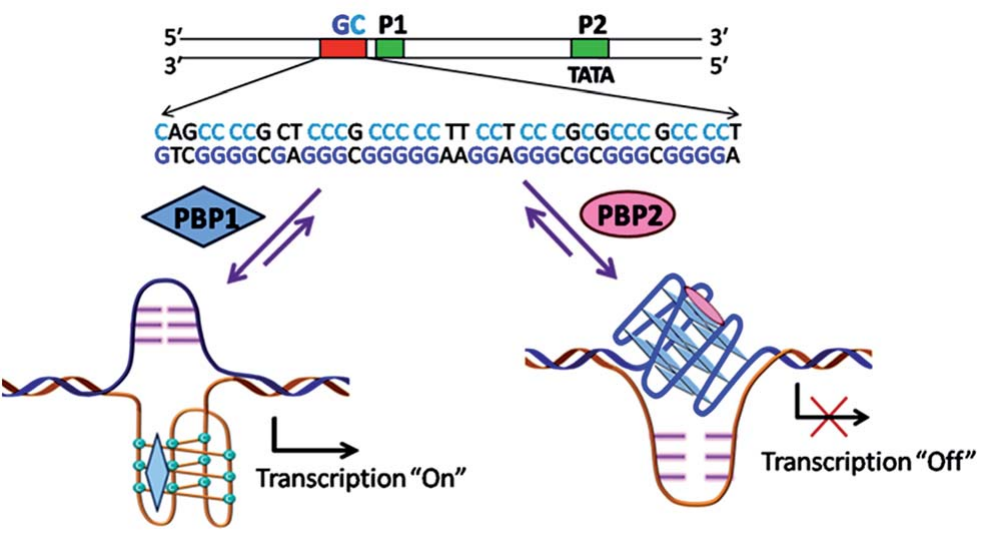
A schematic representation of the working hypothesis: The *BCL-2* GC-rich promoter region forming G-quadruplexes and i-motifs in opposite strands in the presence of the peptidomimetic ligands **PBP1** and **PBP2**.

In order to investigate the influence of the ligands (**PBP1** and **PBP2**) on the *BCL-2* gene expression, we employed a dual-luciferase reporter assay (Scheme 2 and Fig. 4d). Reporter vectors containing wild-type *BCL-2* promoter sequences (i-motif and G-quadruplex forming sequences) in the upstream region of the firefly luciferase coding gene (LB322) were co-transfected with the *Renilla* luciferase vector containing a non G- or C-rich promoter sequence (pRL-TK) into HCT116 cells. After cellular uptake of the reporter luciferase vectors, 5 μM of **PBP1** or **PBP2** was added to the cells. As expected, the *Renilla* luciferase expression was unaffected by the ligands due to the absence of C-rich or G-rich sequences. Hence, the expression of *BCL-2* firefly luciferase was normalized relative to the *Renilla* luciferase expression.

Upon treatment with **PBP2**, the *BCL-2* promoter-linked luciferase expression was decreased by 58% relative to the untreated control. In contrast, treatment with **PBP1** exhibited a 42% increase in *BCL-2* promoter-linked luciferase expression compared to the control. To further validate our results, we also investigated the effect of **PBP1** and **PBP2** on a firefly luciferase vector (pBV-Luc) containing non i-motif or G-quadruplex sequence (Fig. S31, ESI†). Interestingly, we did not observe any notable change in firefly luciferase expression in pBV-Luc treated HCT116 cells upon treatment of **PBP1** and **PBP2** compared to untreated control. In addition, ligand **PBP1** did not show any significant change in the expression of reporter vector containing other promoter i-motif or G-quadruplex forming sequences such as *c-MYC* (Del 4 plasmid, Fig. S32, ESI†). These results indicate that **PBP1** and **PBP2** may regulate *BCL-2* expression by targeting *BCL-2* promoter i-motifs or quadruplexes in cancer cells.

### Detection of apoptosis using Annexin V and caspases 3/7

To further investigate the influence of bis-prolinamides on cell survival, flow cytometry was employed using Annexin V and PI dual staining assays (Fig. 4e and S33, ESI†). Since **PBP1** did not influence the healthy cancer cells in an apoptosis assay (unpublished data), we prepared model apoptotic cells *via* 48 h serum starvation in order to investigate the anti-apoptotic properties.^53^ MCF-7 and HCT116 cells were treated with 5 μM **PBP1** and **PBP2** after serum starvation. Control MCF-7 cells show a significant percentage of apoptotic cells (∼22%) due to serum starvation. Interestingly, treatment with **PBP1** reduced the percentage of apoptotic MCF-7 cells to ∼14% whereas **PBP2** efficiently increased the percentage of apoptotic MCF-7 cells to ∼60%. Similar results were obtained for **PBP1** and **PBP2** treated HCT116 cells (Fig. S33, ESI†).

Since activation of caspases is an important process during apoptosis, the quantitative detection of executioner caspases 3 and 7 in HCT116 cells upon treatment with peptidomimetic ligands **PBP1** and **PBP2** was investigated using FLICA reagent based flow cytometry assay (Fig. S34 and Table S15, ESI†).^54^ The serum starved control cells exhibited moderate levels of active caspases 3 and 7 (∼18.4%). At 24 h post treatment with ligand **PBP1** (5 μM), the level of active caspases 3/7 was significantly decreased (∼5.1%). However, cells incubated with ligand **PBP2** (5 μM) for 24 h exhibited a higher level of active caspases 3/7 (∼44.7%). These results suggest that **PBP1** decreases the level of active caspases 3 and 7 in cancer cells whereas **PBP2** induced apoptosis is associated with the activation of caspases 3 and 7. However, the exact molecular mechanism of this behaviour is under investigation.

## Conclusions

We have demonstrated that two flexible peptidomimetic congeners, **PBP1** and **PBP2**, synthesized using ‘click chemistry’, can exhibit distinguishable recognition between i-motifs and G-quadruplexes. FRET melting and fluorescence spectroscopic studies reveal that both ligands show high selectivity for i-motifs and G-quadruplexes over duplex DNA. These studies also indicate that **PBP1** preferentially binds to the *BCL-2*-C i-motif over G-quadruplexes and **PBP2** selectively binds to G-quadruplexes over i-motifs. In addition, smFRET studies indicate that **PBP1** folds the unstructured *BCL-2* and *c-MYC* C-rich DNA sequences into i-motif structures at neutral pH; whereas **PBP2** promotes G-quadruplex formation from single stranded *BCL-2* and *c-MYC* G-rich sequences in the absence of metal ions. Cellular studies revealed that **PBP1** upregulates *BCL-2* gene expression while **PBP2** inhibits *BCL-2* gene expression. Furthermore, **PBP2** triggers apoptosis *via* activation of caspases 3 and 7; whereas **PBP1** reduces the level of active caspases 3/7 and decreases the percentage of apoptotic cancer cells. These results indicate that a small change in the ligand structure can have a dramatic effect on the molecular recognition properties, providing a new platform to achieve differential recognition of G-quadruplexes and i-motifs. These observations further suggest that ligand induced folding of i-motifs or G-quadruplexes may provide an attractive way to control gene expression and to develop therapies for cancer and neurodegenerative diseases.

## Conflicts of interest

The authors declare no conflict of interest.
