# Supplementary material for: Preferential targeting of i-motifs and G-quadruplexes by small molecules
Source: Chem Sci. 2017 Sep 8;8(11):7448–56. doi: 10.1039/c7sc02693e (PMC5674183; doi:10.1039/c7sc02693e)
Supplement: Supplementary file 1 [file SC-008-C7SC02693E-s001.pdf]

Electronic Supplementary Material (ESI) for Chemical Science.
This journal is © The Royal Society of Chemistry 2017

SUPPORTING INFORMATION

Preferential targeting of I-motifs and G-quadruplexes by small molecules

Manish Debnath,[1] Shirsendu Ghosh,[2] Ajay Chauhan,[1] Rakesh Paul,[1] Kankan Bhattacharyya,[2] Jyotirmayee Dash[1,*]

[a]Department of Organic Chemistry, Indian Association for the Cultivation of Science, Jadavpur, Kolkata-700032, India; email: ocjd@iacs.res.in, [b]Department of Physical Chemistry, Indian Association for the Cultivation of Science, Jadavpur, Kolkata-700032, India

Table of contents

1.0	General information	S2
2.0	Energy minimized structures of peptidomimetic ligands	S3
3.0	Synthesis of compounds	S4
4.0	NMR spectra of compounds	S11
5.0	HPLC chromatogram	S18
6.0	Melting analysis by FRET	S19
7.0	TO displacement assay	S22
8.0	Fluorescence binding experiments	S24
9.0	Confocal microscopy	S27
10.0	Donor shot noise data	S29
11.0	smFRET analysis	S31
12.0	Lifetime data	S35
13.0	CD spectroscopy	S40
14.0	MTT assay	S48
15.0	qRT-PCR	S50
16.0	Western Blot	S53
17.0	Dual luciferase reporter assay	S54
18.0	Flow cytometric assay of apoptosis	S56
19.0	Molecular Modeling	S57
20.0	Caspase 3/7 assay	S57

1.0 General information:

The solvents and reagents used in this study were purified by standard techniques reported in Armarego, W. L. F., Chai, C. L. L., Purification of Laboratory Chemicals, 5th edition, Elsevier, 2003; or used as supplied from commercial sources (Sigma-Aldrich Corporation® unless stated otherwise). The reactions were generally carried out under inert atmosphere unless otherwise noted. TLC was performed on Merck Kieselgel 60 F254 plates, and spots were visualized under UV light. The products were purified by flash chromatography on silica gel (100-200 mesh, Merck). ^{1}H and ^{13}C NMR spectra were recorded on either Brüker ADVANCE 500 (500 MHz and 125 MHz), or JEOL 400 (400 MHz and 100 MHz) instruments using deuterated solvents as detailed and at ambient probe temperature (300 K). Chemical shifts are reported in parts per million (ppm) and referred to the residual solvent peak. The following notations are used: singlet (s); doublet (d); triplet (t); quartet (q); multiplet (m); broad (br). Coupling constants are quoted in Hertz and are denoted as *J*. Mass spectra were recorded on a Micromass® Q-Tof (ESI) spectrometer. All compounds were analyzed for purity by HPLC. The compounds showed ≥ 95% purity. All general chemicals and labeled DNA sequences required for biophysical analysis were purchased from Sigma-Aldrich. The labeled DNA sequences of highest purity were purchased for best results. The typical cell culture reagents and the antibodies were purchased from Thermo Fisher Scientific and Merck Millipore, unless stated otherwise. The Annexin V-FITC kit for apoptosis assay was purchased from Life technologies. The *BCL-2* luciferase plasmid LB322 (*BCL-2* from ATG to -3934) was a gift from Linda Boxer (Addgene plasmid # 15381). The renilla luciferase plasmid pRL-TK was a gift from Dr. Susanta Roychoudhury, Indian Institute of Chemical Biology, Kolkata. The dual luciferase reporter assay kit was purchased from Promega. The Vybrant FAM Caspase 3/7 assay kit was purchased from Invitrogen.

2.0 Energy minimized structures of peptidomimetic ligands

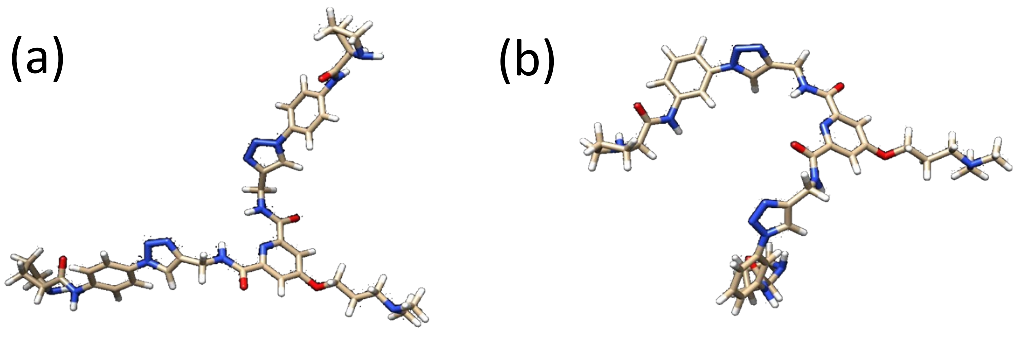

Fig. S1 Energy minimized 3D structure of **PBP1** (a) and 3D structure of **PBP2** (b).

3.0 Synthesis of compounds:

The azido prolinamides **1** and **2** with the azide group at the *para* and *meta* position were prepared by amide coupling of N-Bocproline **4** with the *para* and *meta* azido anilines **5** and **6** using DCC, HOBT in dichloromethane (Scheme S1). The N-Bocproline **4** was prepared from L-proline **S1** in 95 % yield.[1] The dialkyne building block **S2** was prepared from cheledamic acid **7**. Cheledamic acid **7** was treated with oxalyl chloride to generate the corresponding acid chloride which was subsequently coupled with propargyl amine **8** to give the dialkyne **S2** in 65 % yield. The dialkyne **S2** was then treated with Cs_2CO_3 and 3-dimethylaminopropyl chloride **9** in dry DMF at 70 °C to obtain the dialkyne **3** in 95 % yield. The azido prolinamide derivatives **1** and **2** were then treated with the dialkyne **3** in the presence of $CuSO_4·5H_2O$ and Na-ascorbate in $^tBuOH/H_2O$ (1:1) as solvent to yield the pyridyl bis-prolinamide derivatives **S3** and **S4** respectively in high yields. Subsequent removal of the Boc group afforded the bis-prolinamide peptidomimetic ligands **PBP1** and **PBP2** in near quantitative yields.

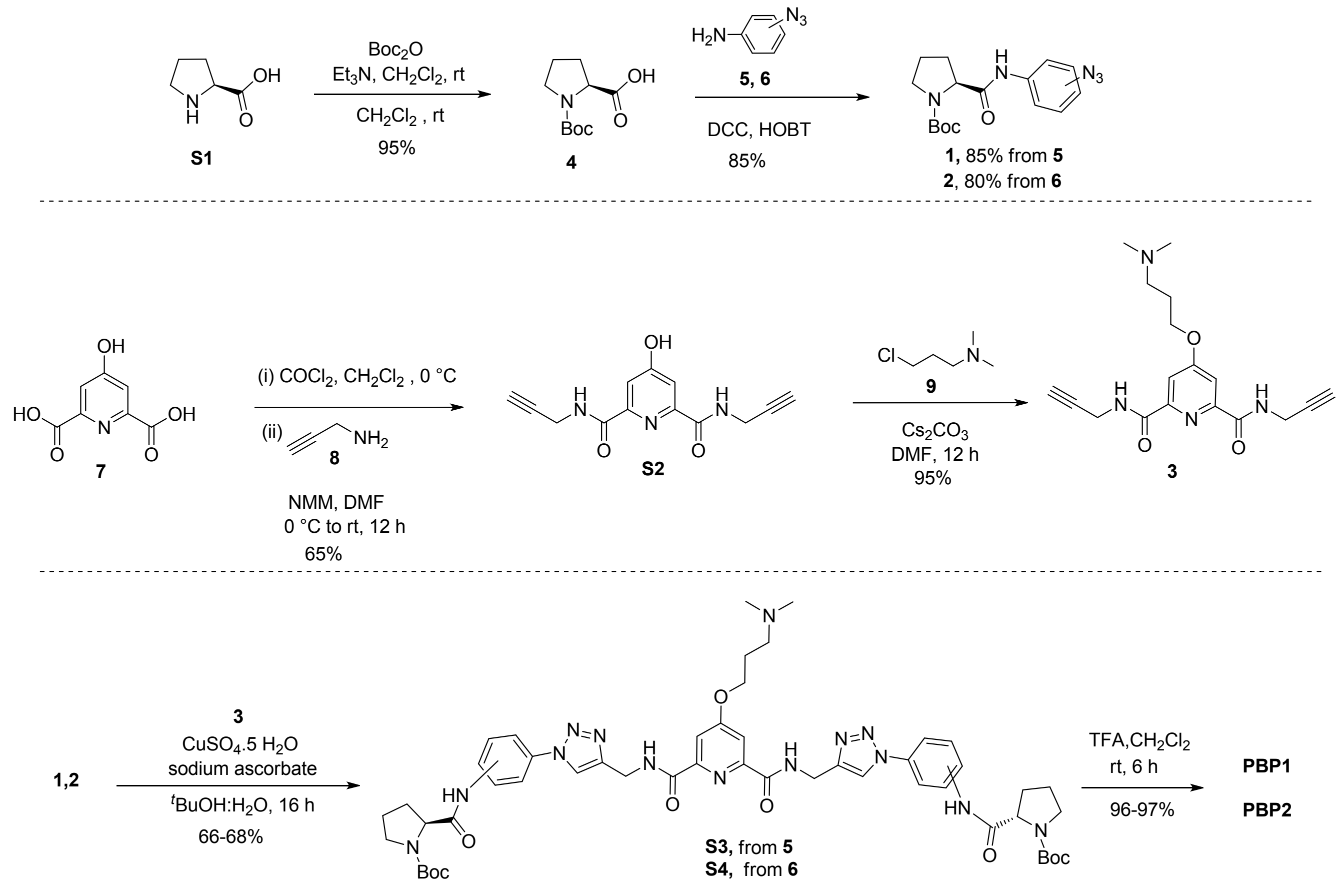

Scheme S1. Synthesis of bis-prolinamide derivatives (**PBP1** and **PBP2**).

Preparation of *N*-Bocproline 4:[1]

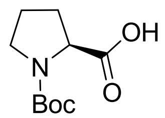

Triethylamine (1.6 mL, 8.7 mmol, 2.0 equiv.) was added to a solution of L-Proline S**1** (500.0 mg, 4.4 mmol, 1.0 equiv.) in dry dichloromethane (3.0 mL/1 mmol) under an argon atmosphere. The reaction mixture was stirred for 15 min at room temperature. Di-*tert*-butyl dicarbonate (1.2 mL, 5.2 mmol, 1.2 equiv) was added dropwise at 0 °C and the reaction mixture was stirred for 12 h at room temperature. Reaction was quenched with dropwise addition of saturated $KHSO_4$ solution up to pH 3, extracted with dichloromethane (3 x 20 mL), washed the organic layer with brine, concentrated under vacuum, recrystallized from hexane obtained as a white solid product **4** (0.9 g, 95 %). ^{1}H NMR (500 MHz, DMSO-d_6): 12.50 ($s_{br,}$ 1H), 4.05-4..03 (m, 1H), 2.39-2.10 (m, 2H), 1.84-1.38 (m, 4H), 1.39 (s, 9H). ^{13}C NMR (125 MHz): 176.0, 154.5, 80.8, 65.8, 50.3, 28.4, 27.8, 24.5. HRMS (ESI) calculated for $C_{10}H_{18}NO_4$ $(M+H)^+$: 216.1281; found, 216.1528.

Preparation of *para* azido prolinamide 1:

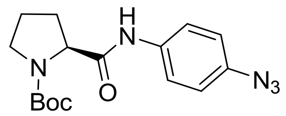

To an ice-cold suspension of *N*-Bocproline **4** (1.0 g, 4.65 mmol) in dry CH_2Cl_2 (25 mL), DCC (1.06 g, 5.1 mmol, 1.1 equiv.) and HOBT (691 mg, 5.1 mmol, 1.1 equiv.) were added and the mixture was allowed to stir for 45 min. Then 4-azidoaniline **5** (624 mg, 4.65 mmol, 1.0 equiv.) in 20 mL dry CH_2Cl_2 was added drop-wise to the reaction mixture, and stirred for 12 h. After complete consumption of all the azides (TLC monitoring), the reaction mixture was filtered through celite, washed with ethyl acetate (50 mL) and concentrated under vacuum. The product was purified by flash chromatography using hexane-ethylacetate (95:5 to 85:15) as eluent to afford the desired product **1** as a yellow solid (1.57 g, 95 %); m.p. = 136-138 °C; ^{1}H NMR (400 MHz, DMSO-d_6): 9.84 (s, 1H), 7.54 (d, 2H, J = 9.1 Hz), 6.96 (d, 2H, J = 8.5 Hz), 4.46 (t, 1H, J = 3.7 Hz), 3.45-3.38 (m, 2H), 2.44 (s_{br}, 1H), 1.99-1.90 (m, 3H), 1.48 (s, 9H); ^{13}C NMR (100 MHz): 170.0, 156.5, 135.5, 134.9, 120.8, 119.2, 80.9, 60.4, 47.3, 28.3, 27.5, 24.5; FT-IR (KBr): 3268, 3123, 3044, 2974, 2935, 2888, 2417, 2258, 2111, 1740, 1740, 1690, 1604, 1534, 1413, 1250, 1163, 986, 918, 830, 777, 743; HRMS (ESI) calculated for $C_{16}H_{21}N_5O_3K$ $(M+K)^+$: 370.1281; found, 370.1268.

Preparation of *meta* azido prolinamide 2:

[1] S. Paladhi, J. Das, P. K. Mishra and J. Dash, *Adv. Synth. Catal.* 2013, **355**, 274-280.

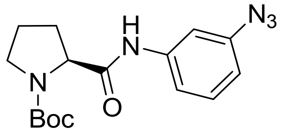

To an ice-cold suspension of *N*-Bocproline **4** (1.1 g, 5.1 mmol) in dry CH_2Cl_2 (25 mL), DCC (1.17 g, 5.6 mmol, 1.1 equiv.) and HOBT (760 mg, 5.6 mmol, 1.1 equiv.) were introduced under argon atmosphere and allowed to stir for 45 min. Then a solution of 3-azidoaniline **6** (687 mg, 5.1 mmol, 1.0 equiv.) in dry CH_2Cl_2 (25 mL) was added dropwise to the reaction mixture, and the reaction mixture was allowed to stir for 12 h following TLC. Upon completion, the reaction mixture was filtered through celite, washed with ethyl acetate (50 mL) and concentrated under reduced pressure. The product was then purified by flash chromatography using hexane-ethyl acetate (95:5 to 85:15) as eluent to afford the desired product **2** as a yellow solid (1.53 g, 91 %). m.p. = 131-133 °C; ^{1}H NMR (400 MHz, DMSO-d_6): 10.04 (s, 1H), 7.65 (s, 1H), 7.39 (s, 1H), 7.17 (s, 1H), 7.08 (s, 1H), 6.75 (s, 1H), 4.16 (t, 1H, *J* = 3.7 Hz), 3.45-3.40 (m, 2H), 2.44-2.10 (m, 1H), 1.89-1.76 (m, 3H), 1.39 (s, 9H); ^{13}C-NMR (100 MHz, $CDCl_3$): 170.6, 156.0, 140.2, 139.9, 129.7, 115.7, 114.0, 109.8, 80.8, 60.4, 47.2, 28.3, 24.5; FT-IR (KBr): 3341, 3221, 3095, 2941, 2835, 2801, 2561, 2158, 2151, 1751, 1738, 1685, 1631, 1556, 1447, 1221, 1101, 918, 826, 741; HRMS (ESI) calculated for $C_{16}H_{22}N_5O_3$ $(M+H)^+$: 332.1717; found, 332.1721.

Synthesis of S2:

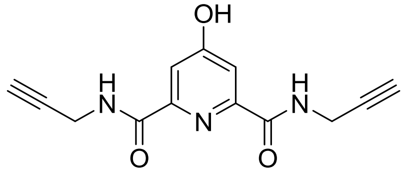

Oxalyl chloride (10.0 mL) was added dropwise to dry DCM containing 4-hydroxy pyridine-2,6-dicarboxylic acid **7** (200.0 mg, 1.09 mmol, 1.0 equiv.) at 0 °C and allowed to stir at 80 °C for 3 h. The reaction mixture was concentrated and dried under vacuum to provide the crude 4-hydroxy pyridine-2, 6-dicarboxylic acid chloride. The crude product was dissolved in DMF (10.0 mL) and cooled to 0 °C. NMM (1.1 mL, 10.1 mmol, 8.4 equiv.) and propargylamine **8** (0.245 mL, 3.82 mmol, 3.5 equiv.) were added under an argon atmosphere at 0 °C and allowed to stir at room temperature for 12 h. The solvent was removed in vacuum and the residue was purified by column chromatography using MeOH–CH_2Cl_2 (4:96) to give the compound **S2** (180.6 mg, 64.5 %) as white solid. m.p. 238-240 °C. ^{1}H-NMR (400 MHz, DMSO-d_6): 9.69 (t, 2H, *J* = 6.1 Hz), 7.57 (d, 2H, *J* = 1.5 Hz), 4.19-4.17 (m, 4H), 3.21 (t, 2H, *J* = 2.1 Hz);

^{13}C-NMR (125 MHz, DMSO-d$_6$): 166.8, 163.1, 150.3, 111.8, 81.2, 73.2, 28.1. HRMS (ESI) calculated for $C_{13}H_{11}N_3O_3$ $(M+H)^+$: 258.0808; found, 258.0811.

Synthesis of pyridyl dialkyne 3:

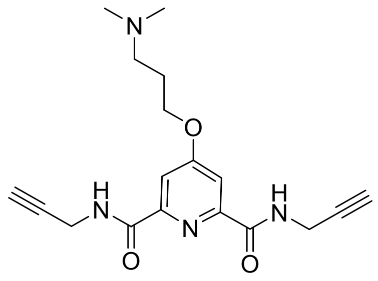

Cs_2CO_3 (3.12 g, 22.75 mmol, 2.5 equiv.) was added to a solution of **S2** (150.0 mg, 0.582 mmol, 1.0 equiv.) in dry DMF (8 mL) and stirred for 30 min at RT under argon atmosphere and 3 dimethyl aminopropylchloride hydrochloride **9** (110.12 mg, 0.69 mmol, 2.2 equiv.) was added and stirred at 70 °C for overnight. The reaction mixture was concentrated to dryness and purified by flash chromatography using methanol:DCM (15:85) to obtain the desired product **3** as a colourless solid (188.3 mg, 94.33 %); m.p. 118-120 °C. ^1H-NMR (500 MHz, CD_3OD): 7.79 (s, 2H), 4.30-4.28 (m, 2H), 4.22 (d, 4H, J = 2.5 Hz), 2.99 (t, 2H, J = 7.8 Hz), 2.64 (s, 6H), 2.63-2.62 (t, 2H, J = 2.5 Hz), 2.20-2.19 (m, 2H); ^{13}C-NMR (125 MHz, CD_3OD): 169.2, 165.5, 152.1, 112.2, 80.6, 72.3, 67.7, 56.7, 44.6, 29.7, 26.6. HRMS (ESI) calculated for $C_{18}H_{22}N_4O_3$ $(M+H)^+$: 343.1716; found, 343.1719.

Synthesis of compound S3:

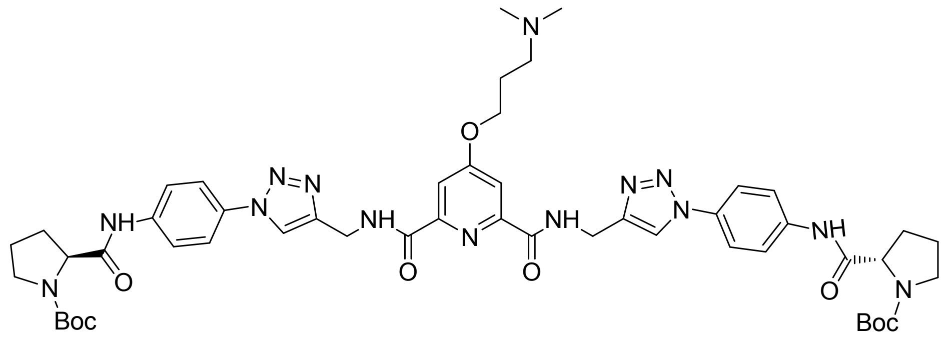

A mixture of pyridyl dialkyne **3** (200.0 mg, 0.584 mmol, 1.0 equiv.), azido prolinamide **1** (425.3 mg, 1.28 mmol, 2.2 equiv.), sodium ascorbate (116.6 mg , 0.467 mmol, 0.8 equiv.) and $CuSO_4.5H_2O$ (34.5 mg, 0.18 mmol, 0.3 equiv.) in $H_2O/^tBuOH$ (7:3, 10 mL) were stirred at room temperature for 16 h. After consumption of the starting material **1** (monitored by TLC), the reaction mixture was concentrated under vacuum and the residue was purified by column chromatography using MeOH-CH_2Cl_2 (20:80) to give

corresponding product **S3** (392.0 mg, 68 %) as pale yellow solid, m.p. 95-97 °C. ^{1}H-NMR (400 MHz, $CDCl_3$): 9.92 (s, 2H), 9.30 (s, 2H), 7.64-7.59 (m, 4H), 7.42 (d, 4H, *J* = 9.3 Hz), 7.15 (d, 4H, *J* = 8.7 Hz), 4.89 (s_{br}, 2H), 4.51 (s, 2H), 4.42-4.39 (m, 2H), 4.16 (s, 4H), 3.59-3.44 (m, 4H), 2.54-2.47 (m, 2H), 1.48 (s, 18H). ^{13}C-NMR (100 MHz, $CDCl_3$): 171.9, 167.6, 163.8, 155.4, 150.5, 145.5, 139.3, 131.7, 120.0, 119.9, 119.4, 110.9, 80.9, 67.2, 60.7, 56.1, 47.4, 45.5, 35.0, 29.9, 28.7, 27.1, 24.8. HRMS (ESI) calculated for $C_{50}H_{64}N_{14}O_9$ $(M+H)^+$: 1005.5051; found, 1005.5055.

Synthesis of compound S4:

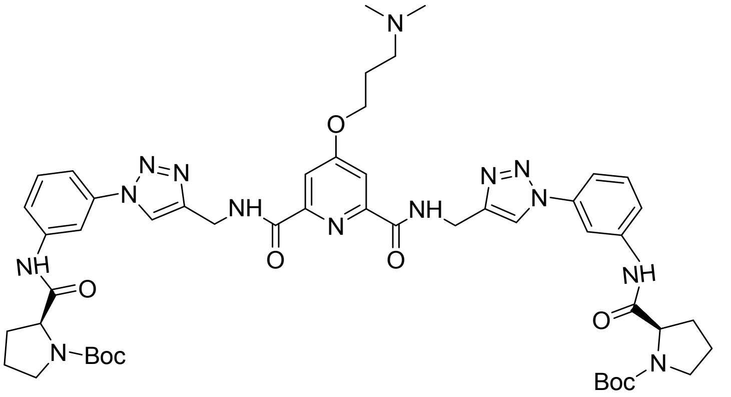

A mixture of pyridyl dialkyne **3** (200.0 mg, 0.584 mmol, 1.0 equiv), azido prolinamide **2** (425.3 mg, 1.28 mmol, 2.2 equiv.), sodium ascorbate (116.6 mg , 0.467 mmol, 0.8 equiv.) and $CuSO_4.5H_2O$ (34.5 mg, 0.18 mmol, 0.3 equiv.) in $H_2O/^tBuOH$ (7:3, 10 mL) were stirred at room temperature for 16 h. After consumption of the starting material **2** (monitored by TLC), the reaction mixture was concentrated under vacuum and the residue was purified by column chromatography using MeOH-CH_2Cl_2 (20:80) to give corresponding product **S4** (385.2 mg, 66 %) as a pale solid. ^{1}H-NMR (500 MHz, $CDCl_3$): 9.86 (s, 2H), 9.32 (s, 2H), 7.67 (s, 2H), 7.59-7.55 (m, 4H), 7.25 (s, 2H), 7.08-6.98 (m, 4H), 4.80 (s, 2H), 4.48 (s, 4H), 4.16 (s, 2H), 3.56-3.43 (m, 4H), 2.99 (s, 2H), 2.63 (s, 6H), 2.20 (s, 2H), 2.19-2.00 (s, 6H), 1.87 (s, 2H), 1.38 (s, 18H); ^{13}C-NMR (125 MHz, $CDCl_3$): 171.7, 166.9, 163.5, 155.3, 150.3, 145.1, 139.6, 136.2, 129.7, 119.3, 118.8, 114.3, 110.7, 109.6, 80.6, 66.2, 60.5, 55.1, 47.3, 43.5, 34.5, 29.9, 28.1, 27.9, 25.0, 24.4. HRMS (ESI) calculated for $C_{50}H_{64}N_{14}NaO_9$ $(M+Na)^+$: 1027.4910; found, 1027.4905.

Synthesis of compound PBP1:

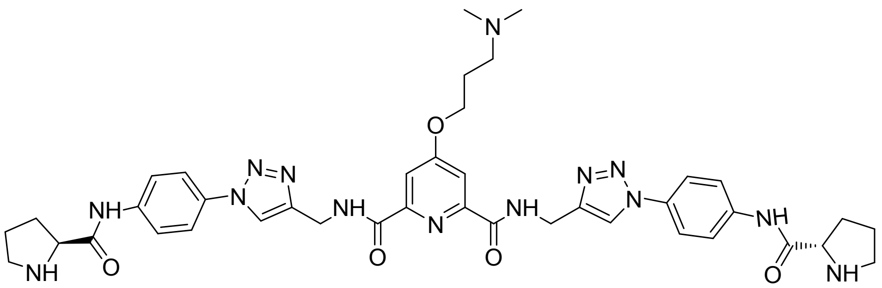

To an ice cooled solution of compound **S3** (250.0 mg, 0.24 mmol, 1.0 equiv.) in 10.0 mL dry CH_2Cl_2, added TFA (0.2 mL, 1.7 mmol, 6.0 equiv.) and the mixture was stirred for 6 h at room temperature. After consumption of the starting material **S3** (monitored by TLC), the reaction mixture was brought to pH 8-9 by adding dropwise a solution of liquid NH_3 (30 %) at 0 °C. Then the reaction mixture was extracted with CH_2Cl_2 (3 x 20 mL), dried in vacuum, purified by column chromatography using CH_2Cl_2:MeOH:NH_3 (90:09:1 to 75:20:5) to give **PBP1** (193 mg, 96 %) as a yellow solid; m.p. 230-232 °C. ^{1}H-NMR (500 MHz, DMSO-d$_6$): 10.24 (s, 2H), 9.95-9.93 (m, 2H), 8.62 (s, 2H), 7.86-7.83 (m, 8H), 7.72 (m, 2H), 4.72 (s, 4H), 4.26 (t, 2H, J = 4.8 Hz), 3.79-3.77 (m, 2H), 2.96 (s$_{br}$, 4H), 2.43 (t, 2H, J= 5.6 Hz), 2.19 (s, 6H), 2.12-2.10 (m, 2H), 1.94 – 1.83 (m, 4H), 1.74-1.70 (m, 4H). ^{13}C-NMR (125 MHz, DMSO-d$_6$): 173.8, 167.4, 163.4, 150.8, 146.0, 138.8, 132.1, 121.2, 120.7, 120.2, 110.6, 67.1, 60.9, 55.4, 46.9, 45.2, 34.7, 30.6, 26.4, 25.9. HRMS (ESI) calculated for $C_{40}H_{48}N_{14}NaO_5$ (M+Na)$^+$: 827.3842; found, 827.3839.

Synthesis of compound PBP2:

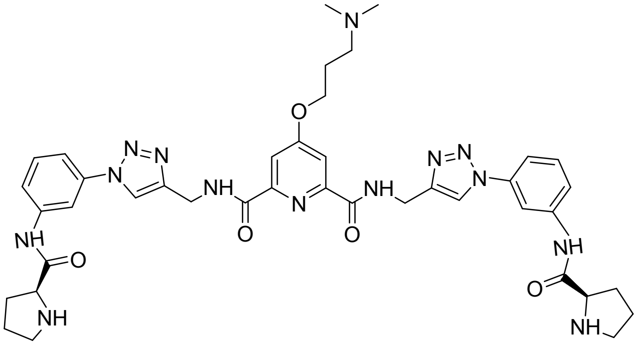

To an ice cooled solution of compound **S4** (250.0 mg, 0.3 mmol, 1.0 equiv.) in 10.0 mL dry CH_2Cl_2, added TFA (0.2 mL, 1.7 mmol, 6.0 equiv.) and the mixture was stirred for 6 h at room temperature. After consumption of the starting material **S4** (monitored by TLC), the reaction mixture was brought to pH 8-9 by adding drop wise a solution of liquid NH_3 (30 %) at 0 °C. Then the reaction mixture was extracted with CH_2Cl_2 (3 x 20 mL), dried in vacuum, purified by column chromatography using CH_2Cl_2:MeOH:NH_3 (90:09:1 to 75:20:5) to give **PBP2** (190 mg, 97 %) as a light brown solid. ^{1}H-NMR

(500 MHz, DMSO-d_6): 9.97 (s, 2H), 8.67 (s, 2H), 8.59 (s, 2H), 8.24 (s, 2H), 7.65 (s, 2H), 7.59 (d, 2H, *J* = 9.3 Hz), 7.54-7.48 (m, 4H), 4.64 (s, 4H), 4.35 (s, 2H), 4.24 (s, 2H), 3.22- 3.13 (m, 6H), 2.73 (s, 6H), 2.40-2.35 (m, 2H), 2.15 -2.09 (m, 4H), 1.96-1.85 (m, 6H); ^{13}C-NMR (125 MHz, DMSO-d_6): 175.0, 167.4, 163.5, 150.8, 146.2, 139.4, 130.7, 121.6, 121.4, 118.7, 115.9, 110.7, 110.5, 69.9, 66.1, 55.3, 55.0, 44.9, 34.6, 27.5, 26.1, 24.8. HRMS (ESI) calculated for $C_{40}H_{48}KN_{14}O_5$ $(M+K)^+$: 843.3674; found, 827.3672.

4.0 NMR spectra of compounds

NMR of *meta*-azido prolinamide **2**:

^{1}H-NMR:

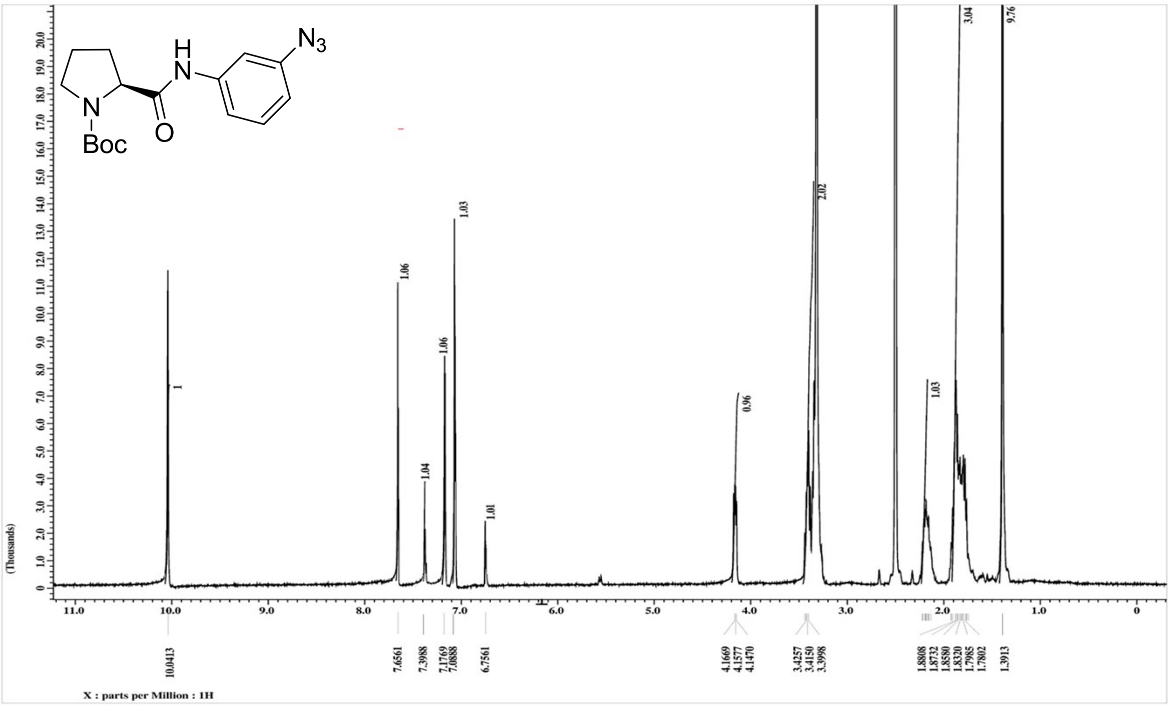

^{13}C-NMR:

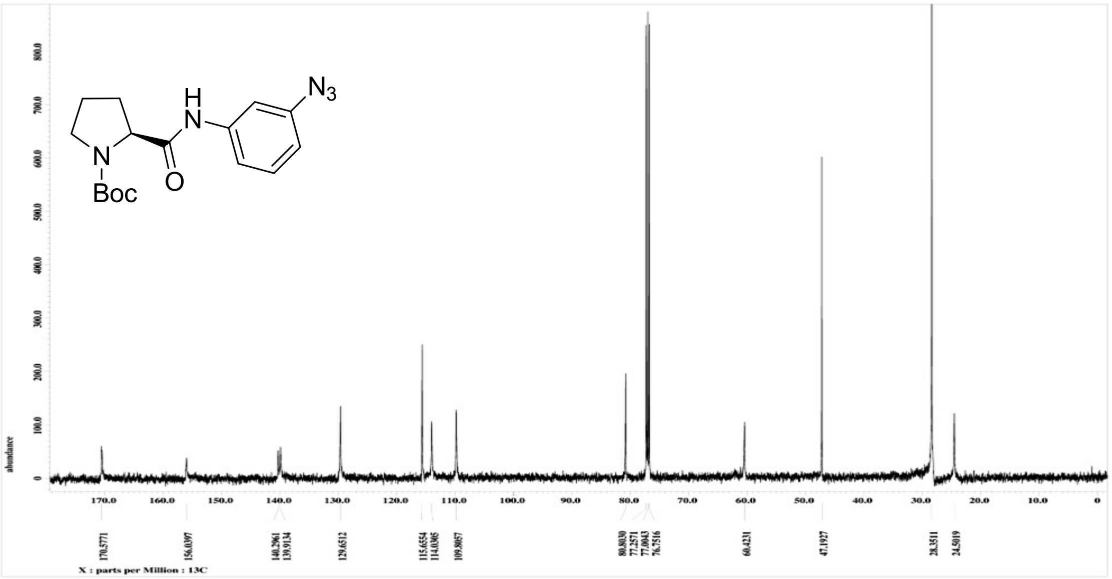

NMR of S2:

^{1}H-NMR:

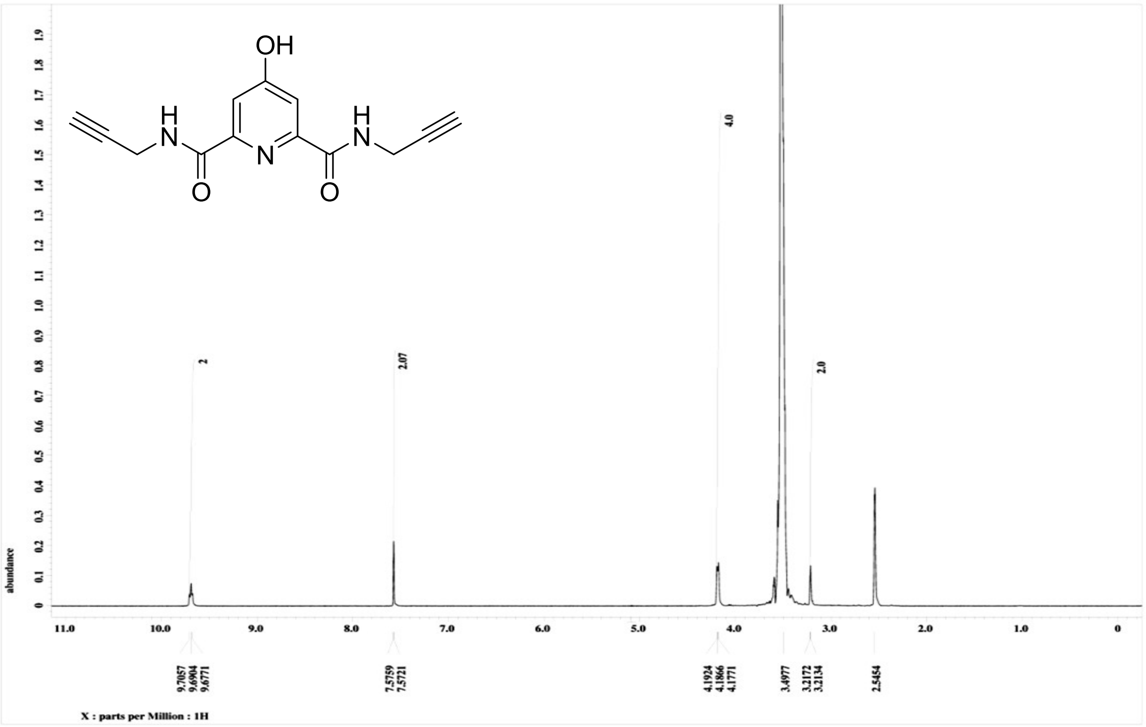

^{13}C-NMR:

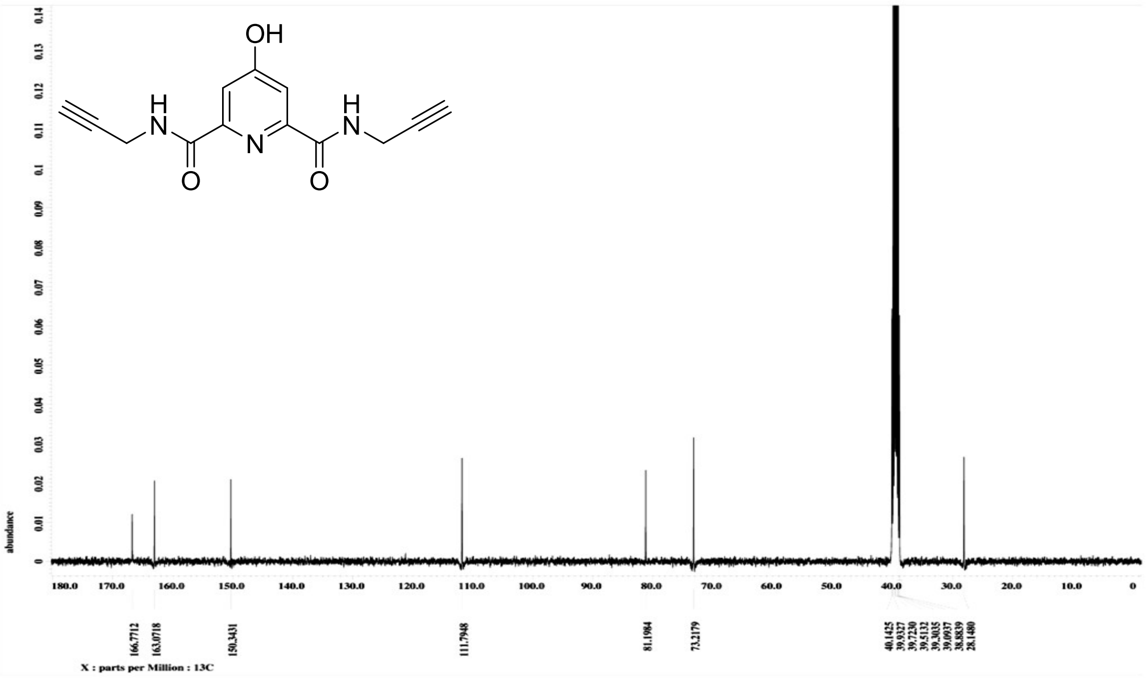

NMR of compound 3:

1H-NMR:

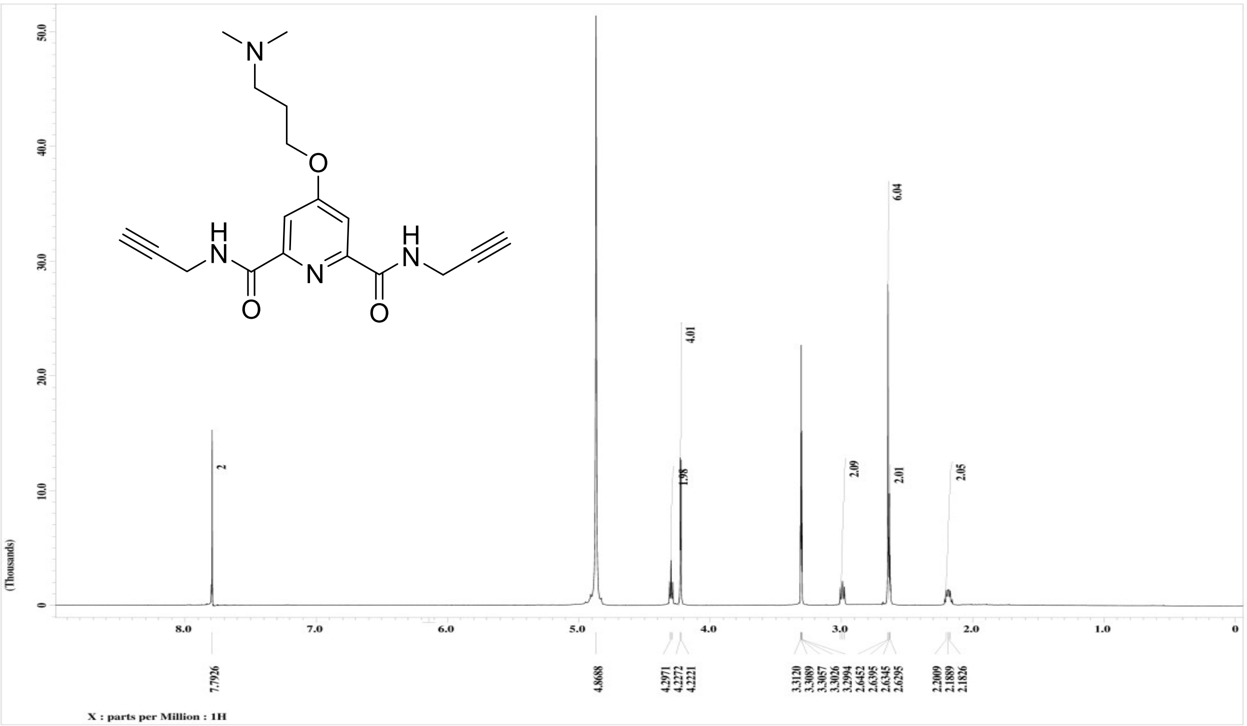

^{13}C-NMR:

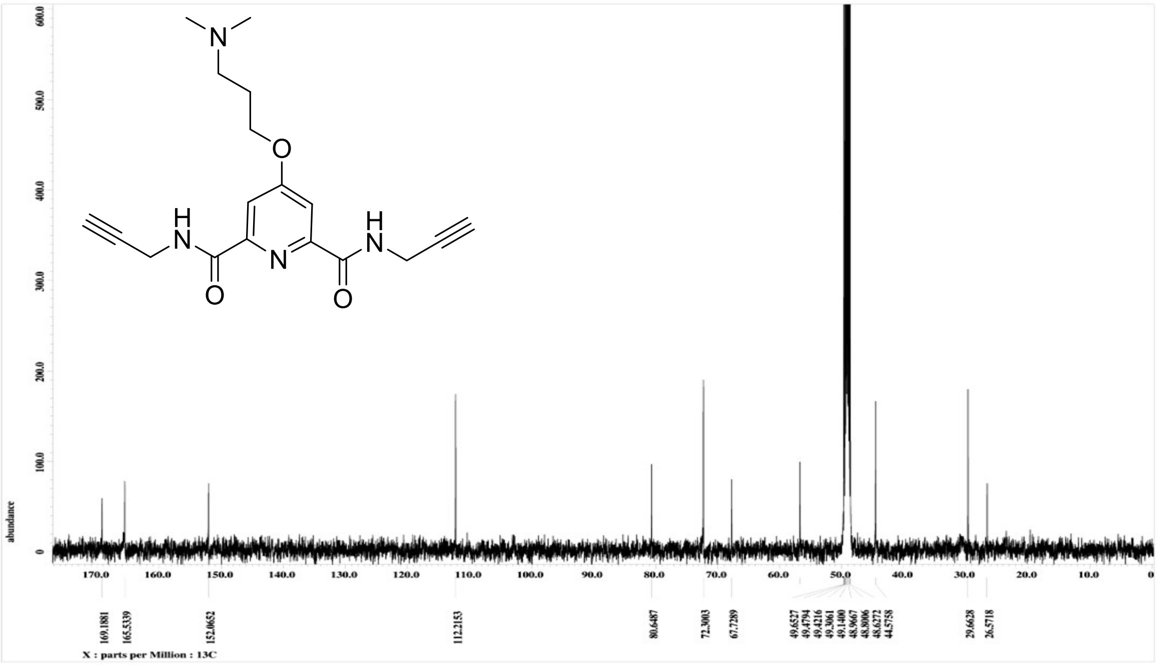

NMR of compound S3:

^{1}H-NMR:

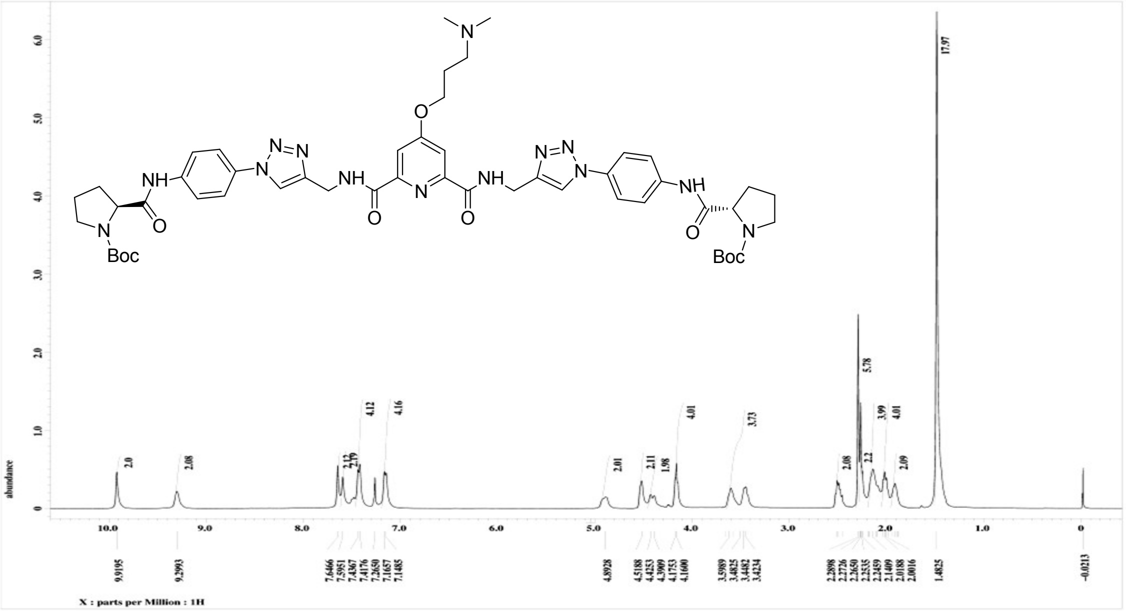

^{13}C-NMR:

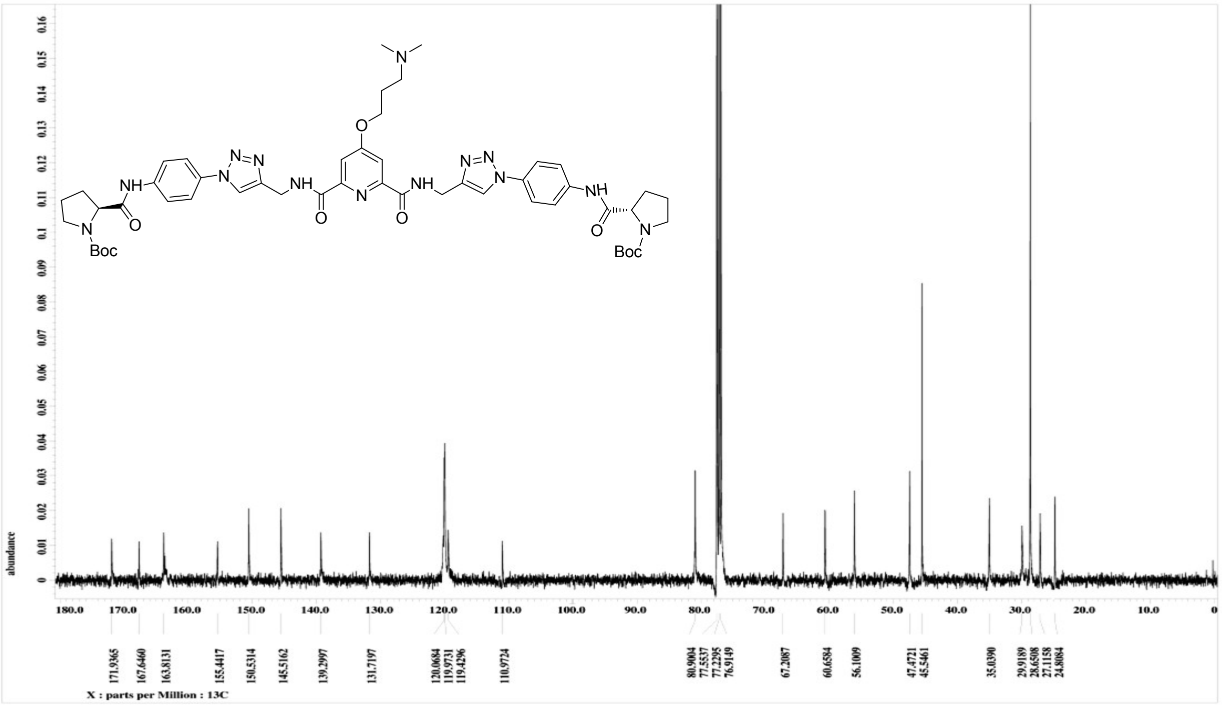

NMR of PBP1

^{1}H-NMR:

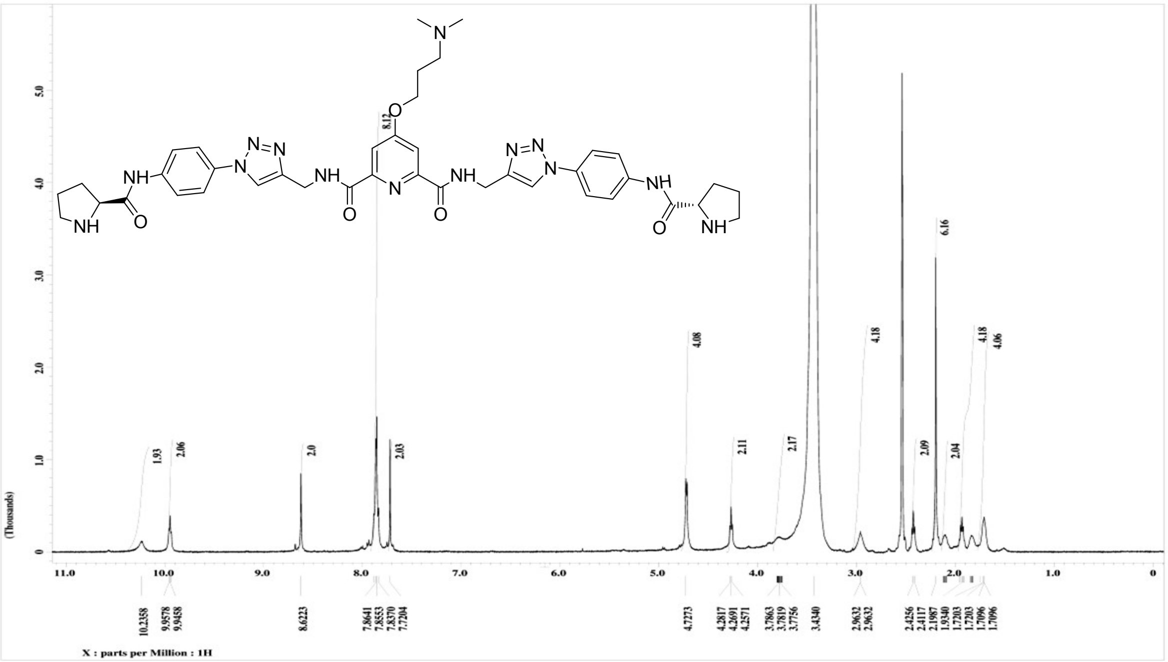

^{13}C-NMR:

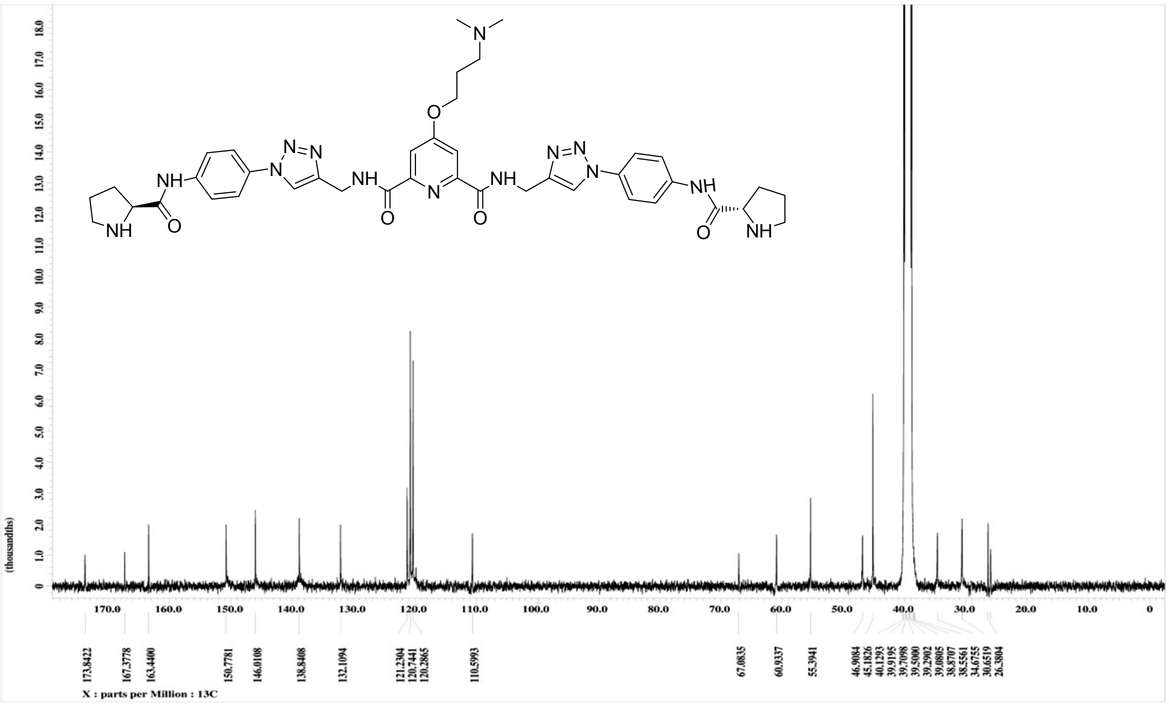

NMR of compound S4:

^{1}H-NMR:

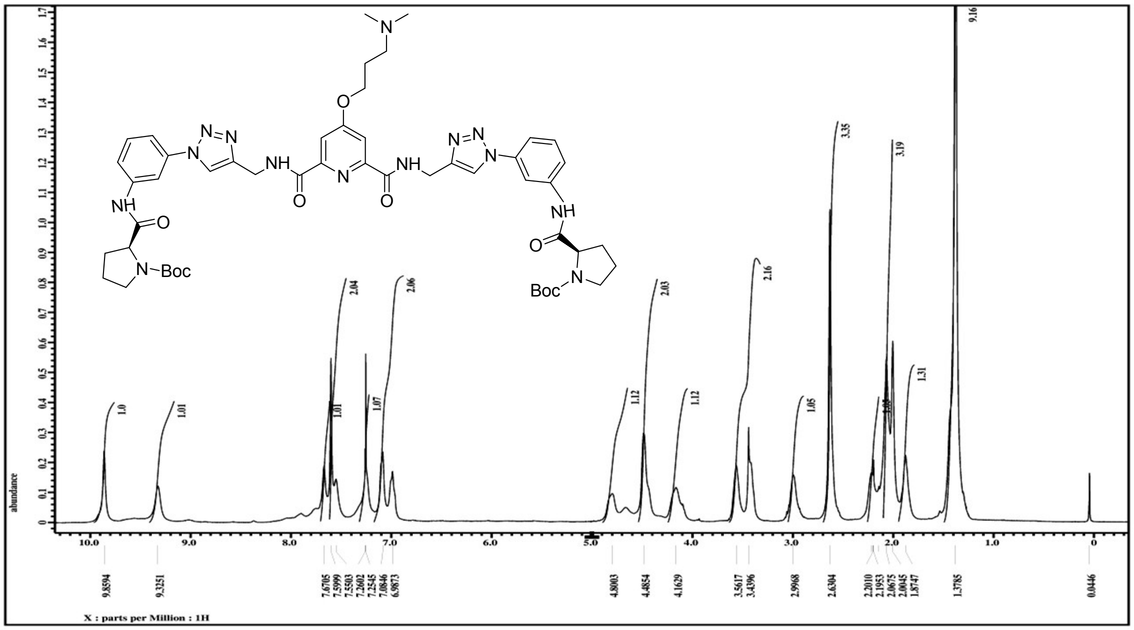

^{13}C-NMR:

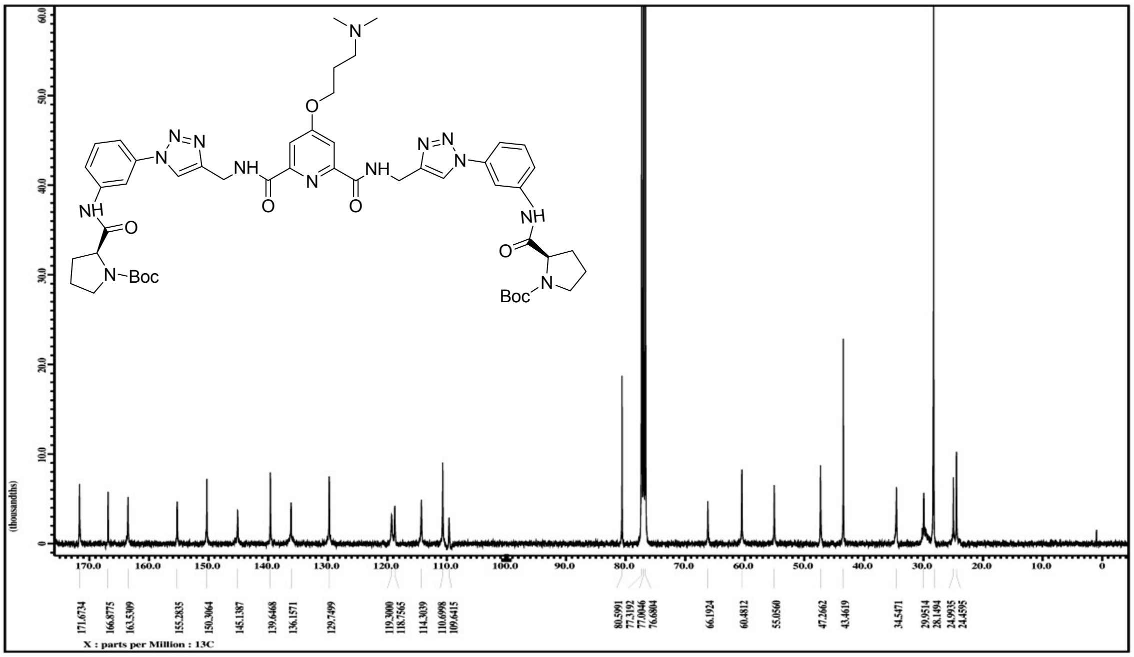

NMR of compound PBP2:

^{1}H-NMR:

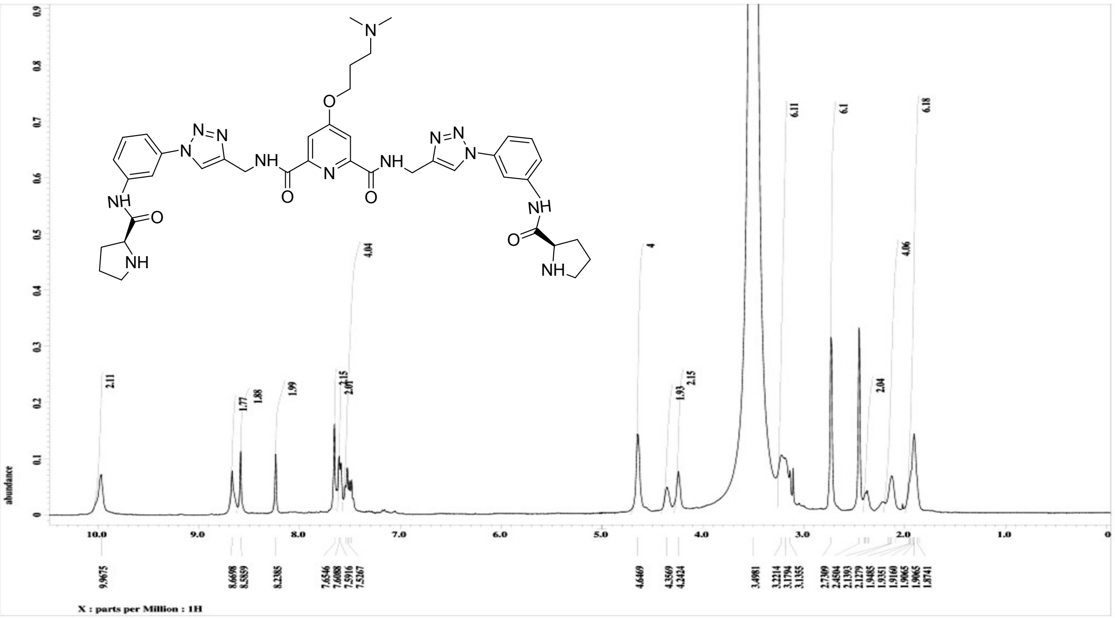

^{13}C-NMR

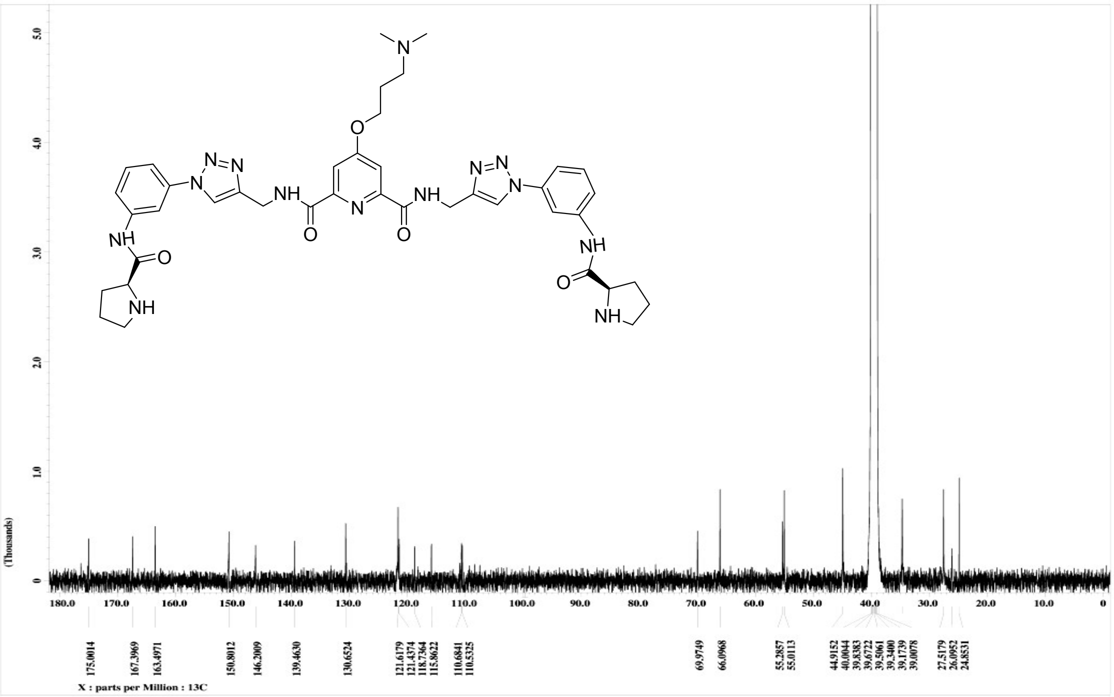

5.0 HPLC analysis:

HPLC chromatogram for PBP1

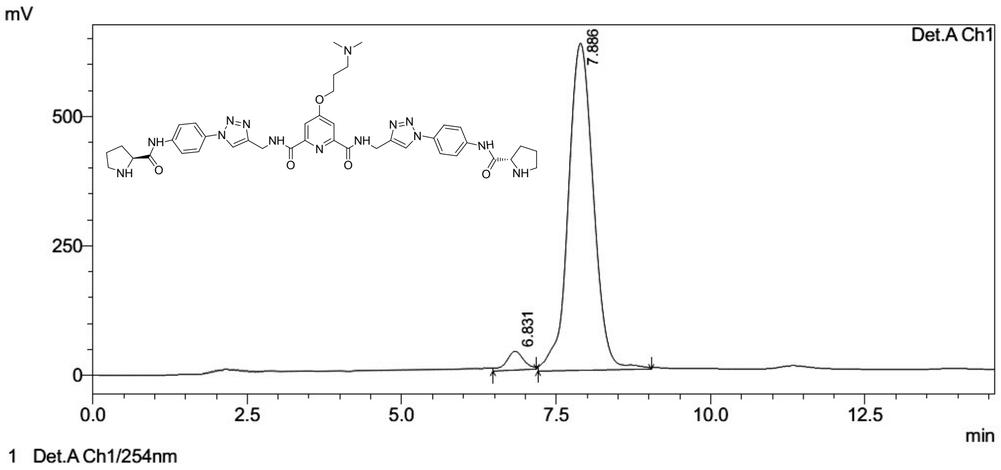

PeakTable

Detector A Ch1 254nm

Peak#	Ret. Time	Area	Height	Area %	Height %
1	6.831	715717	36313	3.826	5.435
2	7.886	17990680	631787	96.174	94.565
Total		18706398	668099	100.000	100.000

HPLC chromatogram for PBP2

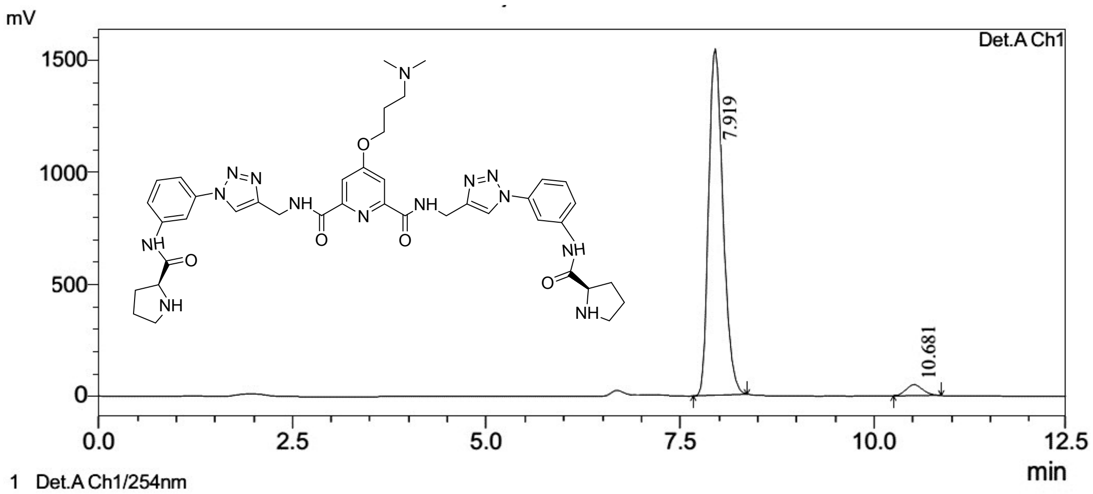

PeakTable

Detector A Ch1 254nm

Name	Ret. Time	Area	Height	Area %
1	7.919	6942133	1545943	96.396
2	10.681	259543	49343	3.604
		7201676	1595286	100.000

6.0 Melting analysis by Förster Resonance Energy Transfer (FRET). FRET melting assay was performed using a previously described method with small modifications.[2,3] 5 µM of dual labeled C-rich *BCL-2*-C, *c-MYC*-C, *h-TELO*-C, mutated *BCL-2*-C and mutated *c-MYC*-C sequences were heat annealed at 95 ºC in 60 mM K-cacodylate buffer (pH 4.8). These annealed sequences were then diluted in 60 mM K-cacodylate buffer to a final concentration of 200 nM and pH adjusted to 6. Similarly, 5 µM of dual labeled G-rich sequences *BCL-2*-G, *c-MYC*-G, *h-TELO*-G and the hairpin *ds* DNA sequence were heat annealed into G-quadruplexes in 60 mM K-cacodylate buffer (pH 7) and subsequently diluted in the same buffer to a final concentration of 200 nM. Stock solutions having 20 µM concentrations of **PBP1** and **PBP2** were prepared in 60 mM K-cacodylate buffer (final pH 7). The 96-well plates were prepared by aliquoting the annealed DNA (final concentration 100 nM) into each well, followed by the addition of increasing concentrations of **PBP1** and **PBP2** (0-10 µM). The pH was adjusted for C-rich DNA at 6, while the pH of G-rich and *ds* DNA were kept at 7. To perform competition experiments, dual labeled C-rich and G-rich sequences were heat annealed as described before to form i-motifs and G-quadruplexes, respectively. These folded i-motifs and quadruplexes were diluted to 200 nM in 60 mM K-cacodylate buffer and pH adjusted to 6 and 7 for i-motifs and G-quadruplexes, respectively. In dual labeled G-quadruplexes and i-motifs, 0-40 mol equivalent excess of heat annealed competitor G-quadruplex $(TG_5T)_4$ and duplex (ds26) DNA were added. The concentration of **PBP1** and **PBP2** were kept at 1.0 µM. Measurements were made in triplicate with an excitation wavelength of 483 nm and a detection wavelength of 533 nm using a LightCycler® 480-II RT-PCR machine (Roche). Final analysis of the data was carried out using Origin Pro 8 data analysis.

Sequences used in this study:

BCL-2-C: 5′-FAM-d($CAGC_4GCTC_3GC_5T_2C_2TC_3GCGC_3GC_4T$)-TAMRA-3′

BCL-2-G: 5′-FAM-d($AG_4CG_3CGCG_3AG_2A_2G_5CG_3AGCG_4CTG$)-TAMRA-3′

c-MYC-C: 5′-FAM-d($TC_4AC_2T_2C_4AC_3TC_4AC_3TC_4A$)-TAMRA-3′

c-MYC-G: 5′-FAM-d($TG_4AG_3TG_4AG_3TG_4A_2G_2TG_4A$)-TAMRA-3′

h-TELO-C: 5′-FAM-d[$TA_2C_3TA_2C_3TA_2C_3TA_2C_3$]-TAMRA-3′

h-TELO-G: 5′-FAM-d($G_3TTAG_3TTAG_3TTAG_3$)-TAMRA-3′

BCL-2-C-mut: 5′-FAM-d($CAGC_2TCGCTC_2TGC_2TC_2T_2C_2TC_2TGCGC_2TGC_2TCG$)-TAMRA-3′

c-MYC-C-mut: 5′-FAM-d($TC_2TCAC_2T_2CAC_2ACGCTCTC_2ACGCTCTC_2A$)-TAMRA-3′

---

[2] J.-L. Mergny, *Biochemistry*, 1999, **38**, 1573-1581.
[3] H. A. Day, C. Huguin and Z. A. E. Waller, *Chem. Commun.*, 2013, **49**, 7696-7698.

ds DNA: 5′-FAM-d(TAT AGC TAT A HEG TAT AGC TAT A)-TAMRA-3′

HEG stands for hexaethylene glycol.

ds26: 5′-CAATCGGATCGA ATTCGATCCGATTG-3′

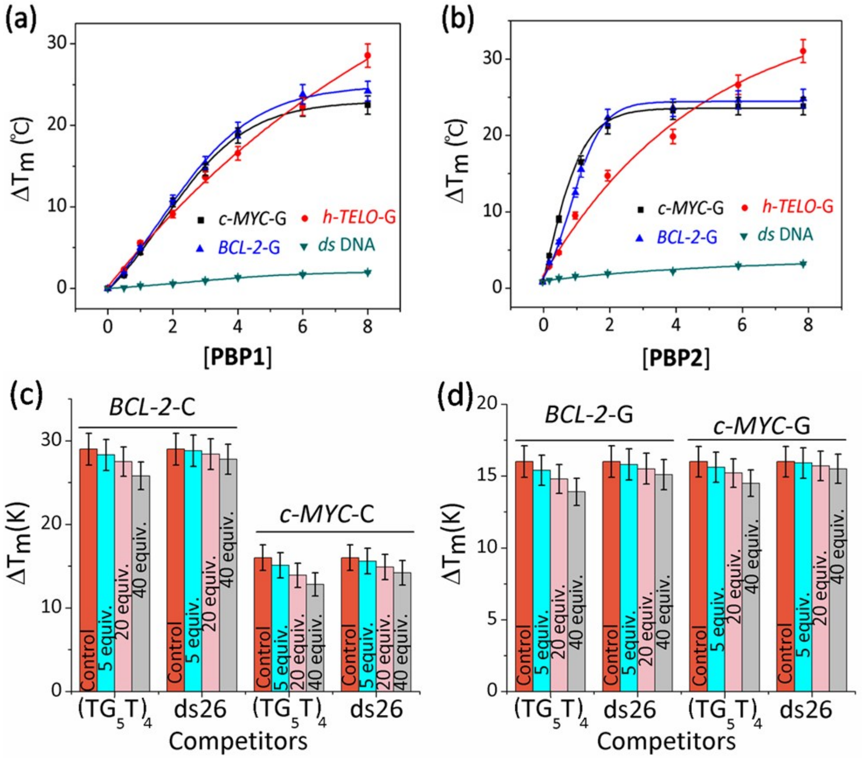

Fig. S2 Thermal shift profiles for increasing concentrations of (a) **PBP1** and (b) **PBP2** upon stabilizing pre-folded G-quadruplexes and hairpin *ds* DNA; 60 mM K-cacodylate buffer (pH 7). FRET competition of (c) **PBP1** bound dual labeled *BCL-2*-C and *c-MYC*-C i-motifs in presence of competitor G-quadruplex $(TG_5T)_4$ and duplex (ds26) DNA (60 mM K-cacodylate buffer, pH 6) and (d) **PBP2** bound dual labeled *BCL-2*-G and *c-MYC*-G G-quadruplexes in presence of competitor tetramolecular G-quadruplex $(TG_5T)_4$ and duplex (ds26) DNA (60 mM K-cacodylate buffer, pH 7).

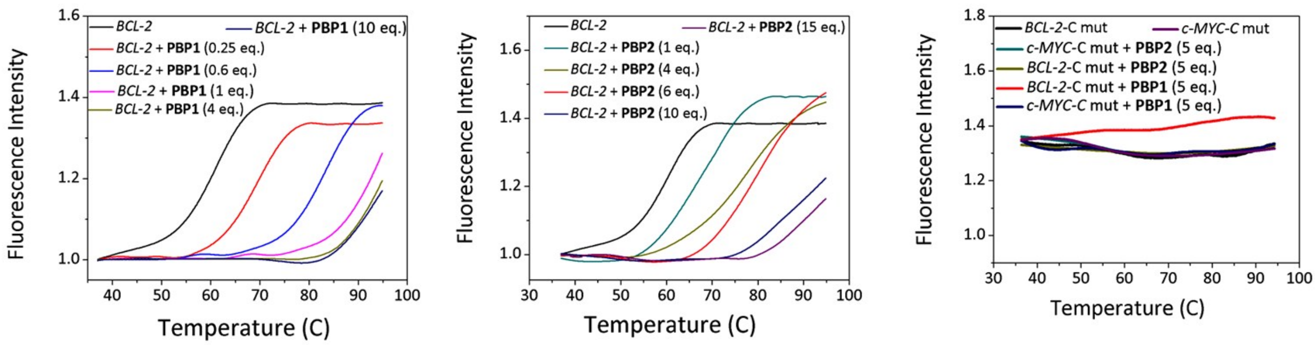

Fig. S3 Raw melting curves for titration of dual labeled pre-folded *BCL-2*-C i-motifs in 60 mM K-cacodylate buffer, (pH 6) with increasing concentrations of (a) **PBP1** (0-10 equiv.); (b) **PBP2** (0-15

equiv.); (c) Raw melting curves for titration of dual labeled mutated *BCL-2*-C sequences in 60 mM K-cacodylate buffer, (pH 6) with increasing concentrations of **PBP1** (5 equiv.) and **PBP2** (5 equiv.).

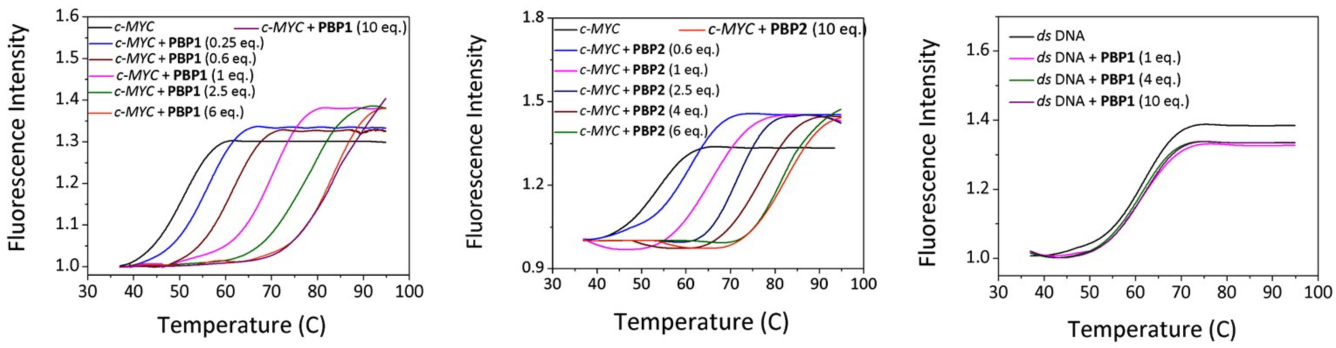

Fig. S4 Raw melting curves for titration of dual labeled pre-folded *c-MYC*-C i-motifs diluted in 60 mM K-cacodylate buffer (pH 6) with increasing concentrations of (a) **PBP1** (0-10 equiv.); (b) **PBP2** (0-10 equiv.). (c) Raw melting curves for titration of dual labeled *ds* DNA diluted in 60 mM K-cacodylate buffer (pH 7) with increasing concentrations of **PBP1** (0-10 equiv.).

Table S1. Dual labeled sequences used in this study and their ΔT_m values at 1 μM ligand concentration.

**DNA Sequences**	**ΔT_m[a] in °C**		
	**PBP1**	**PBP2**	**PBP3**
BCL-2-C: 5′-*FAM*-d($CAGC_4GCTC_3GC_5T_2C_2TC_3GCGC_3GC_4T$)-*TAMRA*-3′	29	8	4.1
BCL-2-G: 5′-*FAM*-d($AG_4CG_3CGCG_3AG_2A_2G_5CG_3AGCG_4CGT$)-*TAMRA*-3′	5.2	16	5.3
c-MYC-C: 5′-*FAM*-d($TC_4AC_2T_2C_4AC_3TC_4AC_3TC_4A$)-*TAMRA*-3′	16	8	5.2
c-MYC-G: 5′-*FAM*-d($TG_4AG_3TG_4AG_3TG_4A_2G_2TG_4A$)-*TAMRA*-3′	5.2	16	3.4
h-TELO-C: 5′-*FAM*-d($TA_2C_3TA_2C_3TA_2C_3TA_2C_3$)-*TAMRA*-3′	9	5	3.5
h-TELO-G: 5′-*FAM*-d($G_3TTAG_3TTAG_3TTAG_3$)-*TAMRA*-3′	5	8	3.1
ds DNA: 5′-*FAM*-d(TATAGCTATA-HEG-TATAGCTATA)-*TAMRA*-3′	0.94	1.1	0.7
[a]*BCL-2*-C-mut:5′-FAM-d($CAGC_2TCGCTC_2TGC_2TC_2T_2C_2TC_2TGCGC_2TGC_2TCG$)-TAMRA-3′	-	-	-
[a]*c-MYC*-C-mut:5′-FAM-d($TC_2TCAC_2T_2CAC_2ACGCTCTC_2ACGCTCTC_2A$)-TAMRA-3′	-	-	-

[a]ΔT_m = ±1 °C; T_m of pre-folded *c-MYC*-C, *BCL-2*-C, *h-TELO*-C and *ds* DNA diluted in 60 mM K-cacodylate buffer, (final pH 6) are 48 ± 1 °C, 59 ± 1 °C, 43 ± 1 °C and 60 ± 1 °C, respectively. T_m of pre-folded *c-MYC*-G, *BCL-2*-G and *h-TELO*-G diluted in 60 mM K-cacodylate buffer, pH 7 is 69 ± 1 °C, 70 ± 1 °C, 55 ± 1 °C. See experimental section for folding conditions. The mutant C-rich sequences primarily exist in the unfolded form as evident from absence of sigmoidal melting curve under experimental conditions.

7.0 TO Displacement assay:

Thiazole orange (TO) Displacement assay was performed with pre-folded quadruplexes and pre-folded i-motifs. First, 10 µM of unlabeled *BCL-2*-C and *c-MYC*-C i-motif forming sequences were folded in 60 mM K-cacodylate buffer, pH 4.8 by heat annealing. These folded i-motifs were diluted to 0.25 µM in 60 mM K-cacodylate buffer (pH adjusted to 6). Similarly, 10 µM of unlabeled *BCL-2*-G and *c-MYC*-G G-quadruplex sequences and a hairpin *ds* DNA were folded in 60 mM K-cacodylate buffer (pH 7) and diluted to 0.25 µM in the same buffer. To the diluted 0.25 µM solution of pre-folded DNA, TO was added to the final concentration of 0.5 µM. The DNA-TO mixture was then titrated with ligands **PBP1**, **PBP2** or **PBP3** (20 µM stock in respective buffer) and the fluorescence spectrum was recorded after a 3-min equilibration period in Horiba JobinYvonFluorolog 3 fluorescence spectrometer. The percentage of displacement was calculated from the fluorescence area (FA, 510-700 nm, λ_{ex} = 501 nm), using,

$$TO\ displacement\ (\%) = 100 - \left[FA \times 100/FA_0\right] \ldots\ldots \quad (S1)$$

FA_0 being the fluorescence area from TO bound to DNA without added ligand. The percentages of TO displacement were then plotted as a function of the concentration of the ligands.

Sequences used in this study:

BCL-2-C: 5′-d($CAGC_4GCTC_3GC_5T_2C_2TC_3GCGC_3GC_4T$)-3′

BCL-2-G: 5′-d($AG_4CG_3CGCG_3AG_2A_2G_5CG_3AGCG_4CTG$)-3′

c-MYC-C: 5′-d($TC_4AC_2T_2C_4AC_3TC_4AC_3TC_4A$)-3′

c-MYC-G: 5′-d($TG_4AG_3TG_4AG_3TG_4A_2G_2TG_4A$)-3′

h-TELO-C: 5′-d[$TA_2C_3TA_2C_3TA_2C_3TA_2C_3$]-3′

h-TELO-G: 5′-d($G_3TTAG_3TTAG_3TTAG_3$)-3′

ds DNA: 5′-d(TAT AGC TAT A HEG TAT AGCTAT A)-3′

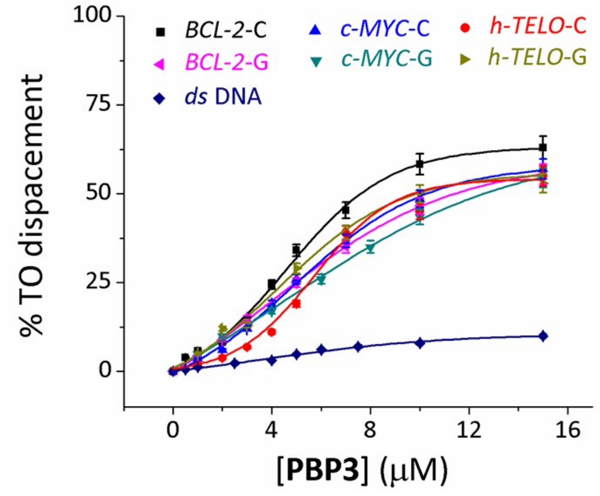

Fig. S5 TO displacement from pre-folded *BCL-2*-C, *c-MYC*-C, *h-TELO*-C i-motifs in 60 mM K-cacodylate (pH 6), pre-folded *BCL-2*-G, *c-MYC*-G, *h-TELO*-G G-quadruplexes in 60 mM K-cacodylate, pH 7 and hairpin *ds* DNA; with increasing concentrations of **PBP3** (0-15 μM).

We observed a dose dependent quenching upon addition of the ligands to the TO-bound G-quadruplexes and i-motifs indicating the displacement of TO from the DNA by the ligands. As **PBP 1-3** are non-fluorescent and since they do not influence TO fluorescence (evident from lack of quenching upon addition of **PBP1-3** in TO bound duplex DNA), the only cause of decrease in fluorescence intensity is due to the TO displacement by ligand.

Table S2. DC_{50} obtained for bis-prolinamides from TO displacement.

**DNA Sequences**	**DC_{50} (μM)[a]**		
	**PBP1**	**PBP2**	**PBP3**
BCL-2-C: 5′- d($CAGC_4GCTC_3GC_5T_2C_2TC_3GCGC_3GC_4T$)-3′	0.9	8.2	7.9
BCL-2-G: 5′- d($AG_4CG_3CGCG_3AG_2A_2G_5CG_3AGCG_4CGT$)-3′	5.7	2.4	9
c-MYC-C: 5′- d($TC_4AC_2T_2C_4AC_3TC_4AC_3TC_4A$) -3′	2.7	6.8	8.4
c-MYC-G: 5′- d($TG_4AG_3TG_4AG_3TG_4A_2G_2TG_4A$) -3′	8.5	1.3	10.2
h-TELO-C: 5′- d($TA_2C_3TA_2C_3TA_2C_3TA_2C_3$) -3′	4	>15	10
h-TELO-G: 5′- d($G_3TTAG_3TTAG_3TTAG_3$) -3′	9.8	4.7	8.4
ds DNA: 5′- d(TATAGCTATA-HEG-TATAGCTATA) -3′	n.d.	n.d.	n.d.

[a]Errors were calculated as standard deviation of three independent experiments. Error (± 5 %)

8.0 Fluorescence binding experiments

Fluorescence quenching assay was performed following a previously described method.[4] The fluorescence spectra were recorded on a Horiba JobinYvonFluorolog 3 instrument at 25 °C in a thermostated cell holder using quartz cuvette with a 1 cm path-length. 10 µM of 5′-TAMRA or 3′-TAMRA labeled *BCL-2*-C and *c-MYC*-C i-motif forming sequences were folded in 60 mM K-cacodylate (pH 4.8) buffer by heat annealing at 95 ºC and subsequently diluted to 250 nM in 60 mM K-cacodylate buffer (pH adjusted to 6). Similarly, 10 µM of 5′-TAMRA and 3′-TAMRA labeled *BCL-2*-G and *c-MYC*-G G-quadruplex forming sequences and hairpin *ds* DNA were folded in 60 mM K-cacodylate buffer (pH 7) followed by dilution to 250 nM in the same buffer. The 250 nM labeled i-motifs or G-quadruplexes or *ds* DNA were titrated with ligands **PBP 1-3** (500 µM stock in respective buffer) and the fluorescence emission spectra were recorded after a 2 min equilibration period. Fluorescence excitation was set at 520 nm and the emission was monitored at 582 nm. Bandwidths of both excitation and emission filters were set at 4 nm. Fluorescence quenching was observed after the incremental addition of **PBP 1-3**. The recorded spectral data was used to determine the dissociation constant of the ligands for quadruplexes and i-motifs using the Hill formula (2),[5]

$$\%\,Fluorescence\ quenching = V_{max}\frac{[Ligand]^n}{K_d^n + [Ligand]^n} \ldots\ldots\ldots\ldots (S2)$$

Where, K_d is the dissociation constant. “n” stands for Hill coefficient and was taken as “1”.

Following sequences, labeled with TAMRA at either 5′- or -3′ end were used in this study,

BCL-2-C: 5′-d($CAGC_4GCTC_3GC_5T_2C_2TC_3GCGC_3GC_4T$)-3′

BCL-2-G: 5′-d($AG_4CG_3CGCG_3AG_2A_2G_5CG_3AGCG_4CTG$)-3′

c-MYC-C: 5′-d($TC_4AC_2T_2C_4AC_3TC_4AC_3TC_4A$)-3′

c-MYC-G: 5′-d($TG_4AG_3TG_4AG_3TG_4A_2G_2TG_4A$)-3′

ds DNA: 5′-d(TAT AGC TAT A HEG TAT AGC TATA)-3′

[4] D. D. Le, M. Di Antonio, L. K. M. Chan and S. Balasubramanian, *Chem. Commun*., 2015, **51**, 8048-8050.

[5] R. Tippana, W. Xiao and S. Myong, *Nucleic Acids Res.*, 2014, **42**, 8106-8114.

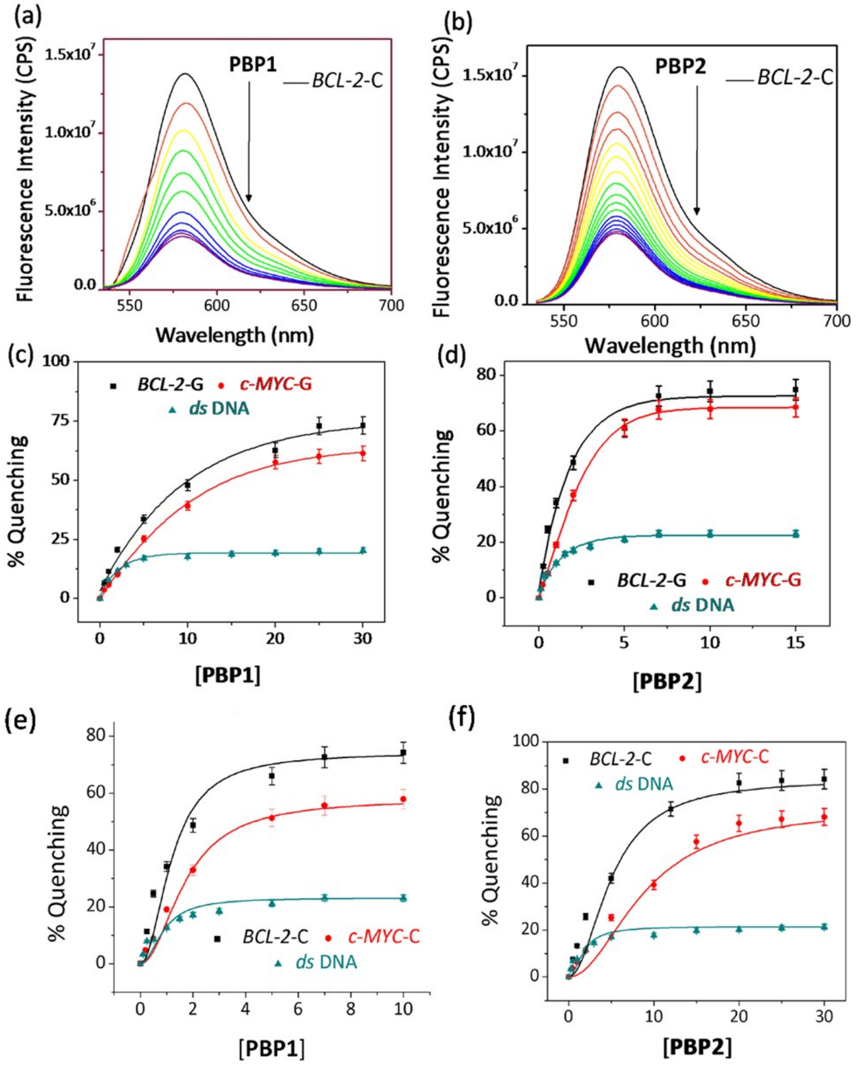

Fig. S6 (a) Fluorescence emission spectra of the 250 nM of 5´-TAMRA labeled pre-folded *BCL-2*-C diluted in 60 mM K-cacodylate (pH adjusted to 6) with incremental addition of (a) **PBP1** (0-15 μM); (b) **PBP2** (0-30 μM). The percentage quenching observed for 250 nM of 3´-TAMRA labeled pre-folded *BCL-2*-G and *c-MYC*-G G-quadruplexes in the presence of **PBP1** (0-15 μM) (c) and **PBP2** (0-30 μM) (d) diluted in 60 mM K-cacodylate, pH 7. The percentage quenching observed for 250 nM of 3´-TAMRA labeled pre-folded *BCL-2*-C and *c-MYC*-C i-motifs in the presence of (e) **PBP1** (0-10 μM) and (f) **PBP2** (0-30 μM) in 60 mM K-cacodylate, pH 6.

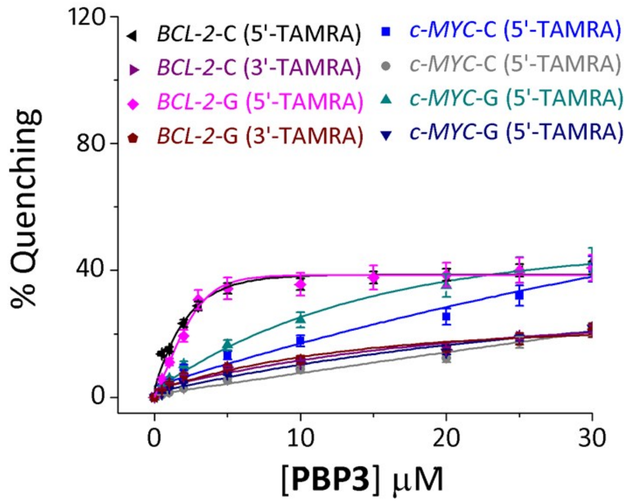

Fig. S7 The percentage quenching observed for 250 nM of 5′- and 3′-TAMRA labeled pre-folded *BCL-2*/*c-MYC* i-motifs and G-quadruplexes in the presence of **PBP3** (0-30 μM) in 60 mM K-cacodylate of pH 6 for i-motifs and pH 7 for G-quadruplexes. Since **PBP3** quenched the fluorescence intensity of 5′- and 3′-TAMRA labeled G-quadruplexes and i-motifs by less than 40 %, the K_d values cannot be reliably determined from this assay.

Table S3. Table representing the dissociation constant (K_d) values for PBP1 and PBP2 with 5′-TAMRA labeled DNA.

DNA	PBP1 (μM)	PBP2 (μM)
5′-TAMRA-*BCL-2*-C	0.3	5.8
5′-TAMRA-*c-MYC*-C	2.4	9.5
5′-TAMRA-*ds* DNA	>25	>25
5′-TAMRA-*BCL-2*-G	7.2	1.9
5′-TAMRA-*c-MYC*- G	12.5	1.3
K_d = ± 5 %		

Table S4. Table representing the dissociation constant (K_d) values for PBP1 and PBP2 with 3′-TAMRA labeled DNA.

DNA	PBP1 (μM)	PBP2 (μM)
3′-TAMRA-*BCL-2*-C	2.1	10.5
3′-TAMRA-*c-MYC*-C	5.9	15.1
3′-TAMRA-*BCL-2*- G	11.5	4.5
3′-TAMRA-*c-MYC*- G	16.7	4.1
** To understand the binding preferences of **PBP1** and **PBP2**, fluorescence titrations with 3′-TAMRA labeled *BCL-2*-C and *c-MYC*-C i-motif were carried out. The K_d values of **PBP1** and **PBP2** for 3′-TAMRA-*BCL-2*-C i-motif were determined to be 2.1 μM and 10.5 μM, respectively. **PBP1** and **PBP2** exhibited higher K_d values of 4.1 μM and 16.7 μM, respectively for 3′-TAMRA-*c-MYC*-C i-motif. Furthermore, the K_d values obtained for the 5′-labeled sequences are lower compared to the 3′-labeled sequences, which indicate that bis-prolinamide ligands preferentially bind to the proximity of 5′-end of i-motifs. K_d = ± 5 %.		

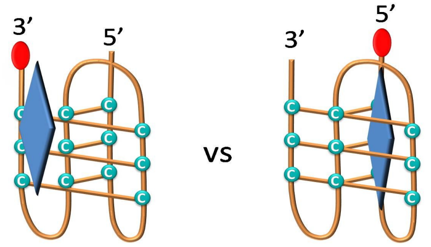

Scheme S2 Selectivity between 5′- and 3′-labeled i-motifs for ligand binding. Since i-motif is an antiparallel structure, an end stacking binding of ligands to the 5′- and 3′-labeled i-motifs would give similar level of quenching and similar K_d values. However, **PBP1** binds to 5′-labeled i-motifs with significantly lower K_d values compared to that of 3′-labeled i-motifs (Fig. 2, S6, Table S3, S4). This result indicates that ligands do not bind through end stacking mode. Furthermore, the addition of **PBP1** to i-motifs does not significantly change the CD peak position or intensity indicating an external binding mode of **PBP1** (Fig. S21a). Previous reports indicate that the Circular dichroism (CD) spectra shows almost no change in the case of external binding such as groove binding or electrostatic interactions, whereas both positive and negative bands are affected in case of intercalative or end-stacking binding mode (Y.-M. Chang, C. K.-M. Chen and M.-H.Hou, *Int. J. Mol. Sci.*, 2012, **13**, 3394-3413 and A. Manna and S. Chakravorti, *J. Phys. Chem. B.*, 2012, **116**, 5226-5233). Since Job's plot indicated 1:1 binding mode (Fig. S25 and S26, ESI), we speculated that **PBP1** might bind to i-motifs via the binding modes shown above. The significantly lower K_d value of **PBP1** upon binding to 5′-labeled i-motif compared to 3′-labeled i-motif indicated that **PBP1** binds to the proximity of 5′-end of the i-motifs (as shown above) and mediate efficient quenching of the labeled dye. In addition, TO displacement assay indicates that the binding of **PBP1** to the proximity of 5′-end of i-motif can displace TO from the ends of i-motifs to a significant extent (due to partial overlapping of **PBP1** and TO binding sites).

9.0 Confocal microscopy:

Single molecule FRET: The smFRET studies were carried out using 100 pM dual labeled *BCL-2*-C, *BCL-2*-C-mut, *BCL-2*-G, *BCL-2*-G-mut, *c-MYC*-C and *c-MYC*-G DNA sequences. These unfolded dual labeled sequences were dispersed in Milli-Q water (pH 7) and were used as free *BCL-2*-C, *BCL-2*-C-mut, *BCL-2*-G, *BCL-2*-G-mut, *c-MYC*-C and *c-MYC*-G sequences. The C-rich sequences were folded into i-motifs by heat annealing in 10 mM Na-cacodylate buffer, pH 4.8 and subsequently diluting them in 10 mM Na-cacodylate (pH 4.8) buffer. Similarly, the folded G-quadruplexes were obtained by heat annealing the G-rich sequences in 60 mM K-cacodylate buffer (pH 7) and subsequently diluting them in Milli-Q water (pH 7). Ligands **PBP1** and **PBP2** were prepared in Milli-Q water (pH adjusted to 7) and were added to the DNA in the final concentration of 1 equivalent (1 equiv.) of the DNA used. We have used the PicoQuant, Micro-Time 200 confocal setup with an inverted optical microscope (Olympus IX-71).[6] A pulsed diode laser with a repetition rate of ~ 40 MHz was used. The laser power was kept below

50 μW. Fluorescence from the labeled DNA was separated with a dichroic mirror (HQ490DCXR, Chroma). To block the exciting laser light, a suitable long-pass filter (510LP, Chroma, for excitation at 470 nm) was used before the detectors. The fluorescence was focused through a pinhole (30 μm). The donor (FAM) and the acceptor (TAMRA) fluorescence signals were captured separately using a dichroic mirror (540DCLP) and two single photon avalanche diodes (SPADs). Two additional band pass filters (FF01-520/35 for the donor and HQ580/35 for the acceptor) were used to further separate the donor and acceptor fluorescence.

For the FRET experiments on freely diffusing systems, the folded and unfolded DNA sequences, labeled with donor (FAM) and acceptor (TAMRA) passed through the confocal volume on a bare slide and the donor dye was excited by laser illumination. Fluorescence bursts of both donor and acceptor (excited via FRET) were recorded and from which the FRET efficiency was calculated. In this system (freely diffusing DNA), FRET analysis was limited by the transit time (binning time typically ~ 2.5 ms) through observation volume of dimension 235 nm [0.61 λ/numerical aperture, NA = 1.2, λ = 470 nm]. The total time collected to generate a single histogram was ~ 15 min. The background count was found to be ~ 500 counts/s and the average count for a burst was ≥8000 counts/s. In the MCS plots, a background of 500 counts/s was subtracted from the data to obtain background corrected images (Fig. 3 and Fig. S8). The average signal strength for a burst shows approximately 16X signal strength of the background. However, bursts having maximum signal strength <20000 counts/s were observed, indicating approximately <40X signal strength of the background.

Efficiency of FRET (ε_{FRET}) is given by,[7]

$$\varepsilon_{FRET} = \frac{I_A}{\gamma I_D + I_A} \text{.............(S3)}$$

Where, I_A and I_D are the background and cross-talk corrected acceptor and donor intensities. The correction factor γ may be expressed as,

$$\gamma = \frac{\varphi_A \eta_A}{\varphi_D \eta_D} \text{...........(S4)}$$

The value of γ is obtained from the quantum yield of donor (φ_D) and acceptor (φ_A) and the calibration curves for the detection efficiency of donor (η_D) and acceptor (η_A) SPADs. The value of γ is determined for each system separately considering change in quantum yields of both donor and acceptor labeled DNA at pH 4.8 or 7 in the presence and absence of **PBP1** and **PBP2**. The distance between the dye pairs (R_{DA}) is calculated from each FRET efficiency value from the relation,

---

[6] S. Ghosh, C. Ghosh, S. Nandi and K. Bhattacharyya, *Phys. Chem. Chem. Phys.*, 2015, **17**, 8017-8027.
[7] J. R. Lakowicz, Principles of fluorescence spectroscopy; 3rd ed.; Springer: New York, (2006).

$$R_{DA} = R_0 \left[\frac{1 - E}{E}\right]^{\frac{1}{6}} \text{.............(S5)}$$

R_0 (Förster distance) was determined from the spectral overlap, $J(\lambda)$, between the donor emission and the acceptor absorption as follows,

$$R_0 = 0.211\left[\kappa^2\eta^{-4}Q_D J(\lambda)\right]^{1/6} \text{...........}(S6)$$

$$J(\lambda) = \frac{\int_0^\infty F_D(\lambda)\varepsilon_A(\lambda)\lambda^4 d\lambda}{\int_0^\infty F_D(\lambda)d\lambda} \text{...........}(S7)$$

R_0 (Förster distance) for the FAM-TAMRA pair of fluorescent dye is 55 Å (assuming a rotational diffusion randomized to the dipole orientation factor κ^2 of 2/3)[8].

10.0 Donor shot noise data:

The smFRET experiments are often complicated by shot noise.[9,10] The contributions of shot noise in each FRET peak were calculated[11].

Table S5. FRET peak widths for *BCL-2*-C and *c-MYC*-C at different pH in the presence and absence of **PBP1** and **PBP2.**

System	Peak efficiency	σ_{sn}	σ_m	σ_m / σ_{sn}
BCL-2-C (pH 7)	0.64	0.18	0.25	1.09
	0.45	0.21	0.06	**0.29**
BCL-2-C (pH 4.8)	0.95	0.087	0.095	1.09
BCL-2-C (pH 7) + **PBP1**	0.92	0.10	0.23	2.3
BCL-2-C (pH 7) + **PBP2**	0.95	0.06	0.16	2.67
	0.67	0.12	0.16	1.33
c-MYC-C (pH 7)	0.80	0.15	0.24	1.6
	0.55	0.19	0.20	1.1
c-MYC-C (pH 4.8)	0.95	0.05	0.15	3
c-MYC-C (pH 7) + **PBP1**	0.93	0.11	0.18	1.64
c-MYC-C (pH 7) + **PBP1**	0.82	0.13	0.22	1.69
	0.57	0.17	0.29	1.7
BCL-2-G + **PBP2**	0.95	0.09	0.26	2.89
BCL-2-G + **PBP1**	0.61	0.09	0.15	1.7
	0.85	0.09	0.23	2.56

The width of the FRET efficiency peak from shot noise, $\boldsymbol{\sigma_{sn}}$, is given by,

$$\sigma_{sn} = \sqrt{\langle E_m\rangle\left(1 - \langle E_m\rangle\right)\langle N^{-1}\rangle} \text{............}(S8)$$

[8] R. M. Clegg, *Methods Enzymol.* 1992, **211**, 353-388.
[9] E. Nir, X. Michalet, K. M. Hamadani, T. A. Laurence, D. Neuhauser, Y. Kovchegov, S. Weiss, *J. Phys. Chem. B* 2006, **110**, 22103-22124.

[10] M. Antonik, S. Felekyan, A. Gaiduk, C. A. M. Seidel, *J. Phys. Chem. B* 2006, **110**, 6970-6978.
[11] K. A. Merchant, R. B. Best, J. M. Louis, I. V. Gopich and W. A. Eaton, *Proc. Natl. Acad. Sci. U. S. A.*, 2007, **104**, 1528-1533.

where, $\langle N^{-1} \rangle$ is the mean of the reciprocal number of the donor and acceptor photons and E_m is the measured FRET efficiency. The mean is calculated using the bursts that contribute to the FRET efficiency peak of the labeled DNA. The contribution of shot noise is found to be significantly higher in *BCL-2*-C (pH 7) in the FRET peak at ~0.45. Hence we didn't assign these two peaks as sub-populations of DNA secondary structures.

11.0 smFRET analysis:

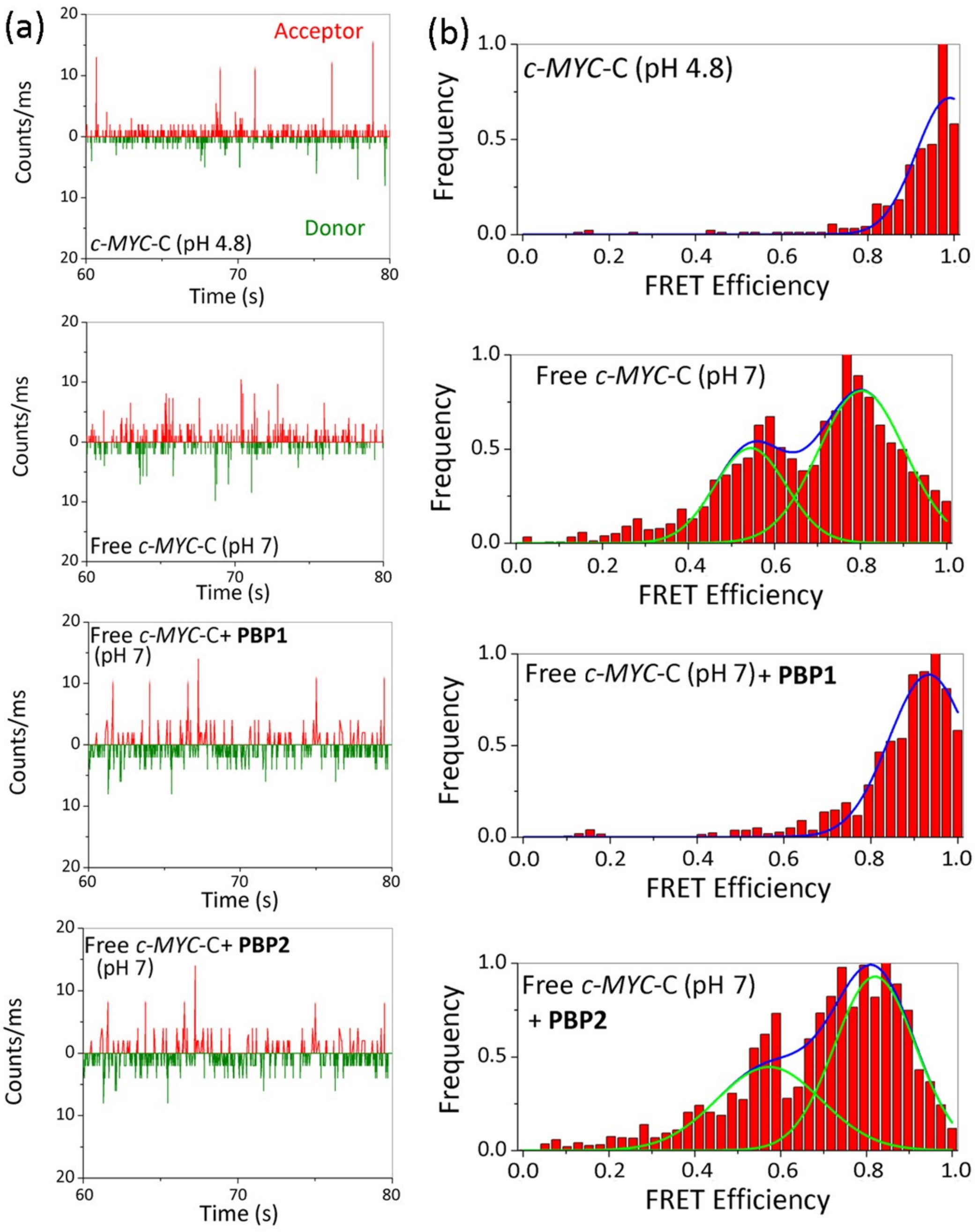

Fig. S8 The smFRET analysis of *c-MYC*-C in the presence and absence of 1 equiv. **PBP1** and **PBP2**. Photon bursts of donor/acceptor (background corrected) (left), and FRET efficiency distributions (right) of 100 pM dual fluorescently labeled free *c-MYC*-C sequence in Milli-Q water (pH adjusted to 7), folded *c-MYC*-C i-motif in 10 mM Na-cacodylate (pH 4.8), in the presence of 1 equiv. **PBP1** (pH adjusted to 7) and in the presence of **PBP2** (pH adjusted to 7).

The fits were determined by best fit method by fitting the histograms into least number of distributions. The histograms were fitted from single Gaussian distribution and then two Gaussian distribution.

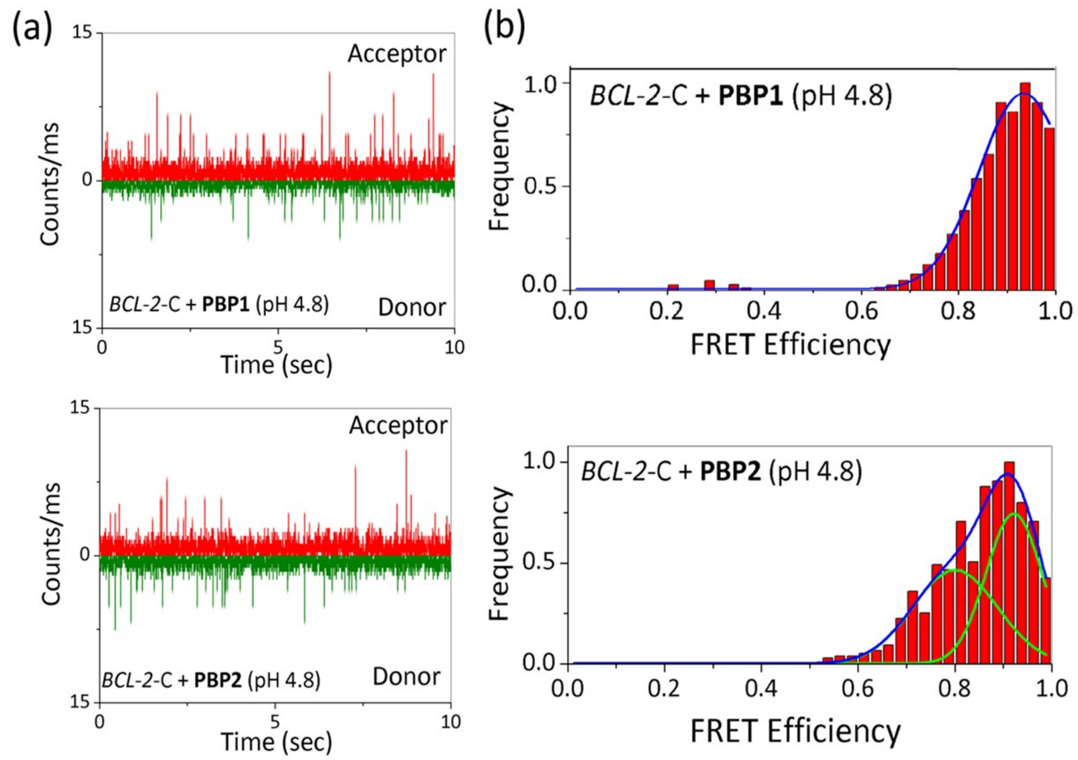

Fig. S9 The smFRET analysis of folded *BCL-2*-C in the presence of 1 equiv. **PBP1** and **PBP2** in 10 mM Na-cacodylate (pH 4.8) buffer. Photon bursts of donor/acceptor (a); and FRET efficiency distributions (b) of 100 pM dual fluorescently labeled *BCL-2*-C in the presence of 1 equiv. **PBP1** or **PBP2** in 10 mM Na-Cacodylate buffer (pH 4.8).

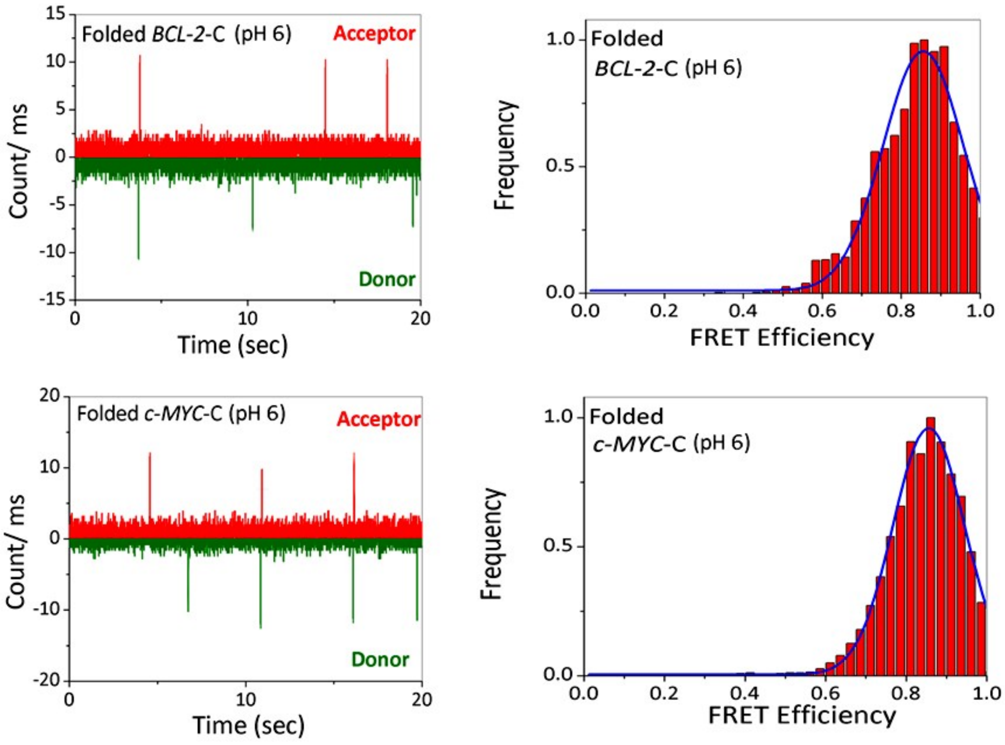

Fig. S10 The smFRET analysis of pre-folded *BCL-2*-C (top) and *c-MYC*-C (bottom) i-motifs (heat annealed in 10 mM Na-cacodylate, pH 4.8) in Milli-Q water, pH adjusted to 6. As seen from the histograms, pre-folded *BCL-2*-C and *c-MYC*-C show high FRET efficiency (~ 88 %) in Milli-Q water, pH 6 and therefore can be considered as stable i-motif structure.

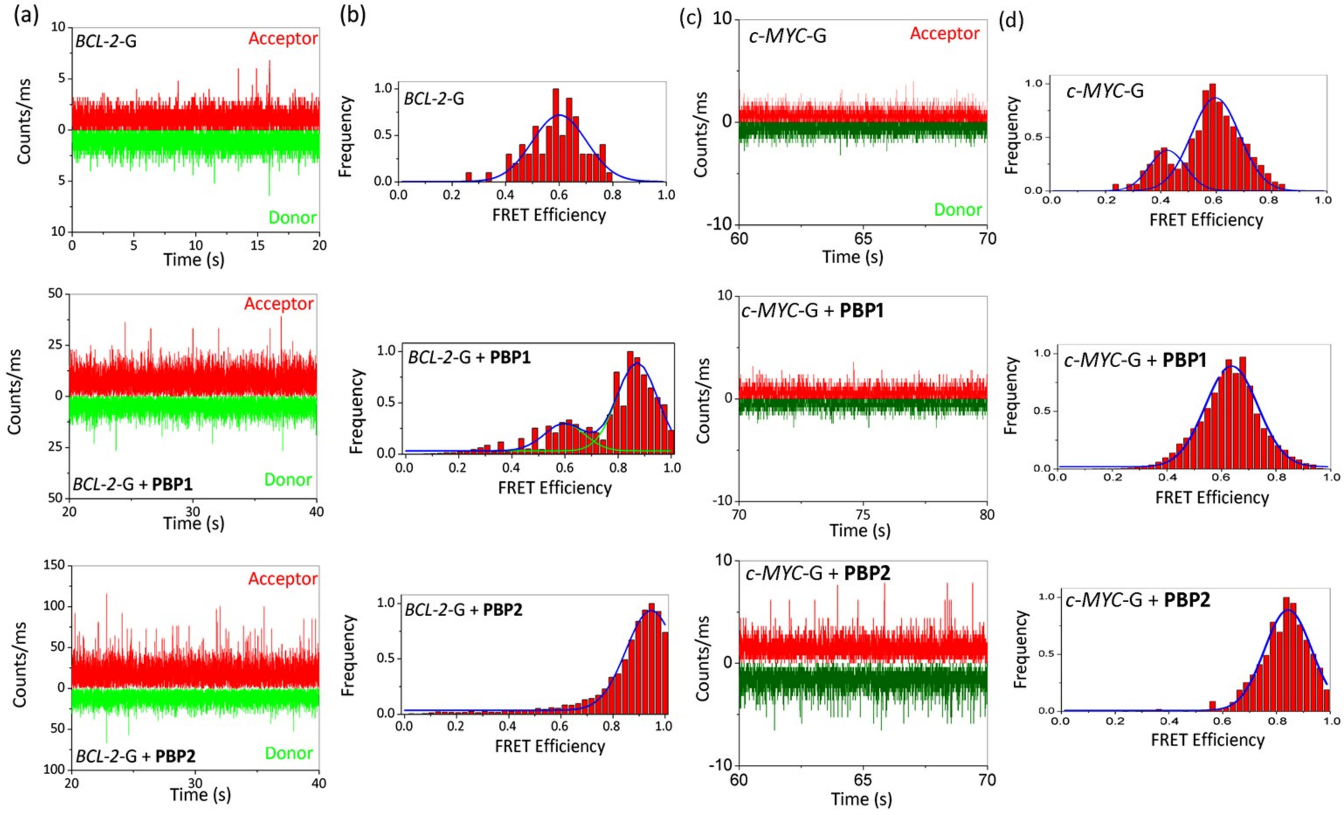

Fig. S11 The smFRET analysis of *BCL-2*-G and *c-MYC*-G G-quadruplex forming sequence in the presence and absence of 1 equiv. **PBP1** and **PBP2**. Photon bursts of donor/acceptor (a) and FRET efficiency distributions (b), of 100 pM dual fluorescently labeled *BCL-2*-G in presence of **PBP1** and **PBP2** in Milli-Q water, pH adjusted to 7. Photon bursts of donor/acceptor (c) and FRET efficiency distributions (d), of 100 pM dual fluorescently labeled *c-MYC*-G in presence of **PBP1** and **PBP2** in Milli-Q water, pH adjusted to 7.

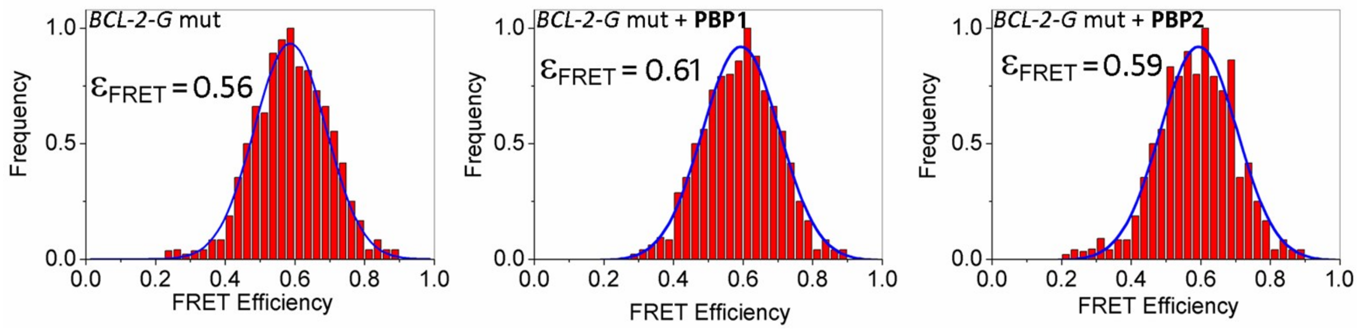

Fig. S12 The smFRET analysis (FRET histograms) of *BCL-2*-G mutant sequence in the presence and absence of 1 equiv. **PBP1** and **PBP2** in Milli-Q water, pH adjusted to 7. *BCL-2*-G-mut: 5′-FAM-d(AG$_2$TGCG$_2$TCGCG$_2$AAG$_2$A$_2$G$_2$ TG$_2$ C GTAA GCG$_2$TGCTG)-TAMRA-3′.

Table S6. The FRET efficiency $\mathbf{E_1}$, $\mathbf{E_2}$ (%) and corresponding donor-acceptor distances (R_{DA}).

System	E_1 (a_1 %)	R_1 (Å)	E_2 (a_2 %)	R_2 (Å)
BCL-2-C (pH 7)	0.45 (9)	57	0.64 (91)	50
BCL-2-C (pH 4.8)	0.95 (100)	33.7	--	--
BCL-2-C (pH 7) + **PBP1**	0.92 (100)	36.6	--	--
BCL-2-C (pH 7) + **PBP2**	0.67 (24)	49	0.95 (76)	33.7
c-MYC-C (pH 7)	0.55 (34)	53.2	0.80 (66)	43.7
c-MYC-C (pH 4.8)	0.95 (100)	33.7	--	
c-MYC-C (pH 7) + **PBP1**	0.93 (100)	35.7	--	
c-MYC-C (pH 7) + **PBP2**	0.57 (38)	52.5	0.82 (62)	42.7
BCL-2-G	0.6 (100)	51.4		
BCL-2-G + **PBP2**	0.95 (100)	33.7		
BCL-2-G + **PBP1**	0.6 (35)	51.4	0.85 (65)	41.2
c-MYC-G	0.45 (35)	57	0.57 (65)	52.5
c-MYC-G + **PBP1**	0.6 (100)	51.4		
c-MYC-G + **PBP2**	0.85 (100)	41.2		

12.0 Lifetime data

Fluorescence Lifetime measurement:

Time-resolved confocal microscopy was employed to measure the fluorescence lifetimes of single labeled (donor only) and dual labeled (donor and acceptor) *BCL-2* and *c-MYC* i-motif and G-quadruplex sequences and their corresponding mutant sequences in the presence of ligands **PBP1** and **PBP2**. Free and folded dual labeled C-rich and G-rich sequences containing stock solutions were prepared as in smFRET. 50 μL of the 100 nM diluted free or folded sample solution was placed on a bare cover slide. The labeled DNA samples were excited through a water immersion objective (magnification 60X and numerical aperture (NA) ≈ 1.2) with a 470 nm pulsed laser (PicoQuant, IRF ~ 100 ps). We used an excitation power of ~ 27 μW and the emission was collected with the same objective and detected by

Micro Photon Devices (MPD-PDM 50CT) through a dichroic beam splitter (HQ490DCXR, Chroma), band pass filter (e.g. XBPA 510, Asahi Spectra), and 30-µm pinhole for spatial filtering to reject out-of-focus signals. The signal was subsequently processed by the PicoHarp-300 time-correlated, single photon counting module (PicoQuant) to generate TCSPC histogram. The parallel ($I_{\|}$, parallel to the polarization of the exciting light) and perpendicular ($I_{\perp}$) components of the fluorescence were separated using a polarizer cube and recorded by using the two detectors (MPDs). $I_{\|}$ and $I_{\perp}$ were combined to generate the fluorescence lifetime decays at magic angle conditions as follows,

$$
\begin{aligned}
I_{magic}(t) &= I_{C}(t)\cos^{2}(54.75°) + I_{\perp}(t)\cdot G\cdot\sin^{2}(54.75°)\\
&= (1/3)\,I_{C}(t) + (2/3)\cdot G\cdot I_{\perp}(t)
\end{aligned}
$$

G is the correction factor for the difference in the detection efficiency of the two detectors. G factor for this microscope setup was measured by tail fitting of fluorescein.[12]

For recording IRF, we used a bare slide and collected the scattered laser light. The FWHM of the IRF for excitation at 470 nm is ~ 100 ps. The fluorescence decay is deconvoluted using the IRF and DAS6 v6.3 software. Intensity decays can be fitted to the single or multi-exponential model as:

$$
I(t) = B + \sum_{i} A_i e^{(-t/\tau_i)} \quad \text{............... (S9)}
$$

Where, A represents the fractional amount of fluorophore in each environment and I(t) is the fluorescence intensity at time t. B is the pre-exponential factor.

The R_{DA} between donor and acceptor labels attached to C-rich and G-rich sequences were estimated from the average FRET efficiency (ε_{avg}) as,

$$
\varepsilon_{avg} = 1 - \frac{\tau_{DA}}{\tau_D} = [1 + (\frac{R_{DA}}{R_0})^6]^{-1} \text{........}(S10)
$$

Where, R_0 represents the Förster distance, the average lifetime (τ_{avg}) of donor labeled C-rich and G-rich sequences are represented as "τ_D" and donor-acceptor labeled C-rich and G-rich sequences are τ_{DA}.

[12] S. T. Hess, E. D. Sheets, A. Wagenknecht-Wiesner and A. A. Heikal, *Biophys. J.*, 2003, **85**, 2566-2580.

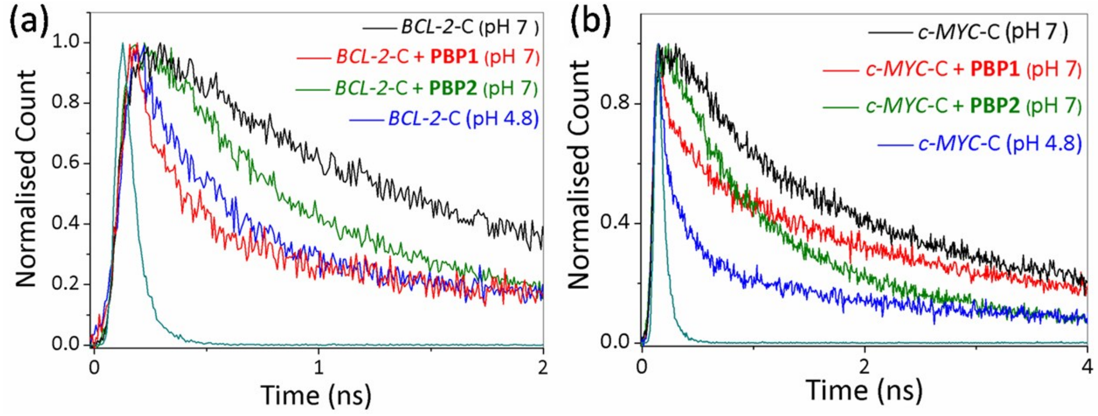

Fig. S13 Fluorescence decay profiles of the (a) donor attached to dual labeled free *BCL-2*-C sequence in Milli-Q water (pH adjusted to 7), free *BCL-2*-C sequence in the presence of 1 equiv. **PBP1** in Milli-Q water (pH adjusted to 7), free *BCL-2*-C sequence in the presence of 1 equiv. **PBP2** in Milli-Q water (pH adjusted to 7), folded *BCL-2*-C i-motif in 10 mM Na-cacodylate (pH 4.8); (b) donor attached to dual labeled free *c-MYC*-C sequence in Milli-Q water (pH adjusted to 7), free *c-MYC*-C sequence in the presence of 1 equiv. **PBP1** in Milli-Q water (pH adjusted to 7), free *c-MYC*-C sequence in the presence of 1 equiv. **PBP2** in Milli-Q water (pH adjusted to 7), folded *c-MYC*-C i-motif in 10 mM Na-cacodylate (pH 4.8) was measured using a time-resolved fluorescence microscope fitted with confocal optics. The cyan line corresponds to the instrument response function.

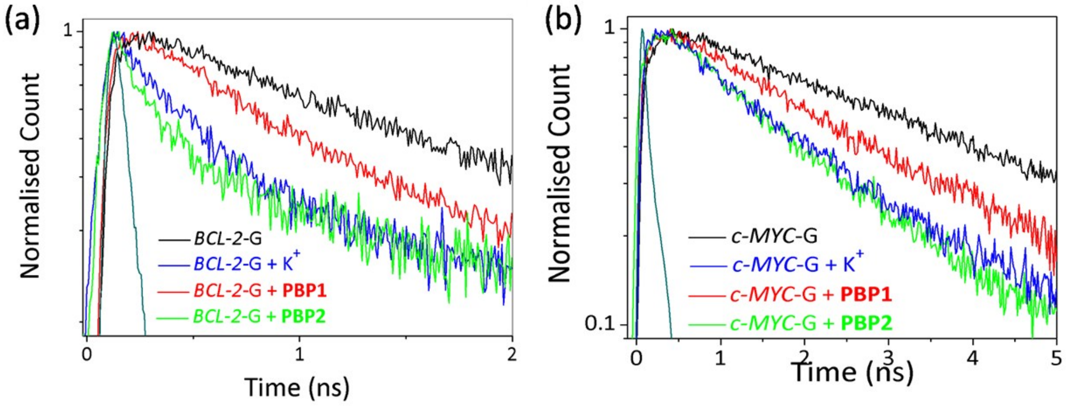

Fig. S14 Fluorescence decay profiles of the (a) donor attached to dual labeled free *BCL-2*-G G-quadruplex forming sequence in Milli-Q water (final pH 7), free *BCL-2*-G sequence in the presence of 1 equiv. **PBP1** in Milli-Q water (final pH 7), free *BCL-2*-G sequence in the presence of 1 equiv. **PBP2** in Milli-Q water (final pH 7), K^+-folded *BCL-2*-G G-quadruplex in Milli-Q water (final pH 7); (b) donor attached to dual labeled free *c-MYC*-G G-quadruplex forming sequence in Milli-Q water (final pH 7), free *BCL-2*-G sequence in the presence of 1 equiv. **PBP1** in Milli-Q water (final pH 7), free *BCL-2*-G sequence in the presence of 1 equiv. **PBP2** in Milli-Q water (final pH 7), K^+-folded *c-MYC*-G in Milli-Q water (final pH 7) was measured using a time-resolved fluorescence microscope fitted with confocal optics. The cyan line corresponds to the instrument response function.

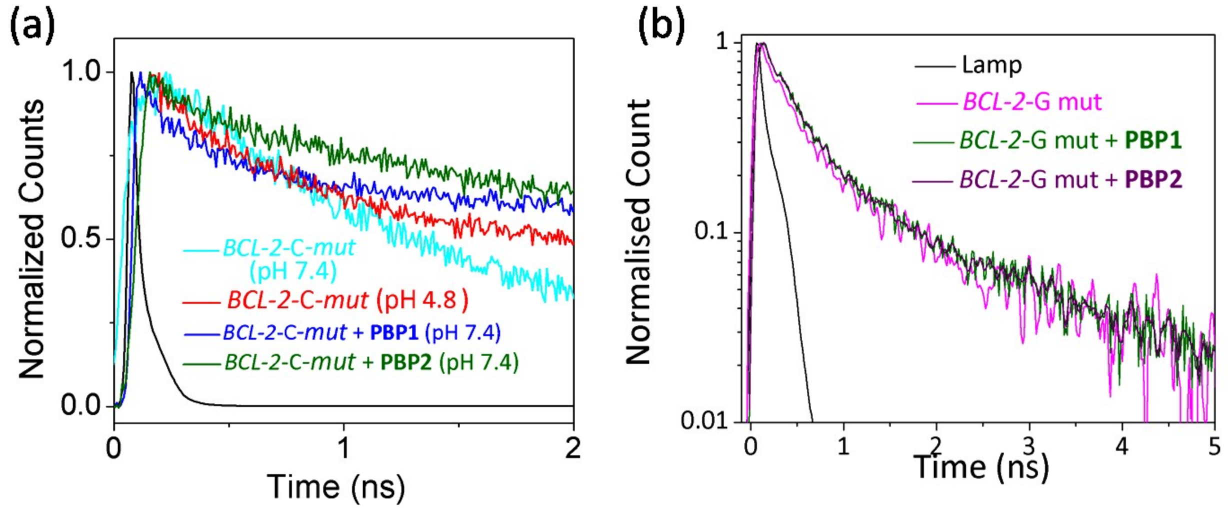

Fig. S15 Fluorescence decay profiles of the (a) donor attached to dual labeled *BCL-2*-C mutant sequence in Milli-Q water (pH 7), *BCL-2*-C mutant sequence in the presence of 1 equiv. **PBP1** in Milli-Q water (pH 7), *BCL-2*-C mutant sequence in the presence of 1 equiv. **PBP2** in Milli-Q water (pH 7), *BCL-2*-C mutant (pH 4.8); (b) donor attached to dual labeled *BCL-2*-G mutant sequence in Milli-Q water (pH 7), *BCL-2*-G mutant in the presence of 1 equiv. **PBP1** in Milli-Q water (pH 7), *BCL-2*-G mutant in the presence of 1 equiv. **PBP2** in Milli-Q water (pH 7), *BCL-2*-G mutant in K^+-buffer (pH 7) was measured using a time-resolved fluorescence microscope fitted with confocal optics. *BCL-2*-G-mut: 5′-FAM d(AG$_2$TGCG$_2$TCGCG$_2$AAG$_2$A$_2$G$_2$TG$_2$CGTAAGCG$_2$TGCTG)-TAMRA-3′; *BCL-2*-C-mut: 5′-FAM d(CAGC$_2$TCGCTC$_2$TGC$_2$TC$_2$T$_2$C$_2$TC$_2$TGCGC$_2$TGC$_2$TCG)-TAMRA-3′.

We have determined the fitting model based on best fit. For instance, we have fitted *c-MYC*-C (pH 4.8) to tri-exponential decay to obtained best fit (residuals) in tri-exponential decay model as shown below,

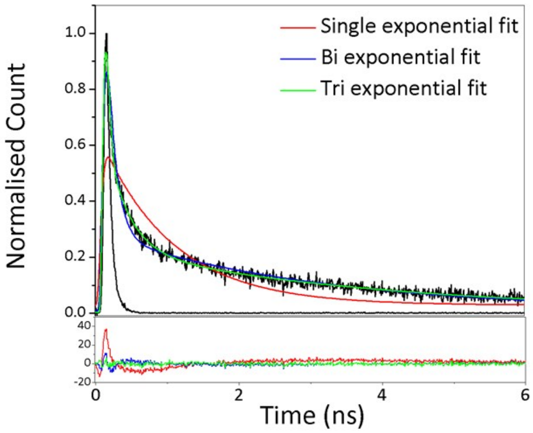

Fig. S16 Representative fit of fluorescence decay profile of *c-MYC*-C in 10 mM Na-cacodylate (pH 4.8) to tri-exponential decay. Lower panel shows the residual of the fitting result.

Table S7. Lifetime parameters of *BCL-2*-C and *c-MYC*-C at λ_{em} = 510 nm.

System		τ_1[a] (A_1)	τ_2[a] (A_2)	τ_3[a] (A_3)	τ_{avg}[a]	ε_{FRET}[a]	R_{DA}[a] (Å)
BCL-2-C (pH 7)	D		2.8 (0.65)	5.6 (0.35)	3.78	0.59	52
	DA	0.12 (0.2)	0.8 (0.3)	2.6 (0.5)	1.56		
BCL-2-mut (pH 7)	D		2.6 (0.7)	6.1 (0.3)	3.62	0.53	54
	DA		0.95 (0.65)	3.1 (0.35)	1.7		
BCL-2-C (pH 4.8)	D		2.8 (0.6)	7.1 (0.4)	4.32	0.81	43.2
	DA	0.12 (0.2)	0.44 (0.5)	2.0 (0.3)	0.84		
BCL-2-C + **PBP1** (pH 7)	D		1.36 (0.67)	7.2 (0.33)	3.24	0.87	40.1
	DA	0.09 (0.63)	0.4 (0.3)	3.5 (0.07)	0.42		
BCL-2-mut + **PBP1** (pH 7)	D		0.86 (0.72)	5.1 (0.28)	2.05	0.55	53
	DA		0.53 (0.75)	2.1 (0.25)	0.92		
BCL-2-C + **PBP2** (pH 7)	D		0.85 (0.6)	4.8 (0.4)	2.43	0.67	48.9
	DA	0.12 (0.16)	0.38 (0.65)	2.78 (0.19)	0.79		
BCL-2-mut + **PBP2** (pH 7)	D		0.83 (0.6)	4.6 (0.4)	2.34	0.59	52
	DA		0.34 (0.7)	2.4 (0.3)	0.96		
c-MYC-C (pH 7)	D		1.4 (0.2)	3.8 (0.8)	3.32	0.52	54.3
	DA	0.16 (0.2)	0.9 (0.5)	3.75 (0.3)	1.6		
c-MYC-C (pH 4.8)	D		2.5 (0.55)	7.2 (0.45)	4.62	0.79	44.1
	DA	0.06 (0.56)	0.47 (0.19)	3.4 (0.25)	0.97		
c-MYC-C + **PBP1** (pH 7)	D		0.8 (0.6)	6.4 (0.4)	3.04	0.72	47
	DA	0.096 (0.35)	0.6 (0.4)	2.4 (0.25)	0.87		
c-MYC-C + **PBP2** (pH 7)	D		0.6 (0.7)	7.6 (0.3)	2.7	0.62	50.7
	DA	0.1 (0.23)	0.5 (0.6)	4.1 (0.17)	1.02		

[a]± 10 % SD

Table S8. Lifetime parameters of *BCL-2*-G and *c-MYC*-G at λ_{em} = 510 nm.

System		τ_1^a (A_1)	τ_2^a (A_2)	τ_3^a (A_3)	τ_{avg}^a	ε_{FRET}^a	R_{DA}^a (Å)
BCL-2-G	D		2.4	-	2.4	0.49	55.36
	DA	0.2 (0.25)	1.59 (0.75)	-	1.24		
BCL-2-G-mut	D		2.8		2.8	0.52	54
	DA	0.17 (0.3)	1.5 (0.4)	2.3 (0.3)	1.34		
BCL-2-G + K^+	D		3.86	-	3.86	0.81	43.2
	DA	0.09 (0.22)	1.22 (0.68)	-	0.74		
BCL-2-G + **PBP1**	D		3.1	-	3.1	0.74	46.2
	DA	0.14 (0.6)	1.8 (0.4)	-	0.8		
BCL-2-G-mut + **PBP1** (pH 7)	D		3.2		3.2	0.52	54
	DA	0.13 (0.3)	1.8 (0.3)	2.4 (0.4)	1.54		
BCL-2-G + **PBP2**	D		4.1	-	4.1	0.86	40.6
	DA	0.1 (0.47)	1.05 (0.53)	-	0.6		
BCL-2-G-mut + **PBP2** (pH 7)	D		3.1		3.1	0.55	53
	DA	0.15 (0.2)	1.1(0.4)	2.3 (0.4)	1.39		
c-MYC-G	D			2.96	2.96	0.5	55
	DA	0.13 (0.22)	0.9 (0.5)	3.6 (0.28)	1.48		
c-MYC-G + K^+	D		2.35 (0.4)	4.6 (0.6)	3.7	0.8	43.6
	DA	0.1 (0.6)	0.47 (0.25)	3.8 (0.15)	0.74		
c-MYC-G + **PBP1**	D		1.0 (0.55)	5.9 (0.45)	3.2	0.69	48.2
	DA	0.12 (0.24)	0.67 (0.5)	2.4 (0.26)	0.99		
c-MYC-G + **PBP2**	D		1.6 (0.6)	7.4 (0.4)	3.9	0.84	41.7
	DA	0.1 (0.6)	0.5 (0.3)	4.1 (0.1)	0.62		
[a]± 10 % SD							

13.0 CD spectroscopy: CD spectra were recorded on a JASCO J-815 spectrophotometer by using a 1 mm path length quartz cuvette. Dual-labeled *c-MYC*-C, *BCL-2*-C and *h-TELO*-C i-motif forming sequences were folded in 10 mM Na-cacodylate buffer (pH 4.8) by heating at 95 ºC for 5 min and subsequent cooling to room temperature to a final concentration of 200 μM. Similarly, dual labeled G-rich sequences *BCL-2*-G and *c-MYC*-G sequences were heat annealed into G-quadruplexes in 60 mM K-cacodylate buffer (pH 7) to a final concentration of 200 μM and diluted in the same buffer. For working solutions, the folded i-motifs are diluted in 10 mM Na-cacodylate buffer (pH 4.8) and free i-motif forming sequences (not heat-annealed) are diluted in Milli-Q water (final pH 7). Ligands **PBP1**, **PBP2** and **PBP3** were added stepwise to free and folded G- and C-rich sequences (pH 7) and the corresponding CD spectra were recorded. CD spectra unlabeled *c-MYC*-C and *BCL-2*-C pre-annealed in 10 mM Na-cacodylate buffer (pH 4.8) were also compared with their labeled counterparts. Binding stoichiometries were measured by continuous variation binding analysis by the method according to Job.[13,14] Ligands **PBP1** and **PBP2** were varied along with the i-motifs or quadruplexes for a fixed and constant summed concentration of 10 μM. The concentrations of **PBP1** and **PBP2** ranging from (0, 2, 4, 6, 8, 1 μM) were added to i-motifs or quadruplexes (10, 8, 6, 4, 2, 0 μM), respectively in 60 mM K-cacodylate buffer of pH 6 or 7 and the CD spectra was recorded. The CD spectra of **PBP1** and **PBP2** was recorded and used

[13] P. Job, *Ann. Chim.*1928, **9**, 113-203.
[14] T. M. Lohman, D. P. Mascotti, *Methods Enzymol.* 1992, **212**, 424-458.

in background correction. The CD spectra represent an average of three scans and were smoothed and zero corrected. Final analysis and manipulation of the data was carried out by using Origin 8.0.

CD spectroscopy was employed to investigate the ligand induced formation of i-motif structures from free *BCL-2*-C and *c-MYC*-C C-rich sequences (Fig. S17-S24, ESI). The CD spectra of folded *BCL-2*-C and *c-MYC*-C i-motifs in 10 mM Na-cacodylate buffer, pH 4.8 or 60 mM K-cacodylate buffer, pH 4.8 exhibited a characteristic positive peak at ~ 286-288 nm and a negative peak at ~ 255-260 nm (Fig. S17, ESI). In contrast, free *BCL-2*-C and *c-MYC*-C sequences in 60 mM K-cacodylate buffer or Milli-Q water (pH adjusted to 7) showed positive peak at ~ 276-279 nm and a negative peak at ~ 250 nm, which is indicative of unfolded DNA in a random coil structure.[15] Additionally, CD spectra of mutated *BCL-2*-C and *c-MYC*-C sequences exhibited a positive peak at ~ 276-279 nm in both pH 4.8 or pH 7, suggesting that they exist in unfolded form (Fig. S19, ESI).

The addition of **PBP1** hardly altered the characteristic CD peaks and intensities of the folded *BCL-2*-C i-motif in 60 mM K-cacodylate buffer, (final pH 4.8) indicating **PBP1** did not induce any significant conformational change to the folded i-motif structure and interacts via an external binding mode[16,17] (Fig. S21a, ESI). Interestingly, a systematic red shift in the positive band to ~ 286 nm was observed when the free *BCL-2*-C sequence was titrated with **PBP1** in 60 mM K-cacodylate buffer, pH 7 (Fig. S22a). This red shift indicates that **PBP1** can induce the formation of folded i-motif structure at neutral pH (pH 7). In contrast, only a slight decrease in ellipticity at ~ 276 nm was observed upon addition of **PBP2** to free *BCL-2*-C and *c-MYC*-C sequences, respectively at 60 mM K-cacodylate buffer, pH 7 (Fig. S22b and S23, ESI). The peak positions of free *BCL-2*-G and *c-MYC*-G remain unchanged in the presence of **PBP1**, but shows an increase in ellipticity upon titration with **PBP2** (Figure S22c, S22d, S23c and S23d, ESI). The CD analysis results are consistent with the smFRET data which suggest that ligand **PBP1** triggers the formation of *BCL-2*-C and *c-MYC*-C i-motif structures and ligand **PBP2** shows weak interactions with the C-rich sequences. In addition, no significant change in CD spectra of *BCL-2* i-motif and G-quadruplexes were observed upon titration with ligand **PBP3** (Fig. S24, ESI).

Next, the binding stoichiometry of these ligands for i-motifs and G-quadruplexes was determined by monitoring the change in CD spectra as a function of the mole fraction of ligands (Job's plot). The slope change between the linear regions suggests a 1:1 binding mode of **PBP1** and **PBP2** for i-motifs and G-quadruplexes, respectively (Fig. S25 and S26, ESI).

[15] H. A. Day, C. Huguin and Z. A. E. Waller, *Chem. Commun.*, 2013, **49**, 7696-7698.
[16] A. Manna and S. Chakravorti, *J. Phys. Chem. B.*, 2012, **116**, 5226-5233.
[17] Y.-M. Chang, C. K.-M. Chen and M.-H. Hou, *Int. J. Mol. Sci.*, 2012, **13**, 3394-3413.

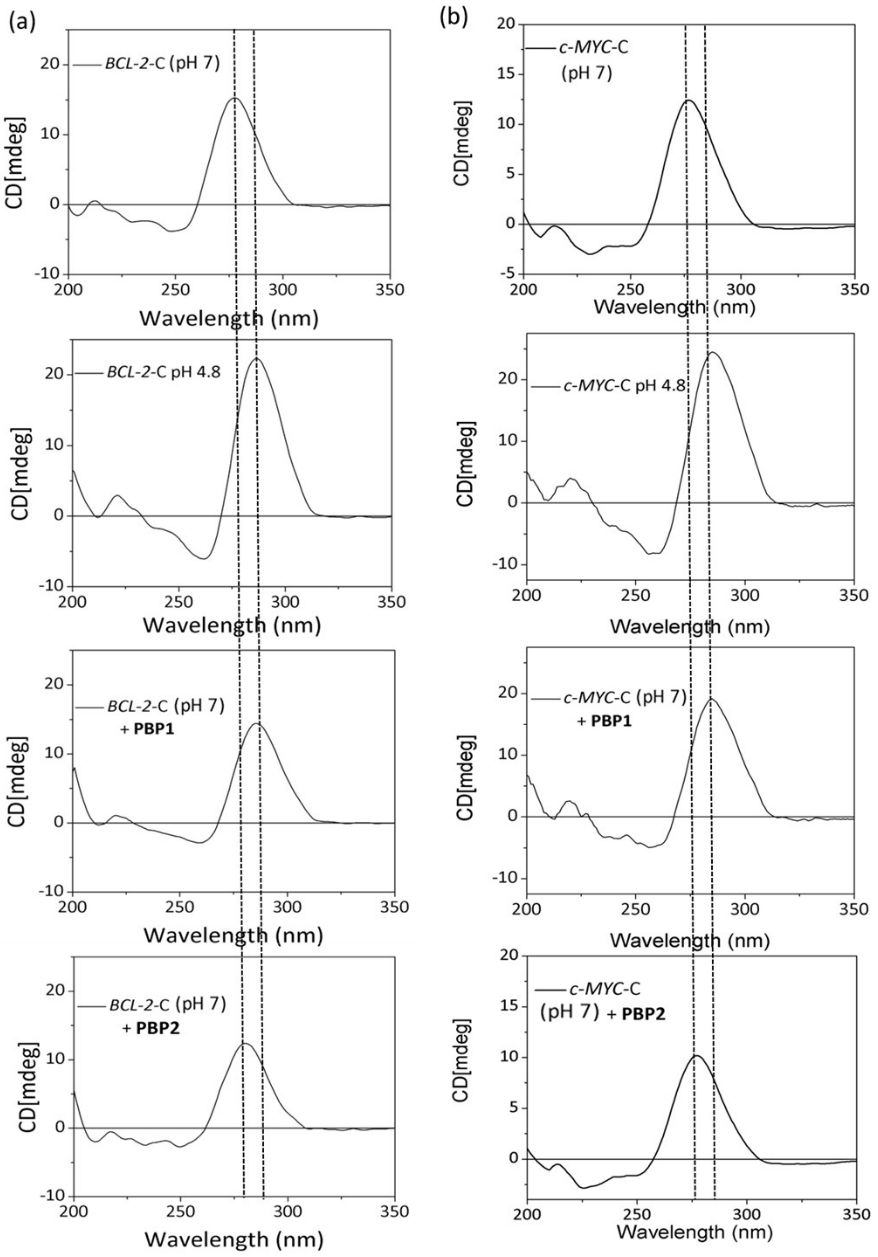

Fig. S17 CD spectra of a solution of (a) free *BCL-2*-C (10 μM) in Milli-Q water (pH 7), folded *BCL-2*-C (10 μM) in Milli-Q water (pH 4.8), free *BCL-2*-C (10 μM) in Milli-Q water (final pH 7) in presence of 1 equiv. **PBP1** (pH 7) and free *BCL-2*-C (10 μM) in Milli-Q water (pH 7) in presence of 1 equiv. **PBP2** (pH 7) (Top to bottom); (b) free *c-MYC*-C (10 μM) in Milli-Q water (pH 7), folded *c-MYC*-C (10 μM) in Milli-Q water (pH 7), free *c-MYC*-C (10 μM) in Milli-Q water (pH 7) in presence of 1 equiv. **PBP1** (pH 7) and free *c-MYC*-C (10 μM) in Milli-Q water (pH 7) in presence of 1 equiv. **PBP2** (pH 7) (Top to bottom); Dotted lines represent the shift in CD peaks from 277 nm to 285 nm.

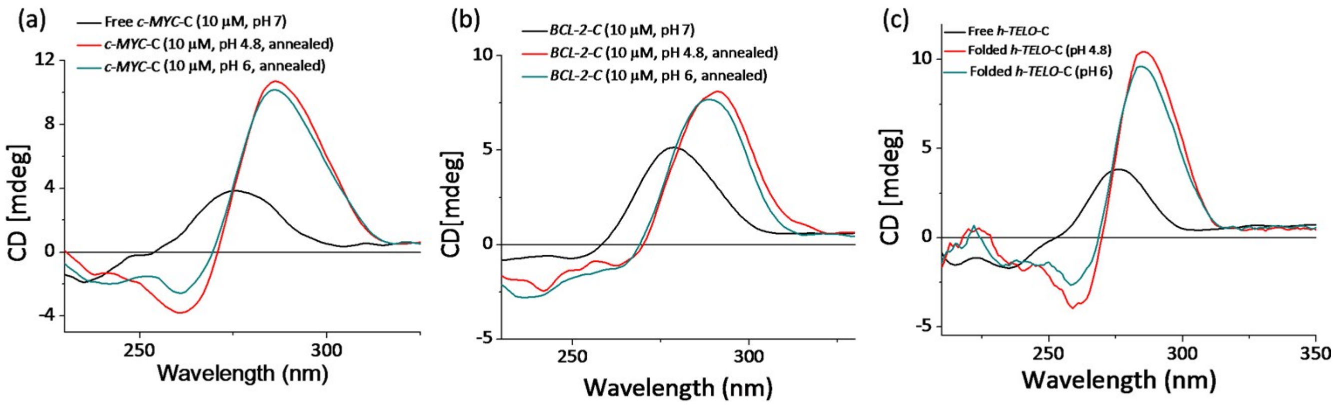

Fig. S18 CD spectra of a solution of (a) free *BCL-2*-C in 60 mM K-cacodylate buffer (pH 7) (black line), folded *BCL-2*-C in 60 mM K-cacodylate buffer (pH 4.8) (red line) and folded *BCL-2*-C in 60 mM K-cacodylate buffer (pH 6) (cyan line); (b) free *c-MYC*-C in 60 mM K-cacodylate buffer (pH 7) (black line), folded *c-MYC*-C in 60 mM K-cacodylate buffer (pH 4.8) (red line) and folded *c-MYC*-C in 60 mM K-cacodylate buffer (pH 6) (cyan line); (c) free *h-TELO*-C in 60 mM K-cacodylate buffer (pH 7) (black line), folded *h-TELO*-C in 60 mM K-cacodylate buffer (pH 4.8) (red line) and folded *h-TELO*-C in 60 mM K-cacodylate buffer (pH 6) (cyan line).

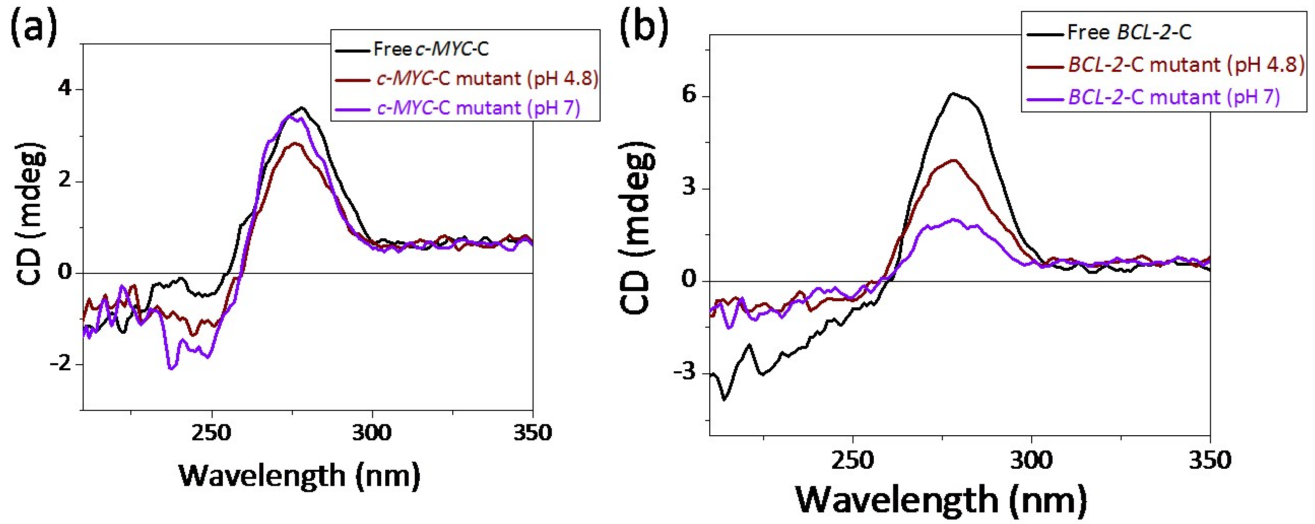

Fig. S19 CD spectra of a solution of (a) free *c-MYC*-C (10 µM) in 60 mM K-cacodylate (pH 7), mutant *c-MYC*-C (10 µM) in 60 mM K-cacodylate (pH 4.8) and mutant *c-MYC*-C (10 µM) in 60 mM K-cacodylate (pH 7); (b) free *BCL-2*-C (10 µM) in 60 mM K-cacodylate (pH 7), mutant *BCL-2*-C (10 µM) in 60 mM K-cacodylate (pH 4.8) and mutant *BCL-2*-C (10 µM) in 60 mM K-cacodylate (pH 7).

BCL-2-C-mut: 5′- FAM-d(CAGC_2TCGCTC_2TGC_2T$C_2$$T_2$$C_2TC_2$TGCG$C_2TGC_2$TCG)-TAMRA-3′;

c-MYC-C-mut: 5′- FAM- d(TC_2TCA$C_2$$T_2CAC_2$ACGCTCT$C_2$ACGCTCT$C_2$A) - TAMRA-3′

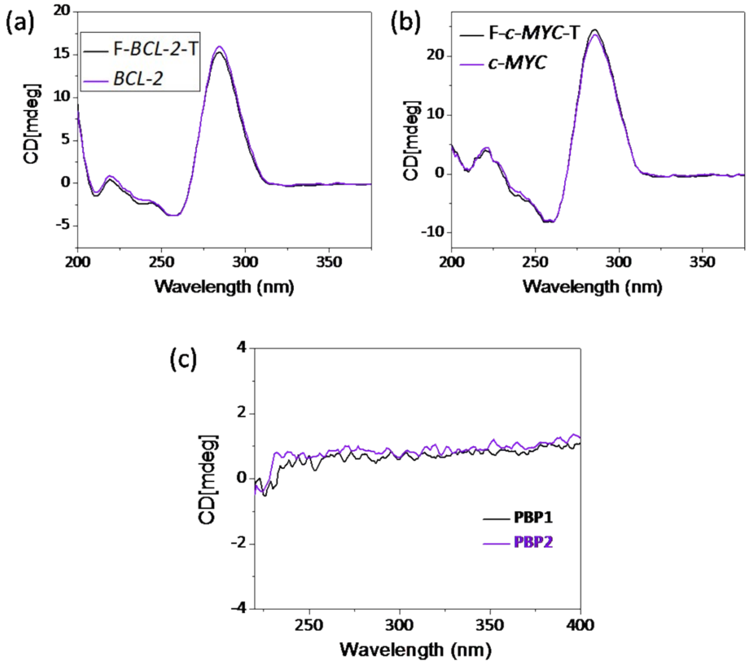

Fig. S20 CD spectra of a solution of (a) FAM-TAMRA dual labeled *BCL-2*-C (10 μM) and unlabeled *BCL-2*-C (10 μM) folded in 10 mM Na-cacodylate buffer (pH 4.8); (b) FAM-TAMRA dual labelled *c-MYC*-C (10 μM) and unlabeled *c-MYC*-C (10 μM) folded in 10 mM Na-cacodylate buffer (pH 4.8); (c) CD spectra of **PBP1** and **PBP2** (30 μM) in 10 mM Na-cacodylate buffer (pH 7) used to discard any contribution of the prolinamide ligands to the CD spectra.

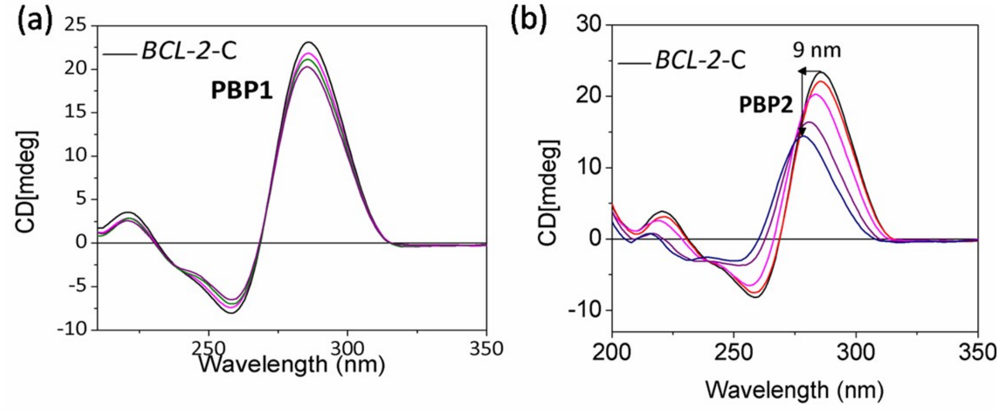

Fig. S21 CD spectra of a solution of (a) folded *BCL-2*-C (10 μM) in 60 mM K-cacodylate buffer (pH 4.8) titrated with **PBP1** (0-1 equiv.), no significant change in CD bands were observed for folded *BCL-2*-C i-motif upon titration with **PBP1**; (b) folded *BCL-2*-C (10 μM) in 60 mM K-cacodylate buffer (final pH 7) titrated with **PBP2** (0-1 equiv.), a systematic blue shift with along with a decrease in ellipticity in the CD band of folded *BCL-2*-C i-motif was observed upon titration with **PBP2**.

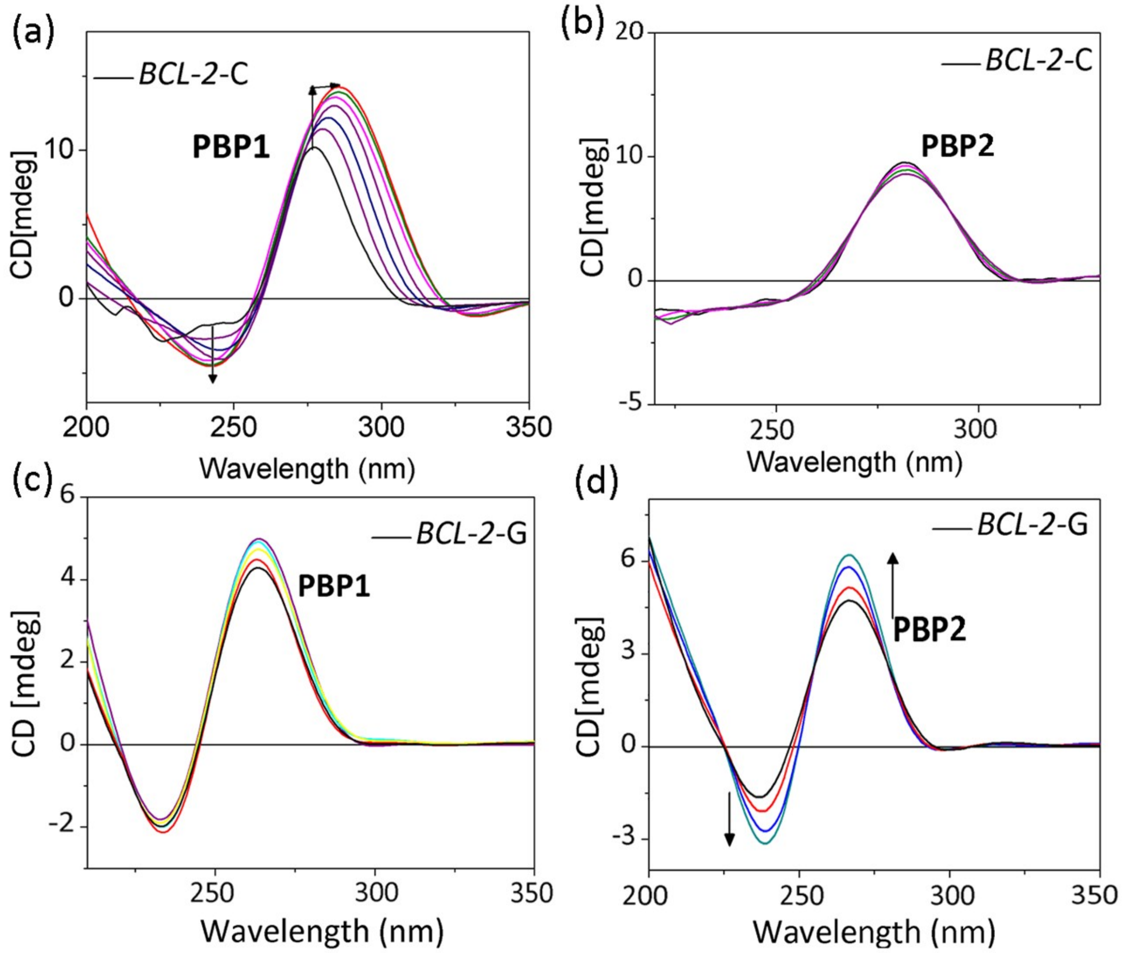

Fig. S22 CD spectra of a solution of free *BCL-2*-C (10 μM) in 60 mM K-cacodylate buffer (pH 7) titrated with (a) **PBP1** (0-1 equiv.); (b) **PBP2** (0-1 equiv.). CD spectra of free *BCL-2*-G (10 μM) in Milli-Q water (pH 7) titrated with (c) **PBP1** (0-1 equiv.); (d) **PBP2** (0-1 equiv.)

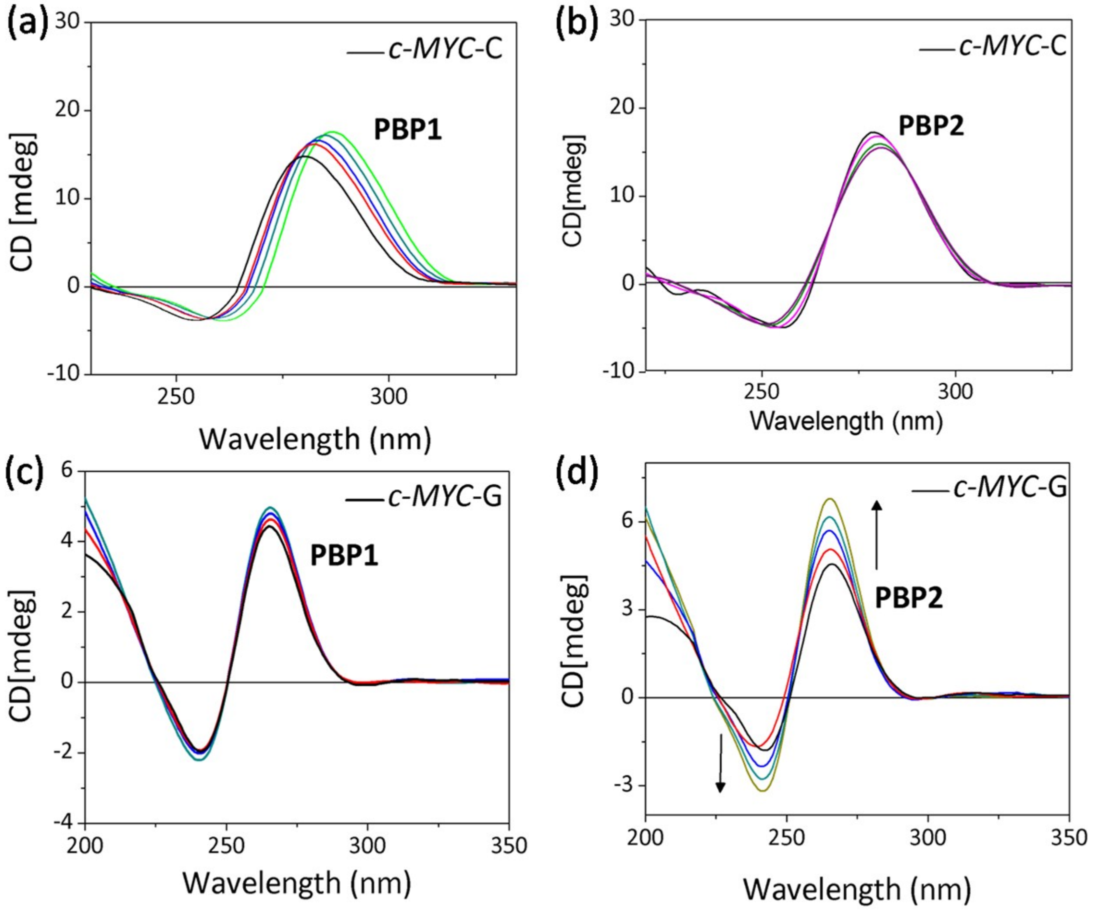

Fig. S23 CD spectra of a solution of free *c-MYC*-C (10 µM) in 60 mM K-cacodylate buffer (pH 7) titrated with (a) **PBP1** (0-1 equiv.); (b) **PBP2** (0-1 equiv.). CD spectra of free *c-MYC*-G (10 µM) in Milli-Q water (pH 7) titrated with (c) **PBP1** (0-1 equiv.); (d) **PBP2** (0-1 equiv.).

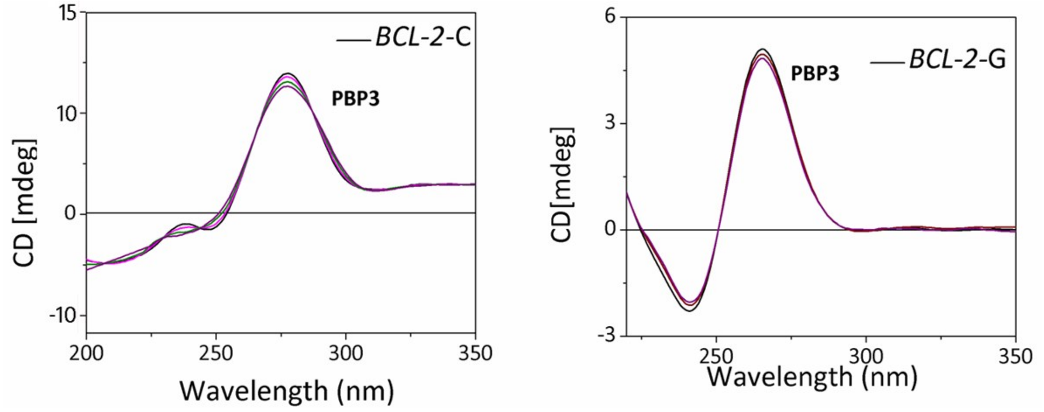

Fig. S24 CD spectra of a solution of free *BCL-2*-C (10 µM) in 60 mM K-cacodylate buffer (pH 7) titrated with **PBP3** (0-1 equiv.) (left). CD spectra of free *BCL-2*-G (10 µM) in Milli-Q water (pH 7) titrated with **PBP3** (0-1 equiv.) (right).

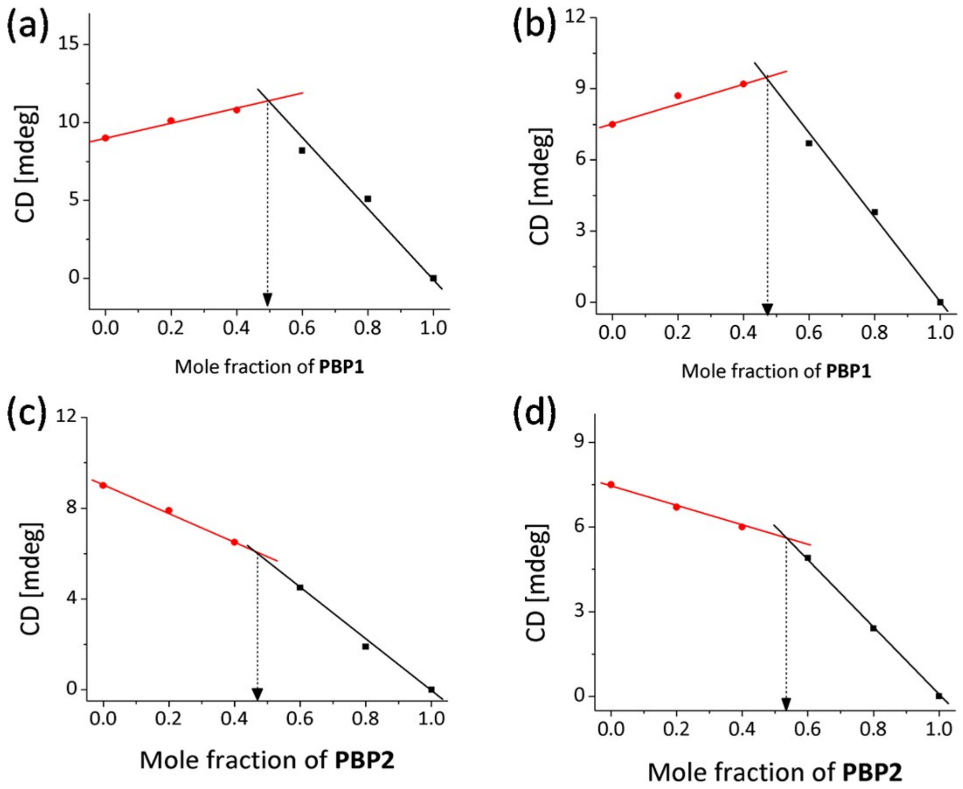

Fig. S25 Job's plot analysis by monitoring continuous variation in the CD ellipticity with concentration of **PBP1** (0, 2, 4, 6, 8, 10 μM) in presence of (a) *BCL-2*-C i-motif (10, 8, 6, 4, 2, 0 μM) in 60 mM K-cacodylate buffer (final pH 6), (b) *c-MYC*-C i-motif (10, 8, 6, 4, 2, 0 μM) in 60 mM K-cacodylate buffer (final pH 6). Job's plot analysis by continuous variation in the CD ellipticity with concentration of **PBP2** (0, 2, 4, 6, 8, 10 μM) in presence of (c) *BCL-2*-G G-quadruplexes (10, 8, 6, 4, 2, 0 μM) in 60 mM K-cacodylate buffer (final pH 7); (d) *c-MYC*-G G-quadruplexes (10, 8, 6, 4, 2, 0 μM) in 60 mM K-cacodylate buffer (final pH 7).

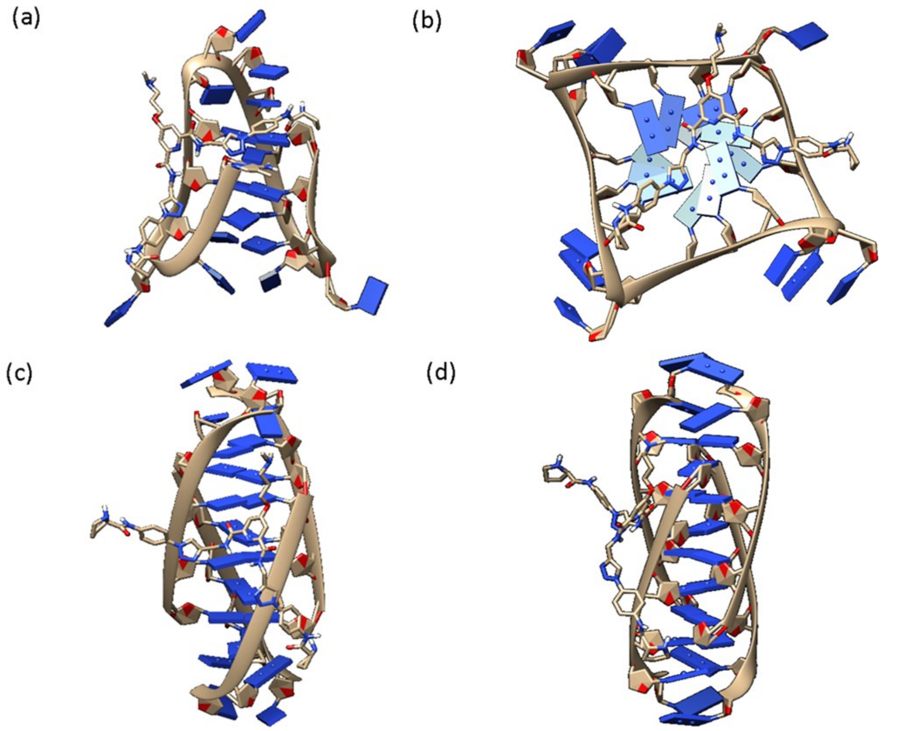

Fig. S26 (a) Schematic representation showing the probable 1:1 binding **PBP1** with a model intramolecular i-motif structure (PDB ID: 1A83); (b) Schematic representation showing the probable 1:1 binding **PBP2** with a model G-quadruplex structure (PDB ID: 1XAV). Schematic representation showing the probable 1:1 binding **PBP1** with a model tetrameric i-motif structure (PDB ID: 1YBL) (c) front view; (d) side view. The models shown here strictly represents the probable binding mode of the ligands to *BCL-2*-C and *c-MYC*-C i-motifs based on fluorescence and CD spectroscopy data. Till date the solution or X-ray crystallographic structures of *BCL-2*-C and *c-MYC*-C i-motifs are not known in literature and hence these i-motif PDB structures cannot fully represent *BCL-2*-C and *c-MYC*-C i-motifs. Therefore, we relied on fluorescence and CD spectroscopy data for the investigation of the binding mode.

14.0 MTT assay[18]

Human breast adenocarcinoma (MCF-7), human colon cancer cells (HCT116) and normal mouse myoblast (C2C12) cells were cultured in DMEM containing high glucose (5.5 mM) supplemented with 10 % FBS at pH 7.4. Cells were maintained in tissue culture plates containing 4×10^5 cells/well at 37 °C in an atmosphere of 5 % carbon dioxide (CO_2)/95 % air. The MTT cell proliferation assay determines the ability of living cells to reduce the yellow tetrazole, 3-(4,5-dimethylthiazol-2-yl)-2,5-diphenyltetrazolium bromide (MTT) to purple formazan crystals by mitochondrial enzymes. All the cell lines were treated with various concentrations of prolinamide derivatives **PBP1** and **2** for 24 h and 48 h. Following incubation with each compound, 20 µL of

[18] T. Mosmann, *J Immunol Methods*, 1983, **65**, 55-63.

MTT was added (at a concentration of solution 5 mg/mL in phosphate-buffered saline, pH 7.4) to each well. After incubation for 4 h at 37 °C, the culture medium was removed, and the formazan crystals were dissolved in 200 µL DMSO. Absorbance (A) of formazan dye was measured at 570 nm using a microplate reader.The background absorbance was determined at 690 nm and subtracted from the 570 nm measurement.

The percentage of viable cells was determined by the equation (S11):

$$Viable\ cells\ (\%) = \frac{A\ of\ treated\ cells}{A\ of\ untreated\ cells} \times 100 \ldots\ldots.(S11)$$

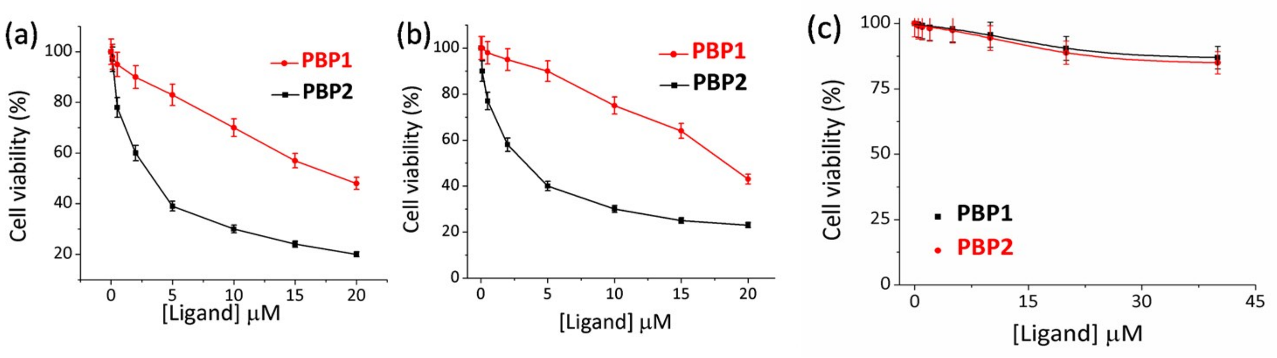

Fig. S27 Representative plots for the cell viability (MTT assay) in (a) MCF-7; (b) HCT116; and (c) C2C12 cells with increasing concentration of **PBP1** (0-10 µM) and **PBP2** (0-15 µM) for 24 h.

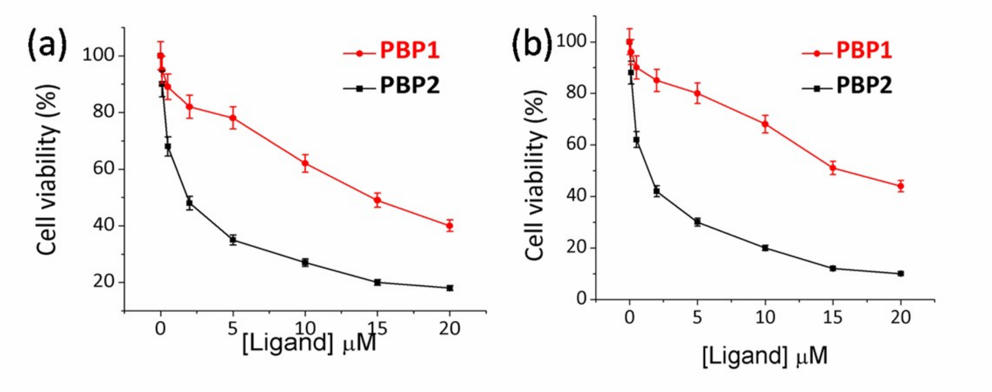

Fig. S28 Representative plots for the cell viability (MTT assay) in (a) MCF-7 and (b) HCT116 cells upon treatment with increasing concentration of **PBP1** (0-10 µM) and **PBP2** (0-15 µM) for 48 h**.**

Table S9. Table representing the concentration for 50 % cell growth inhibition (IC_{50}) of **PBP1** and **PBP2** (after 24 h).

Compounds	IC_{50} (μM)		
	**MCF7**	**HCT116**	**C2C12**
**PBP1**	17.9 ± 1.8	18.5 ± 1.9	>40
**PBP2**	3.3 ± 0.7	3.9 ± 0.9	>40

Table S10. Table representing the concentration for 50 % cell growth inhibition (IC_{50}) of **PBP1** and **PBP2** (after 48 h).

Compounds	IC_{50} (μM)		
	**MCF7**	**HCT116**	**C2C12**
**PBP1**	14.4 ± 1.4	15.1 ± 1.5	>40
**PBP2**	1.7 ± 0.2	1.3 ± 0.15	>40

15.0 qRT-PCR:

In order to evaluate the effect of bis-prolinamide derivatives (**PBP1** and **PBP2**) at the transcriptional level of *BCL-2* gene, qRT-PCR was performed. Ligands **PBP1** and **PBP2** were added at IC_{50} dose (24 h) to human breast adenocarcinoma (MCF-7) (17.9 μM and 3.3 μM) and human colon cancer cells (HCT116) (18.5 μM and 3.9 μM) and incubated in a humidified atmosphere with 5 % CO_2 in air at 37 °C. After 24 h, total RNA was extracted from ligand treated and untreated cells by using the Trizol kit according to the manufacturer's protocol (Invitrogen Corporation). Next, cDNA library was prepared by High Capacity cDNA Reverse Transcription Kit (Applied Biosystems). The Master mix (2X) was prepared by mixing 2 μL 10X RT buffer, 0.8 μL 25X 100 mM dNTP mix, 2 μL 10X RT random primer, 1 μL of reverse transcriptase, 1 μL of RNase inhibitor and 3.2 μL nuclease free water. Then, 10 μL sample was added to 10 μL 2X master mix and reverse transcription was performed on a 96 well Thermal Cycler (Veriti, Applied Biosystems). The thermal cycling condition was programmed as 10 min at 25 °C, 120 min at 37 °C and 5 min at 85 °C for one single cycle. Using the cDNA library as template, we carried out quantitative Real-time polymerase chain reaction (qRT-PCR) on a Light Cycler 480 II (Roche) with SYBR green JumpStart TaqReadyMix (Sigma, Saint Louis, USA) reagent. The primer sequences used for the qRT-PCR analyses are as follows:[19]

[19] X.-D. Wang, T.-M. Ou, Y.-J. Lu, Z. Li, Z. Xu, C. Xi, J.-H. Tan, S.-L. Huang, L.-K. An, D. Li, L.-Q. Gu and Z.-S. Huang, *J. Med. Chem.*, 2010, **53**, 4390-4398.

BCL-2 (forward):5′-GAGGATTGTGGCCTTCTTTG-3′

BCL-2 (reverse): 5′-GCCGGTTCAGGTACTCAGTC-3′

GAPDH (forward): 5'-GACGGCCGCATCTTCTTGT-3'

GAPDH (reverse): 5'-CACACCGACCTTCACCATTTT-3'

18S rRNA (forward): 5'-GATTCCGTGGGTGGTGGTGC-3'

18S rRNA (reverse): 5'-AAGAAGTTGGGGGACGCCGA-3'

18S rRNA (NCBI Reference Sequence: NR_003278.1)

The PCR mixture (25 μL) contained 15 pmol of each primer, 7 μL of water, 5 μL of cDNA, 12.5 μL 2X JumpStart Taq ReadyMix. The PCR mixture samples were sealed in 96-well plates (Roche) and PCR amplification was performed using Light Cycler 480 II real-time PCR detection system (Roche). The thermal cycling conditions were 2 min at 94 °C and then 40 cycles of 15 s at 94 °C, followed 60 s at 60 °C. For relative quantification of gene expression, the comparative cycle threshold method (C_T method) was used.[20] The C_T for target and housekeeping standard (GAPDH and 18S rRNA) were determined for **PBP1** and **PBP2** treated or untreated samples. The arithmetic calibrator ($2^{-\Delta\Delta CT}$) was used to calculate the relative mRNA level expression of *BCL-2* in **PBP1** and **PBP2** treated or untreated cells. The difference in *BCL-2* expression was presented as fold changes. This experiment was performed in triplicate to confirm the reproducibility of the results.

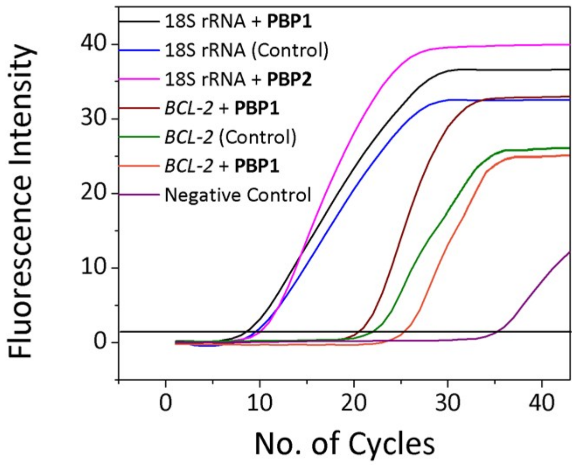

[20] F. Leite, S. O'Brien, M. J. Sylte, T. Page, D. Atapattu and C. J. Czuprynski, *Infect. Immun.*, 2002, **70**, 4336-4343.

Fig. S29 Raw curves for RNA quantification showing C_t values. Here 18S rRNA was used as control gene to quantify the level of *BCL-2* expression. Ligands **PBP1** and **PBP2** were treated at their 24 h IC_{50} doses in HCT116 cells.

Table S11. Table representing the C_t values for RNA quantification upon treatment of HCT116 cells with **PBP1** (18S rRNA Control).

***BCL-2* (Experimental) $C_t(1)$**	**18S rRNA (Experimental) $C_t(2)$**	***BCL-2* (Control) $C_t(3)$**	**18S rRNA (Control) $C_t(4)$**	**$\Delta C_t(1) = [C_t(1) - C_t(2)]$**	**$\Delta C_t(2) = [C_t(3) - C_t(4)]$**
20.6	8.8	22.0	9.6	11.8	12.4

$\Delta\Delta C_t = \Delta C_t(1) - \Delta C_t(2) = 11.8 – 12.4 = -0.6$.

$2^{-\Delta\Delta Ct}$ (**PBP1**) = 1.5 fold.

Table S12. Table representing the C_t values for RNA quantification upon treatment of HCT116 cells with **PBP1** (GAPDH Control).

***BCL-2* (Experimental) $C_t(1)$**	**GAPDH (Experimental) $C_t(2)$**	***BCL-2* (Control) $C_t(3)$**	**GAPDH (Control) $C_t(4)$**	**$\Delta C_t(1) = [C_t(1) - C_t(2)]$**	**$\Delta C_t(2) = [C_t(3) - C_t(4)]$**
20.3	19.1	21.4	19.7	1.2	1.7

$\Delta\Delta C_t = \Delta C_t(1) - \Delta C_t(2) = 1.2 – 1.7 = -0.5$

$2^{-\Delta\Delta Ct}$ (**PBP2**) = 0.35 fold

Table S13. Table representing the C_t values for RNA quantification upon treatment of HCT116 cells with **PBP2** (18S rRNA control).

***BCL-2* (Experimental) $C_t(1)$**	**18S rRNA (Experimental) $C_t(2)$**	***BCL-2* (Control) $C_t(3)$**	**18S rRNA (Control) $C_t(4)$**	**$\Delta C_t(1) = [C_t(1) - C_t(2)]$**	**$\Delta C_t(2) = [C_t(3) - C_t(4)]$**
25.5	10.2	22.0	9.6	15.3	12.4

$\Delta\Delta C_t = \Delta C_t(1) - \Delta C_t(2) = 15.3 – 12.4 = 2.9$

$2^{-\Delta\Delta Ct}$ (**PBP2**) = 0.13 fold

Table S14. Table representing the C_t values for RNA quantification upon treatment of HCT116 cells with **PBP2** (GAPDH control).

BCL-2 **(Experimental)** **$C_t(1)$**	**GAPDH (Experimental)** **$C_t(2)$**	***BCL-2*** **(Control)** **$C_t(3)$**	**GAPDH (Control)** **$C_t(4)$**	**$\Delta C_t(1)$ = $[C_t(1) - C_t(2)]$**	**$\Delta C_t(2)$ = $[C_t(3) - C_t(4)]$**
24.0	20.2	21.4	19.7	3.8	1.7

$\Delta\Delta C_t = \Delta C_t(1) - \Delta C_t(2) = 3.8 – 1.7 = 2.2$

$2^{-\Delta\Delta Ct}$ (**PBP2**) = 0.24 fold

16.0 Western Blot

Human breast adenocarcinoma (MCF-7) and human colon cancer cells (HCT116) were cultured in DMEM (high glucose), supplemented with L-glutamine and 10 % FBS. Ligands **PBP1** and **PBP2** were added at IC_{50} dose (24 h) to MCF-7 (17.9 µM and 3.3 µM) and HCT116 (18.5 µM and 3.9 µM) cells and incubated at 37 °C in 5 % CO_2 in air for 24 h. After incubation, cells were washed once with PBS (pH 7.4) and lysed with cold cell lyses buffer (20 mM Tris, 100 mM NaCl, 1 mM EDTA in 0.5 % Triton X-100). Cell lysates were collected from the treated and untreated cells, and the total protein content in each sample was estimated by Lowry method.[21] Equal amount of proteins (60 µg) from the cell lysates were loaded and separated by 10 % SDS–PAGE and then transferred to nitrocellulose membranes by western blotting. The membranes were blocked with BSA, washed and probed using primary antibodies directed against BCL-2 and GAPDH (as loading control) for overnight at room temperature. The blots were washed and immunoreactive bands were incubated with a 1:10000 dilution of Anti-rabbit IgG, HRP-linked secondary antibody for 2 h at room temperature. Binding signals were visualized with TMB substrate. Relative band intensities were determined by densitometric method using ImageJ software. Western blot experiments were performed in triplicate to confirm the results

[21] O. H. Lowry, N. J. Rosebrough, A. L. Farr and R. J. Randall, *J. Biol. Chem.*, 1951, **193**, 265-275.

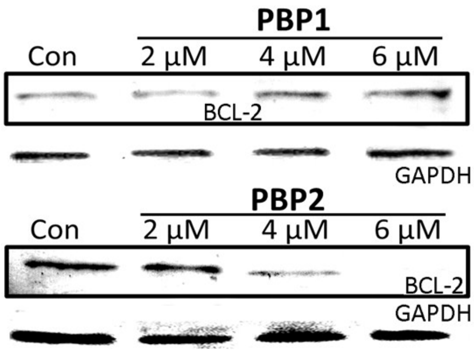

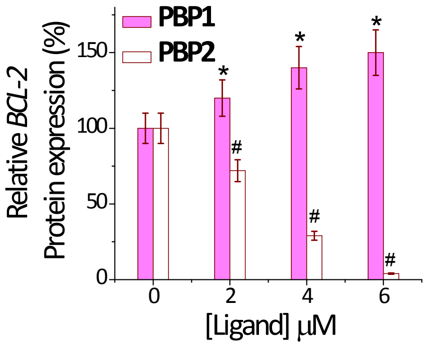

Fig. S30 Immunoreactive bands of BCL-2 protein analyzed by Western blot in HCT116 cells upon treatment with increasing concentrations of **PBP1** and **PBP2** (Left). Densitometric analysis of the immunoblots showing the change in *BCL-2* expression upon increase in concentrations of **PBP1** and **PBP2** (Right). The blots show that **PBP2** exhibits a dose dependent decrease in *BCL-2* protein expression. The blots show that **PBP1** shows a steady increase in *BCL-2* protein expression. Data shown here as mean ± SD. *P < 0.05, #P < 0.01, versus control.

17.0 Dual luciferase reporter assay:

HCT116 cells were seeded in 6-well plates in DMEM (10 % FBS) and incubated in 5 % CO_2 in air at 37 °C for overnight (16 h). The transfection of HCT116 cells was carried out using Lipofectamine 2000. For each well, 0.3 µg of LB322 *BCL-2* promoter containing firefly plasmid and 30 ng of pRL-TK (Renilla) in the ratio of 10:1 (LB322:pRL-TK) and Lipofectamine 2000 reagent (1 µL, Invitrogen) were diluted in DMEM (without FBS) media (Invitrogen) (plasmid mixture in 0.5 mL and Lipofectamine in 0.5 mL DMEM) and kept in room temperature for 5 min. 1:1 (v/v) solutions of DNA and Lipofectamine 2000 were mixed and incubated at room temperature for 40 min for complexation, as per the manufacturer's protocol. The DNA:Lipofectamine complexes were gently added to ~ 50 % confluent cells grown in 6-well plates and the cells were incubated at 37 °C in 5 % CO_2 in air for 6 h. After that the media was drained off and the wells were replenished with 2 mL DMEM (10 % FBS) containing 5 µM of **PBP1** or **PBP2** and incubated at 37 °C in 5 % CO_2 in air for 40 h. After 40 h incubation, cells were washed with PBS, lysed and the levels of firefly and Renilla luciferase luminescence were measured using the Promega Dual Luciferase assay system according to the manufacturer's protocol using Promega GloMax luminometer. Ratios of firefly signal:Renilla signal (FF/RL) were calculated and normalized to ensure equal transfection.

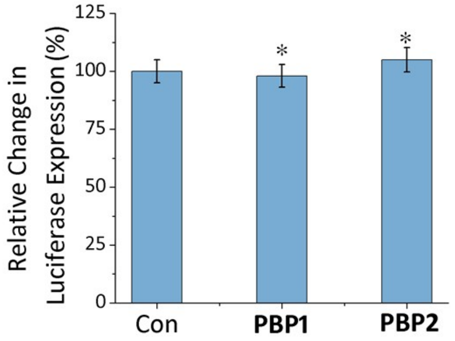

Fig. S31 Relative change in luciferase expression (Firefly/Renilla in %) in pBV-Luc plasmid containing non G-quadruplex or non i-motif forming sequence in promoter region upon treatment with 5 µM of **PBP1** or **PBP2**. pBV-Luc was co-transfected with pRL-TK in HCT116 cells and the firefly expression was normalized with respect to Renilla expression. Data shown here as mean ± SD. *P < 0.05, #P < 0.01, versus untreated control cells.

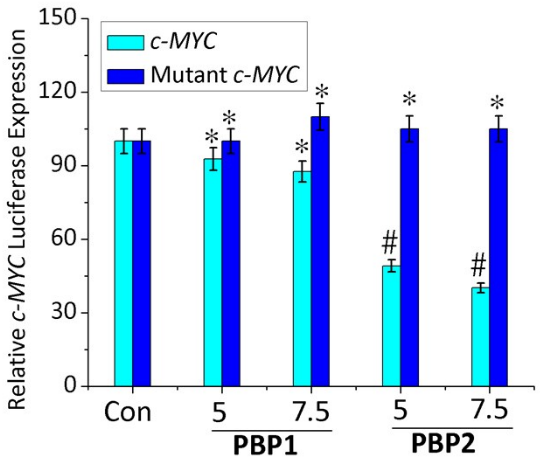

Fig. S32 Relative luciferase expression in Del4 plasmid containing *c-MYC* promoter compared to Del4 plasmid containing mutated *c-MYC* promoter upon treatment of 5 and 7.5 µM of **PBP1** and **PBP2** in HCT116 cells. Del4 plasmid obtained from the lab of Prof. Bert Vogelstein. Data shown here as mean ± SD. *P < 0.05, #P < 0.01, versus untreated control cells. These results indicate that **PBP1** preferentially targets *BCL-2* promoter in cellular system and regulate gene expression.

18.0 Flow cytometric assay of apoptosis:

The percentages of cells undergoing apoptosis were determined by flow cytometry using Annexin V–FITC and propidium iodide (PI) dual staining. The apoptosis assay was conducted as described in the manufacturer's protocol (Invitrogen). To observe the anti-apoptotic properties, first apoptotic cells were

prepared by serum starvation.[22] Briefly, 1 × 10^6 MCF-7 and HCT116 cells were cultured in 6-well plates using DMEM supplemented with 10 % FBS at pH 7.4. The growth media was drained off after the cells reached ~ 50 % confluency, and then the cells were washed three times with PBS. Then the cells were kept in DMEM without any FBS and incubated at 37 °C in 5 % CO_2 in air for 48 h for serum starvation. After 48 h, fresh growth media supplemented with 10 % FBS at pH 7.4 was added to the cells, which were then treated with **PBP1** (5 μM) and **PBP2** (5 μM), respectively for 24 h. Cells were then harvested with trypsinization. After centrifugation at 800 rpm for 5 min at 4 °C, cell pellet was suspended in 500 μL 1× binding buffer and then treated with 5 μL Annexin V–FITC and 2 μL PI. After incubation for 5 min on ice, each sample was analysed immediately using fluorescence–activated cell sorting (FACS) analysis (BD Biosciences, Mountain View, CA, USA). Approximately 10,000 cells were detected for each sample. Cytogram analysis was done using the Cell Quest software. The experiment was performed in triplicate to confirm the results.

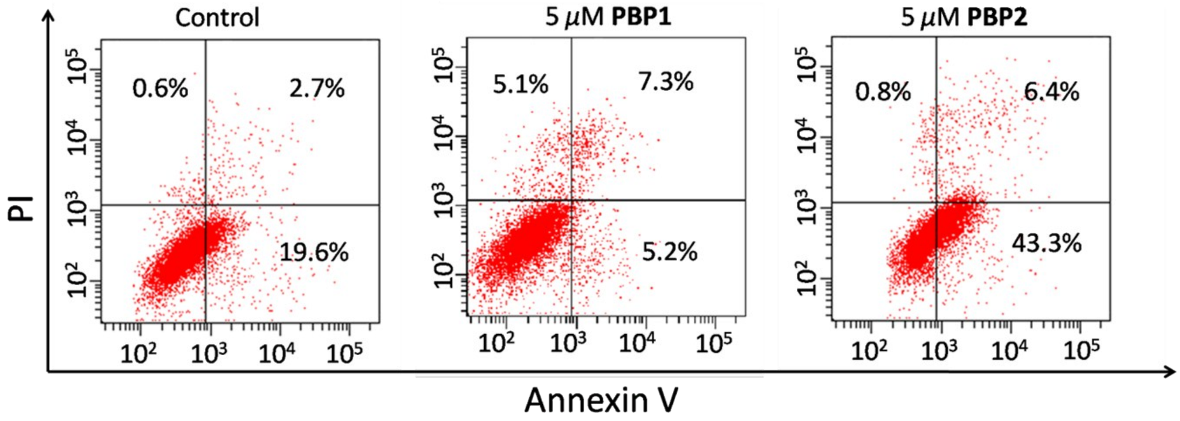

Fig. S33 Apoptosis experiment with HCT116 cancer cell line. The data shows similar anti-apoptotic and apoptotic properties of **PBP1** (5 μM) and **PBP2** (5 μM), when treated in 48 h serum starved cells as observed in Fig. 4e.

19.0 Molecular modeling

Molecular docking studies were performed to generate Fig. S26 using the Auto-Dock 4.0 program[23]. Protein Data Bank files PDB ID: 1XAV[24] was considered as model *c-MYC* quadruplex and PDB ID:

[22] A. J. Raffo, H. Perlman, M.-W. Chen, M. L. Day, J. S. Streitman and R. Buttyan, *Cancer Res.*, 1995, **55**, 4438-4445.
[23] G. M. Morris, D. S. Goodsell, R. S.Halliday, R. Huey, W. E. Hart, R. K. Belew, A. J. Olson, *J. Comput. Chem.,* 1998, **19**, 1639-1662.
[24] A. Ambrus, D. Chen, J. Dai, R. A. Jones, D. Yang, *Biochemistry* 2005, **44**, 2048-2058.

1A83 and 1YBL were considered as model i-motif DNA, respectively for docking studies. Gaussian 03 program,[25] B3LYP/6-31+G(d) basis set[26] used for the DFT analysis of **PBP1** and **PBP2** to obtain the energy minimized structures. Next Lamarckian genetic algorithm (LGA) with the default parameters from AutoDock 4.0 program was employed for the docking calculations of the energy minimized structures of **PBP1** and **PBP2**. Docking was performed without water and counter ions. A maximum of 25 million energy evaluations was applied for the experiment. The results were clustered using a tolerance of 2.0 Å and 10 lowest potential energy structures were collected from the experiment. Chimera 1.6.2 software was used to image docked DNA:ligand complex structure.

20.0 Caspase 3/7 assay

The level of active caspases 3 and 7 was monitored by flow cytometry using FLICA based assay as per the manufacturer's protocol (Vybrant FAM caspase 3/7 assay kit, Invitrogen).[27] Serum starved HCT116 cells were similarly prepared as in Annexin V–FITC and propidium iodide (PI) dual staining assay. Next, the cells were replenished with fresh growth media (with 10 % FBS) at pH 7.4 and treated with **PBP1** and **PBP2** (5 μM), respectively for 24 h. Cells were then harvested by trypsinization and resuspended in culture media to a concentration of 10^6 cells/mL. 300 μL of the cell suspension was then transferred to flow tubes and 10 μL of 30X FLICA reagent was added to each tube. The flow tubes were then incubated at 37 °C in 5 % CO_2 in air for 1 h with occasional stirring. The cells were then washed thrice and analyzed using fluorescence–activated cell sorting (FACS) analysis (BD Biosciences, Mountain View, CA, USA). Approximately 10,000 cells were detected for each sample. Cytogram analysis was done using the Cell Quest software. The experiment was performed in triplicate to confirm the results.

Table S15. Table representing Caspase 3/7 level (%) in different samples.

[25] Gaussian03 program: M. J. Frisch, G. W. Trucks, H. B. Schlegel, G. E. Scuseria, M. A. Robb, J. R. Cheeseman, J. A., Jr. Montgomery, T. Vreven, K. N. Kudin, J. C. Burant, J. M. Millam, S. S. Iyengar, J. Tomasi, V. Barone, B. Mennucci, M. Cossi, G. Scalmani, N. Rega, G. A. Petersson, H. Nakatsuji, M. Hada, M. Ehara, K. Toyota, R. Fukuda, J. Hasegawa, M. Ishida, T. Nakajima, Y. Honda, O. Kitao, H. Nakai, M. Klene, X. Li, J. E. Knox, H. P. Hratchian, J. B. Cross, V. Bakken, C. Adamo, J. Jaramillo, R. Gomperts, R. E. Stratmann, O. Yazyev, A. J. Austin, R. Cammi, C. Pomelli, J. W. Ochterski, P. Y. Ayala, K. Morokuma, G. A. Voth, P. Salvador, J. J. Dannenberg, V. G. Zakrzewski, S. Dapprich, A. D. Daniels, M. C. Strain, O. Farkas, D. K. Malick, A. D. Rabuck, K. Raghavachari, J. B. Foresman, J. V. Ortiz, Q. Cui, A. G. Baboul, S. Clifford, J. Cioslowski, B. B. Stefanov, G. Liu, A. Liashenko, P. Piskorz, I. Komaromi, R. L. Martin, D. J. Fox, T. Keith, M. A. Al-Laham, C. Y. Peng, A. Nanayakkara, M. Challacombe, P. M. W. Gill, B. Johnson, W. Chen, M. W. Wong, C. Gonzalez and J. A. Pople, Gaussian 03, revision D.02; Gaussian, Inc.: Wallingford, CT, **2004.**

[26] B3LYP hybrid functional: (a) R. G. Parr, W. Yang, Density Functional Theory of Atoms and Molecules; Oxford University Press: New York, 1989; (b) C. Lee, W. Yang and R. G. Parr, *Phys. Rev. B* 1988, **37**, 785; (c) A. D. Becke, *Phys. Rev. A* 1988, **38**, 3098-3100; (d) A. D. Becke, *J. Chem. Phys.* 1993, **98**, 5648.

[27] C. Slator, N. Barron, O. Howe and A. Kellett, *ACS Chem. Biol.*, 2016, **11**, 159-171.

Sample	Caspase 3/7 level (%)
Serum starved HCT116 cells	18.4
Healthy HCT116 cells (Control)	8.3
Serum starved HCT116 cells + **PBP1** (2 μM)	9.5
Serum starved HCT116 cells + **PBP2** (2 μM)	31
Serum starved HCT116 cells + **PBP1** (5 μM)	5.1
Serum starved HCT116 cells + **PBP2** (5 μM)	44.7

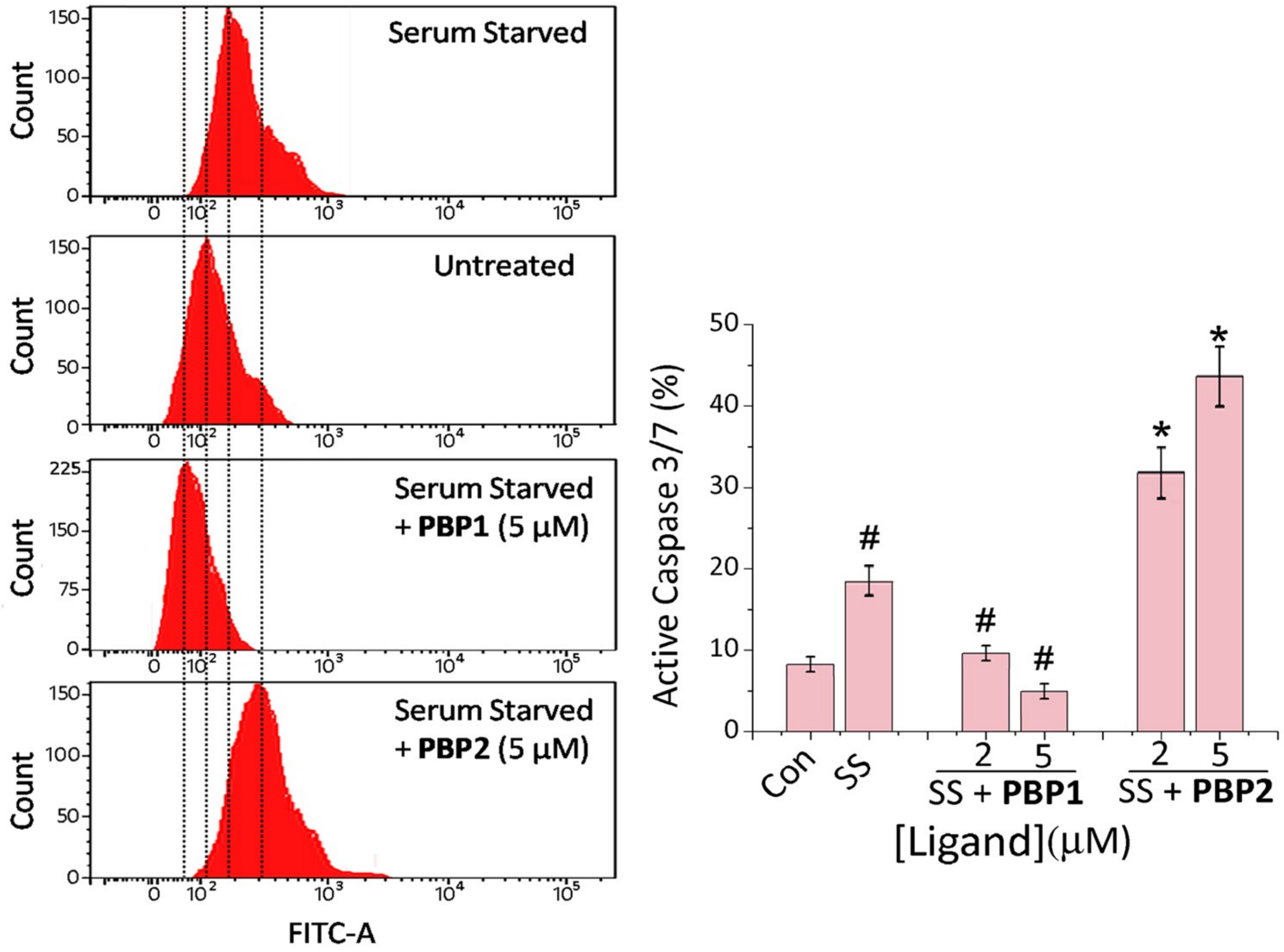

Fig. S34 Flow cytometry histograms in the presence and absence of 5 μM of **PBP1** and **PBP2** in serum starved HCT116 cells (Left). Shift of histograms to the right indicates an increase in caspase 3/7 active cells labeled with FAM. Graphical representation showing the level of active caspases 3/7 in control (Con), Serum starved (SS) and ligand **PBP1** and **PBP2** treated after serum starvation (SS) of cells (Right).

Data shown here as mean ± SD. *P < 0.05, #P < 0.01, versus untreated healthy control cells. An increase in the level of caspases 3 and 7 corresponds to enhanced apoptosis and a decrease in the level of caspases 3 and 7 indicates healthy cells. These results indicate that **PBP1** decreases the level of active caspases 3/7 and **PBP2** induces apoptosis by activating caspases 3/7.
